# Transcriptome analysis reveals the genetic basis underlying the development of skin appendages and immunity in hedgehog (*Atelerix albiventris*)

**DOI:** 10.1038/s41598-020-70844-y

**Published:** 2020-08-18

**Authors:** Hui-Ming Li, Bi-Ze Yang, Xiu-Juan Zhang, Hai-Ying Jiang, Lin-Miao Li, Hafiz Ishfaq Ahmad, Jin-Ping Chen

**Affiliations:** Guangdong Key Laboratory of Animal Conservation and Resource Utilization, Guangdong Public Laboratory of Wild Animal Conservation and Utilization, Guangdong Institute of Applied Biological Resources, Guangdong Academy of Science, Guangzhou, 510260 China

**Keywords:** Genetics, Molecular biology

## Abstract

The expression of hair features is an evolutionary adaptation resulting from interactions between many organisms and their environment. Elucidation of the mechanisms that underlie the expression of such traits is a topic in evolutionary biology research. Therefore, we assessed the de novo transcriptome of *Atelerix albiventris* at three developmental stages and compared gene expression profiles between abdomen hair and dorsal spine tissues. We identified 328,576 unigenes in our transcriptome, among which 4,435 were differentially expressed between hair- and spine-type tissues. Dorsal and abdomen skin tissues 5 days after birth were compared and the resulting DEGs were mainly enriched in keratin filament, epithelium cell differentiation, and epidermis development based on GO enrichment analysis, and tight junction, p53, and cell cycle signaling pathways based on KEGG enrichment analysis. *MBP8*,* SFN*,* Wnt1* and *KRT1* gene may involve in the development of hedgehog skin and its appendages. Strikingly, DEGs in hair-type tissues were also significantly enriched in immune-related terms and pathways with hair-type tissues exhibiting more upregulated immune genes than spine-type tissues. Our study provided a list of potential genes involved in skin appendage development and differentiation in *A. albiventris*, and the candidate genes provided valuable information for further studies of skin appendages.

## Introduction

Perceiving and responding to life-threatening signals and regulating their own morphological characteristics constitute a fundamental challenge for all mammals. Evolution has shaped the ability of organisms to adapt skin appendages to increase their chances of survival. Accordingly, a variety of appendages have evolved on the skin of mammals, either to assist organisms in carrying out specific behaviors or to protect them from predators and pathogenic bacteria^1,2^.

Compared with animals that possess passive defense adaptations such as feathers, scales, etc., the hedgehog has evolved into an unusual, nocturnal, spine-covered mammal. Spines potentiate attacking power and enhance the role of skin appendages as a defense mechanism. The hedgehog progresses through a series of well-defined stages during its life cycle, from embryonic, to non-spine, to with-spine stages^3^. These transitions are governed by tightly regulated gene expression at pre-transcriptional, epigenetic, and translational levels. Spine- and hair-type skin tissues from the hedgehog thus offer a natural model to analyze the genomic basis for the evolution of epidermal appendage formation.

Development of hair follicles on a skin appendage with a complex growth cycle is characterized by anagen, catagen, and telogen stages, and many key signaling pathways and genes are involved in their regulation^4–6^. According to many studies, the WNT/β-catenin signaling pathway in the dermis may be the first dermal signal^7–9^. Sonic hedgehog (SHH) is another secreted protein in the follicular placode that plays a major part in epithelial-mesenchymal signaling^10,11^. SHH depends on WNT signaling and is required for the proliferation of follicular epithelium and development of dermal condensate into dermal papilla^4^. In addition, many key genes related to skin appendage development have been identified; *TGFaR* (epidermal growth factor receptor) and transcription factor *ETS2* regulate hair follicle shape and are responsible for hair follicle architecture and wavy hair^12,13^, and *HOXC13* and *KRT75* control hair shaft differentiation^14–17^. Further, *EDA* and *EDAR* interact with members of the bone morphogenetic protein (*BMP*) family, some of which inhibit follicle development to establish follicle patterning^2,18–20^.

The abovementioned studies primarily focused on mammalian hairs and scales, little is known about changes in gene expression during the development of spine appendages in mammals. Therefore, we aimed to perform a detailed analysis of the transcriptome of skin from abdomen hair and dorsal spines of the hedgehog (*Atelerix albiventris*) by comparing groups of transcripts differentially expressed at different hedgehog developmental stages. We identified key candidate genes related to the development and differentiation of skin appendages whose importance should be verified in future studies. Nonetheless, the presented novel information will be widely applicable in many fields, such as skin disease and skin immune genes, and provide insight into the molecular mechanisms of skin appendage development in general.

## Results

### Illumina sequencing and de novo assembly

**Figure 1:**
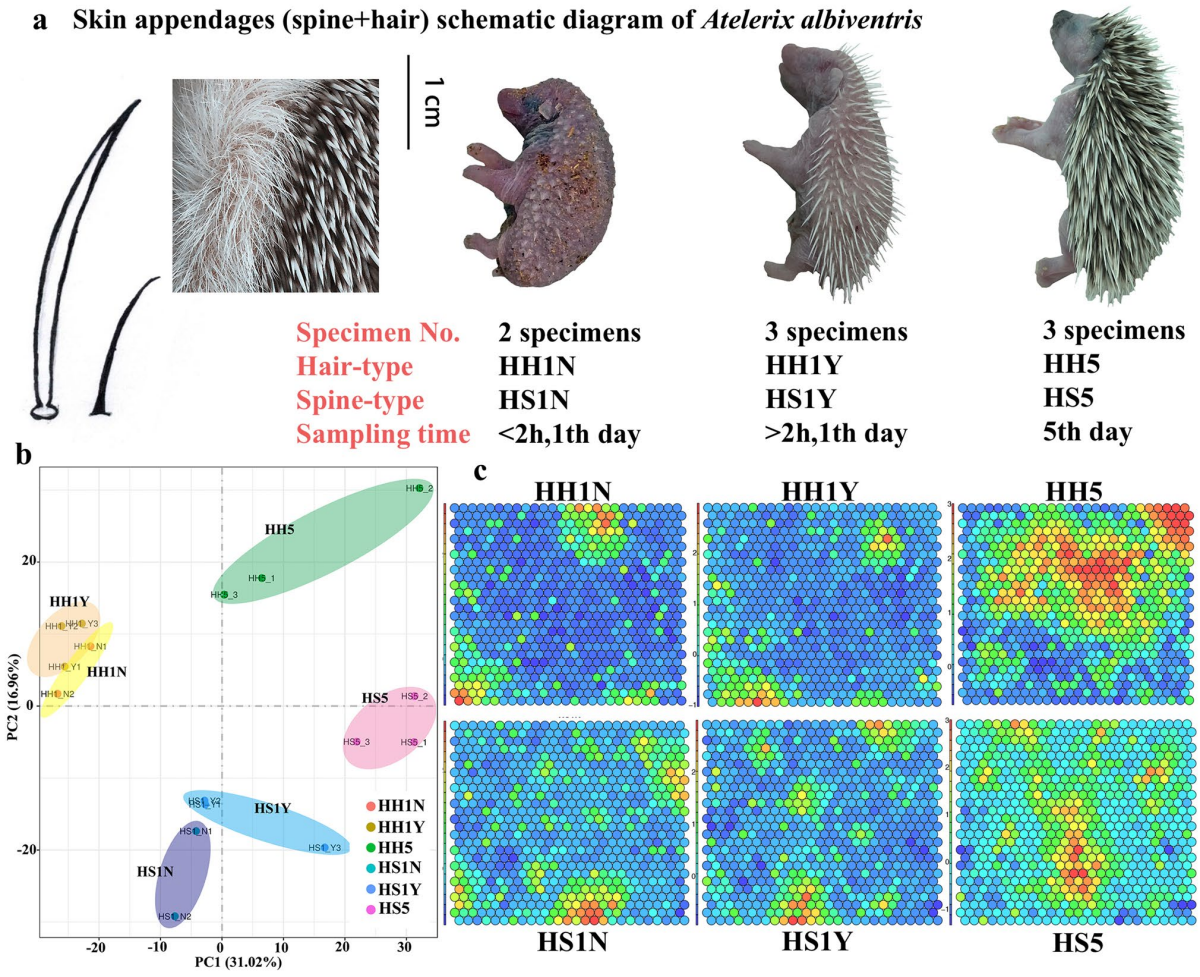
Phenotypes of skin appendages from 3 developmental stages of *Atelerix albiventris*. (**a**) Different phenotypes from 3 different developmental stages of *A. albiventris*; (**b**) Principal components analysis (PCA); (**c**) Gene expression-specific and phenotype-specific gene-trait correlation analysis based on self-organizing feature map module analysis (SOM). (**b**, **c**) were generated using RStudio v1.2.1335: https://rstudio.com/products/rstudio/.

To identify the transcriptome and molecular mechanisms governing skin appendage development and differentiation, we analyzed temporal changes in transcript abundance of *A. albiventris* (Fig. 1a). RNA sequencing generated 82.2 ± 6.0 G (mean ± SD) read pairs for 16 skin tissue samples (Supplementary Table 1). After quality trimming, 96.8 ± 0.4% of reads were retained, indicating a high-quality dataset (> 90% reads with ≥ Q30). The de novo assembly using Trinity revealed 328,576 unigenes, which were used for subsequent analysis. We evaluated biological reproducibility by individually comparing biological replicates through principle component analysis (PCA) (Fig. 1b). As expected, similar gene expression patterns in both hair- and spine-type samples were observed. PCA analysis also demonstrated more separation between the two skin appendage types. A neural network graph based on self-organizing feature map (SOM) analysis revealed dynamic transcriptional changes at the individual stages of appendage development in *A. albiventris* (Fig. 1c).

### Functional annotation and classification of unigenes

Use of the NR and Swiss-Prot databases yielded reliable protein annotations for approximately 42% of unigenes. Absolute unigenes were annotated using seven major public protein databases (Table 1), of which the greatest number of matches (68.3%) was obtained using the NT database. Of 328,576 unigenes assembled, 243,444 genes (74.1%) exhibited a positive match against at least one database. Thus, our gene annotation results from *A. albiventris* transcriptome were considered high quality.

**Table 1:** Summary of the functional annotation of assembled hedgehog unigenes with public protein databases using BlastX cut-off E-value of 1E-5.

	Number of unigenes	% Annotated unigenes
Annotated in NR	54,658	16.63
Annotated in NT	224,390	68.29
Annotated in KO	36,283	11.04
Annotated in SwissProt	67,656	20.59
Annotated in PFAM	70,833	21.55
Annotated in GO	70,833	21.55
Annotated in KOG	24,944	7.59
Annotated in all databases	12,570	3.82
Annotated in at least one database	243,133	73.99
Total unigenes	328,576	100

**Figure 2:**
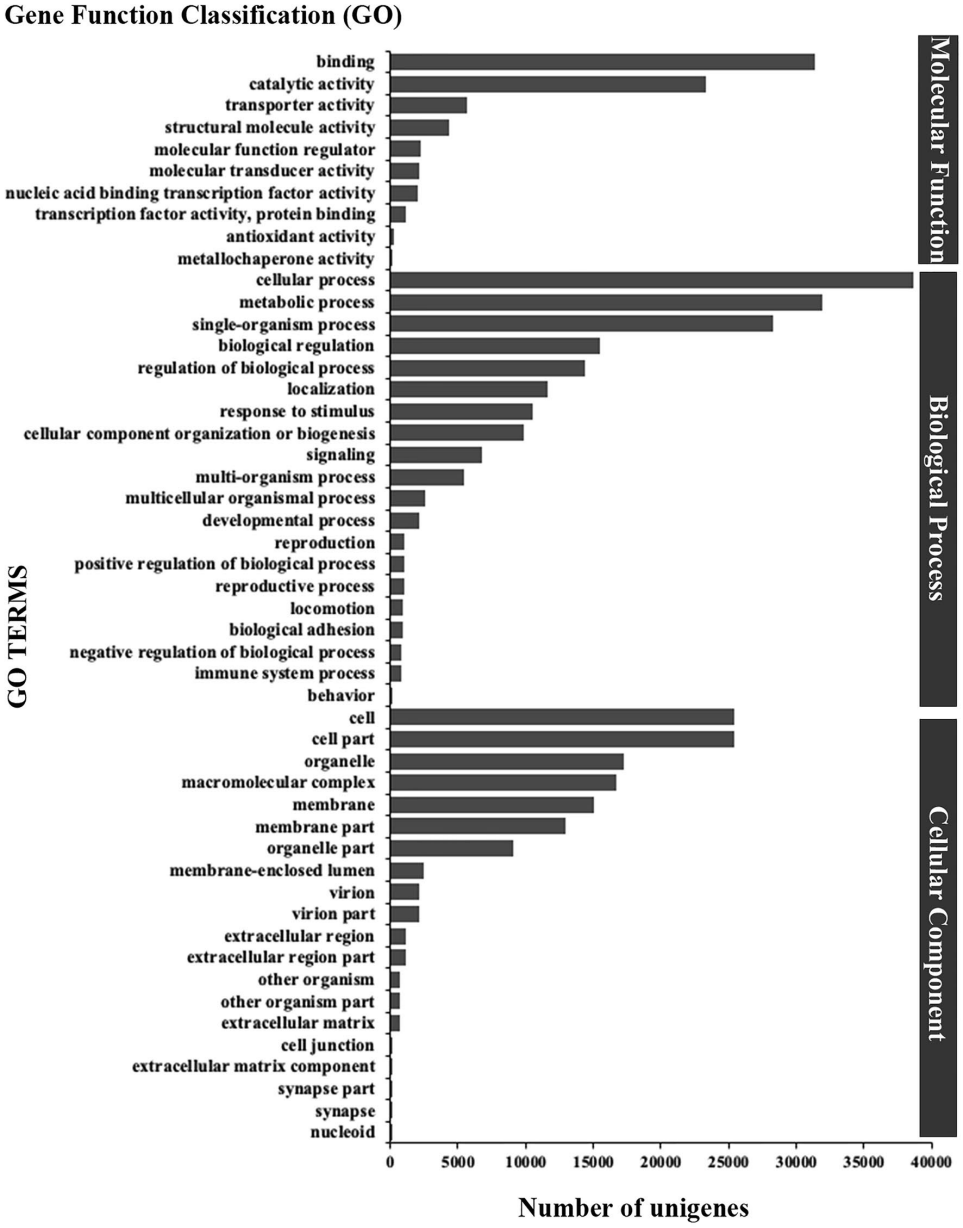
GO classification of assembled unigenes in *Atelerix albiventris*.

In our study, 70,833 unigenes were assigned to 56 sub-categories of GO terms belonging to the following three main categories: biological process (BP), cellular component (CC), and molecular function (MF). These main categories included 20, 20, and 10 sub-term categories, respectively (Fig. 2). The most enriched GO terms were related to cellular and metabolic processes (BP), cell and organelle (CC), and binding and catalytic activity (MF). Importantly, we found some GO terms (level 4) in the BP category related to skin appendage development and differentiation, including (positive/negative) regulation of cell differentiation, regulation of cell proliferation, reproductive structure development, cell development, and ovarian follicle cell development.

**Figure 3:**
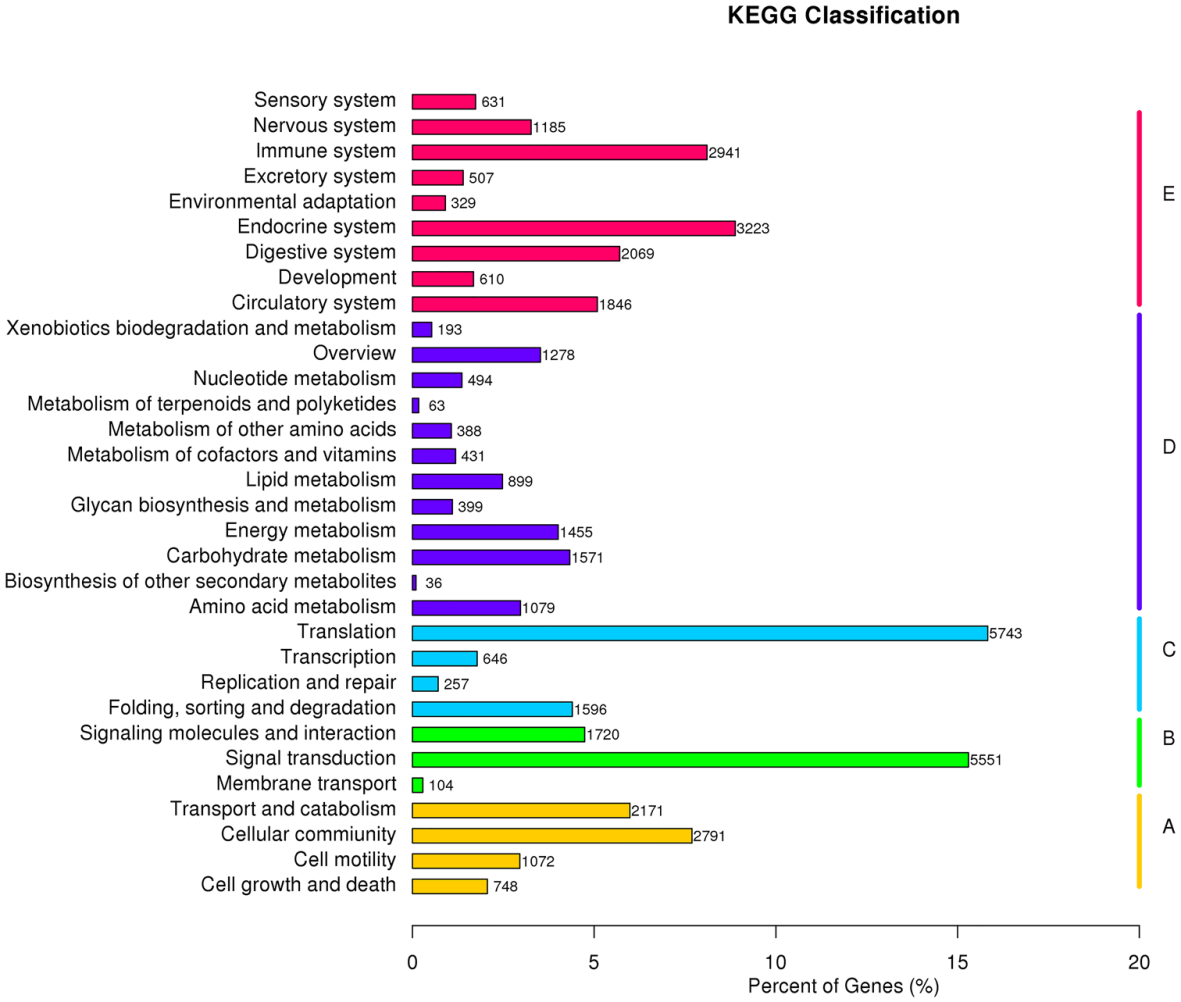
KEGG classification of assembled unigenes in *Atelerix albiventris*. (A) cellular processes; (B) environmental information processing; (C) genetic information processing; (D) metabolism, (E) organismal systems.

Furthermore, 36,283 unigenes were mapped to 232 biological signaling pathways in KEGG database. Of which 15, 30, 22, 98, and 72 unique KEGG pathways represented in five KO hierarchies (Fig. 3). The most genes were annotated in translation pathways (5,743 unigenes), next by signal transduction (5,551 unigenes). In addition, there were some unigenes and pathways were related to skin appendage development and differentiation, including Rap1 (814 unigenes), Wnt (355 unigenes), Hippo (626 unigenes), TGF-β (206 unigenes), and Notch (171 unigenes) signaling pathways.

### Analysis of differentially expressed genes in skin appendage tissues

**Figure 4:**
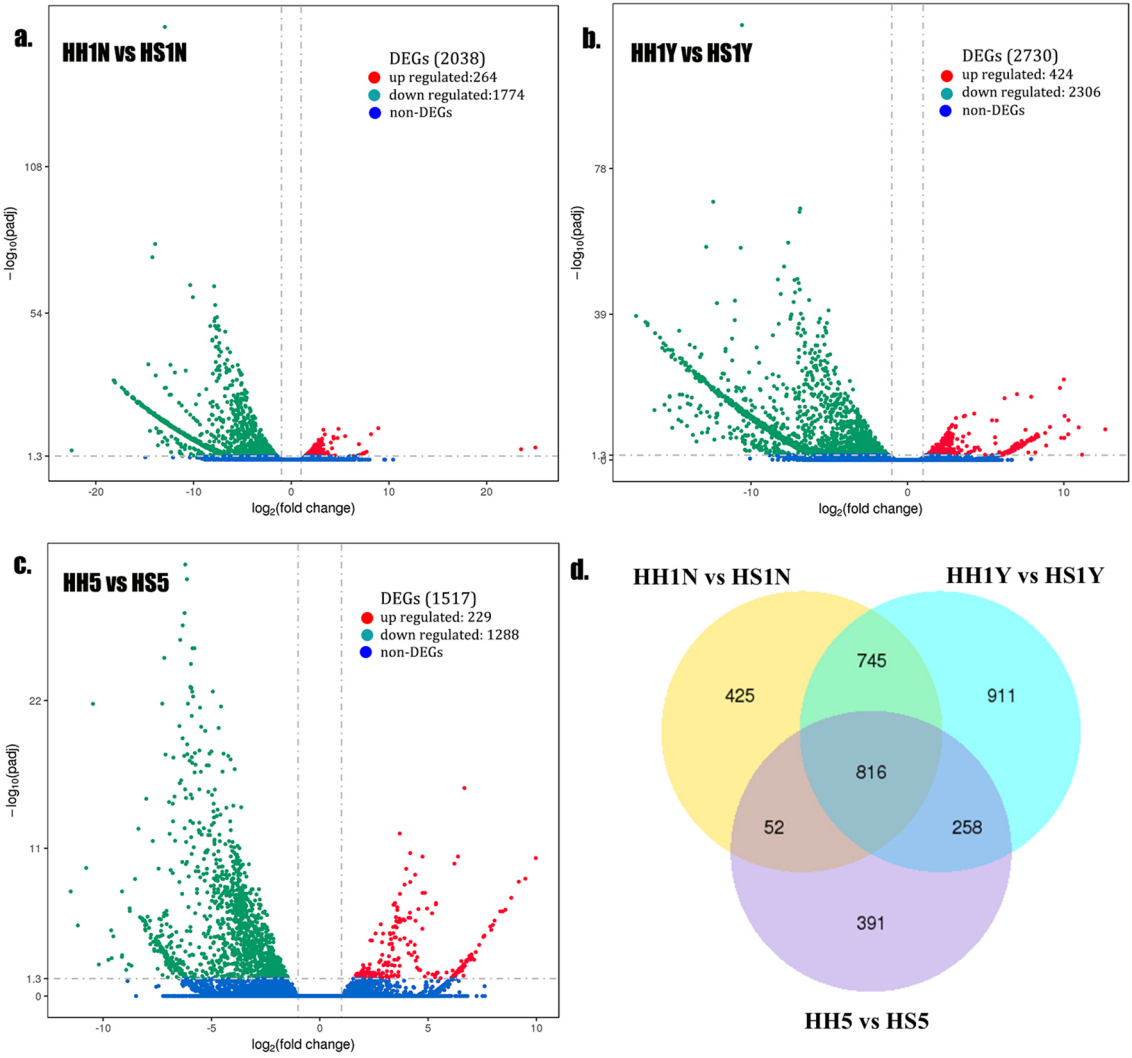
Differential expression analysis of hair- and spine-type tissues in *Atelerix albiventris*. Volcano plot of DEGs for (**a**) ‘HH1N versus HS1N’, (**b**) ‘HH1Y versus HS1Y’, (**c**) ‘HH5 versus HS5’. (**d**) Venn diagram showing co-DEGs among different tissues in *Atelerix albiventris*. (**a**–**c**) of DEGs analysis were generated using Novomagic website, Novogene Bioinformatics Institute, Beijing, China: https://magic.novogene.com.

Analysis of DEGs in skin appendage tissues finally identified 4,435 DEGs in the three developmental stages, and 816 DEGs shared by the two types of tissues (Fig. 4a–c). As shown in the Venn diagram (Fig. 4d), more DEGs were found in tissues with spines (HH1Y and HS1Y) than in tissues without spines (HH1N and HS1N).

**Figure 5:**
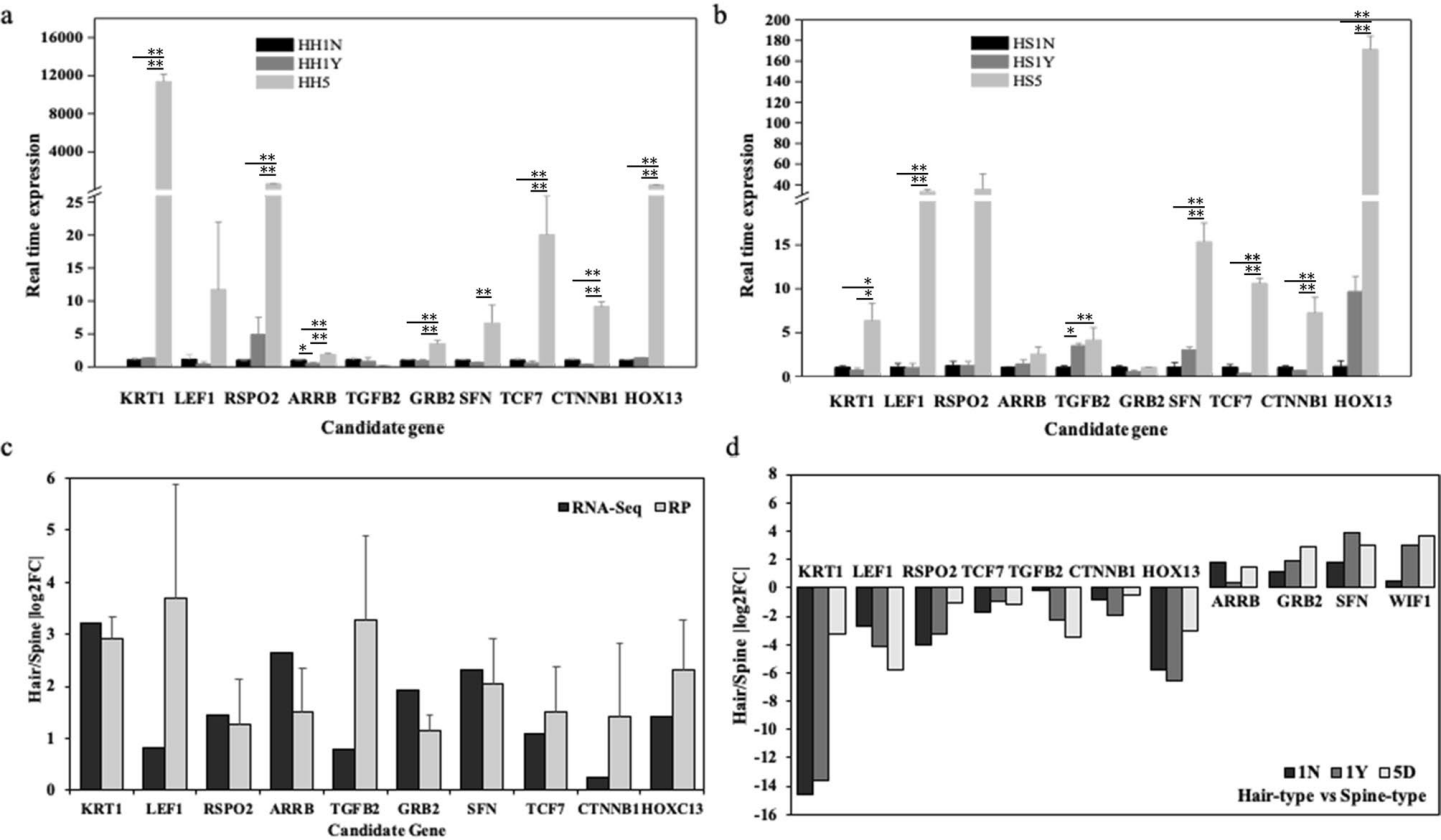
Quantitative real-time PCR analysis. (**a**) The real time expression of 10 DEGs for hair-type tissue in three development stage; (**b**) The real time expression of 10 DEGs for spine-type tissue in three development stage; (**c**) qPCR confirmation of 10 DEGs identified by RNA-seq in stage III tissues; (**d**) Relative expression levels of 10 DEGs in tissues at 3 developmental stages. Bars represent the relative expression levels of unigenes in stage III tissues normalized with respect to the internal control GAPDH. Error bars represent the standard error of three biological replicates. Bars with asterisk symbol indicate statistical differences (*p < 0.05; **p < 0.01).

To validate expression patterns indicated by the transcriptome data, 10 differentiation-related DEGs were selected for RT-qPCR analysis including *KRT1*, *LEF1*, *RSPO2*, *ARRB*, *TGFB2*, *GRB2*, *SFN*, *TCF7*, *CTNNB1*, *HOXC13*, and *WIF1* (Supplementary Table 2). Expression trends determined by RT-qPCR significantly correlated with the RNA-Seq data (Fig. 5a–c). Expression of these 10 genes at the three developmental stages was also analyzed, revealing expression patterns similar to those determined by RNA-sequencing. Expression of *LEF1*, *TGFB2*, *SFN*, and *WIF1* at stages I–III increased significantly. However, expression of *KRT1* and *RSPO2* gradually decreased (Fig. 5d). Overall, both approaches confirmed the observed DEG trend patterns, indicating the accuracy of the transcriptome data and de novo RNA-Seq data.

### Differentially expressed genes related to skin appendage development

In order to further understand the spine development mechanism of the hedgehog, we conducted DEG analysis before and after spine development. In total, 28 DEGs were identified between HS1N and HS1Y, among them, 11 genes were downregulated, and the remaining 17 genes were upregulated (Supplementary Table 3). Based on GO and KEGG annotation, we found one downregulated gene of unknown open reading frame (LOC103122410) related to cell differentiation and multi-cellular organismal development in the BP category, and one downregulated keratin-associated protein like gene (LOC103118355) related to keratin filament.

In order to identify genes that activate the development of hedgehog abdominal hair, we analyzed DEGs before and after the occurrence of abdominal hair. A total 82 DEGs were identified between HH1N and HH1Y, of which 62 genes were downregulated, and the remaining 20 genes were upregulated (Supplementary Table 4). Notably, LOC103122410 was also present in these DEGs; in addition, keratin gene *KRT2* was highly expressed in HH1Y. We speculate that these genes play an important role in the development of hedgehog abdominal hair and spines.

### Differentially expressed genes related to skin appendage differentiation

We identified 1,517 DEGs by comparing the gene expression profiles of HS5 and HH5 tissues in *A. albiventris* 5 days after birth (Supplementary table 5). We screened more than 20 genes related to cell differentiation, cell proliferation and development, including 11 main structural proteins: *KRT1*,* KRT2*,* KRT6A*,* KRT75*,* KRT16*,* KRTAP 9-2*,* KRTAP 13-1*,* KRTAP13-2*,* KRTAP 19-2*,* KRTAP 19-3*,* TCHH*, and 9 regulatory genes: *NOTCH*,* TGFB1*,* FGF*,* BMP3*,* BMP8*,* WNT3*,* WNT10*,* OTX1 and DSC2*. These genes might be indicative of hedgehog transcriptome involvement in spine and hair differentiation.

**Figure 6:**
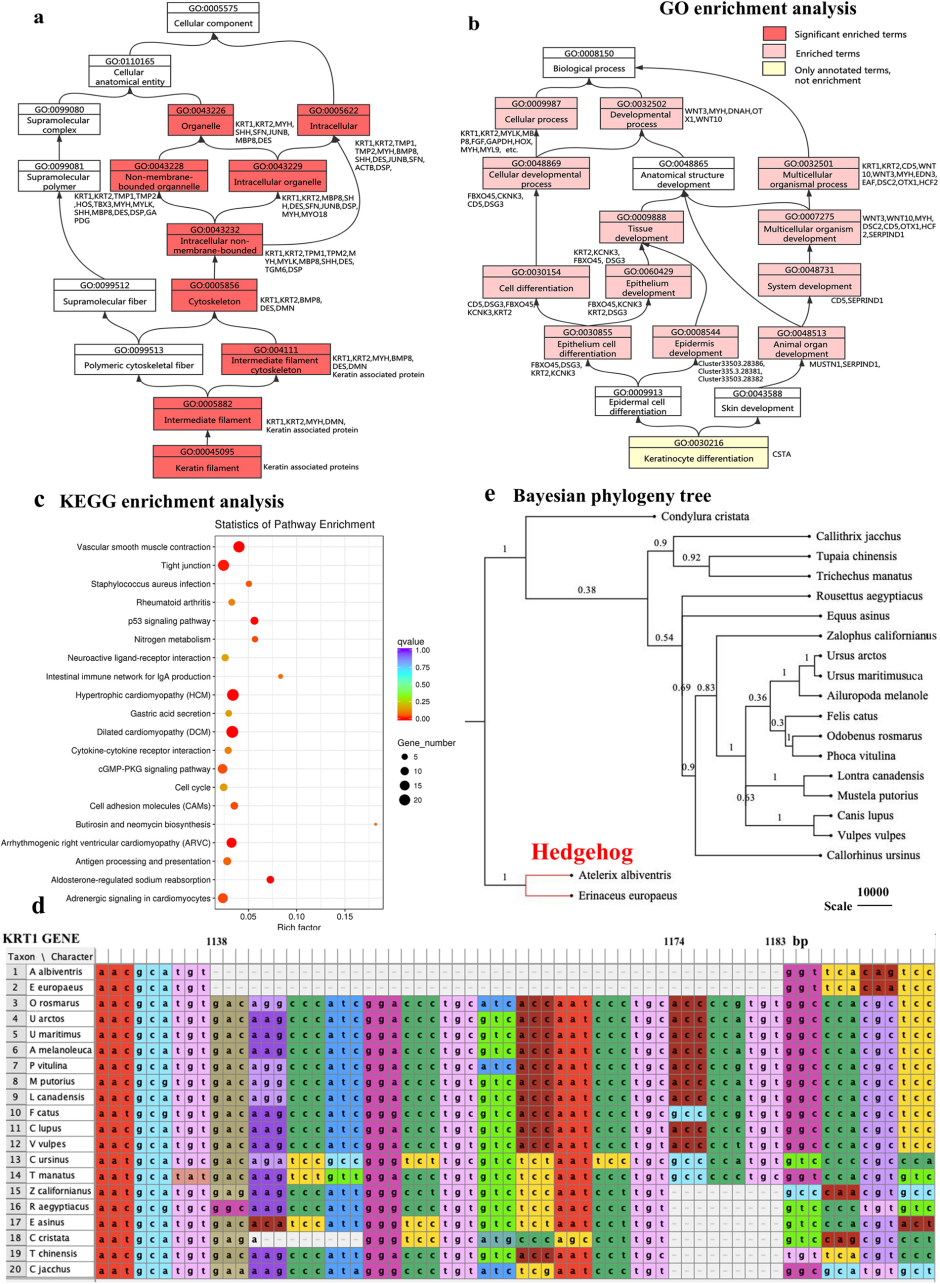
KEGG and GO enrichment analysis of DEGs related to differentiation of KRT1 gene. (**a**, **b**) Directed acyclic graph related to skin appendage development and differentiation in GO enrichment analysis. (**c**) Dot plots of enriched KEGG pathways. (**d**) Multiple sequence alignment of *KRT1* gene from 20 species. (**e**) Bayesian phylogenetic tree with *KRT1* sequences from 20 species. (**a**, **b**) were generated using website of ProcessOn: https://www.processon.com/; (**c**) was generated by Novomagic website, Novogene Bioinformatics Institute, Beijing, China: https://magic.novogene.com; (**d**) was generated by Mesquite v3.5.1: https://www.mesquiteproject.org/; (**e**) was generated by BEAST v1.6.1: https://beast.community/.

Based on annotation of the *A. albiventirs* transcripts with the GO database, the DEGs were significantly enriched in keratin-related terms. Keratin filament is an important component of skin appendages; the main DEGs involved in this process included *KRT1*,* KRT2*,* MYH*,* DES*,* BMP8*,* SHH*, and several *KRTAP* genes (Fig. 6a,b). In addition, *CSTA* was involved in the biological process of keratinocyte differentiation. In a directed acyclic graph diagram associated with this term, cell differentiation, epithelial cell differentiation, and epidermis development were enriched, and the main DEGs involved in this process included *KRT1*,* KRT2*,* MBP8*,* FGF*,* Wnt10*, and *MYH* (Fig. 6b). The only significantly enriched KEGG pathways among all DEGs comparing HH5 and HS5 tissues were tight junction and p53 signaling pathways (Fig. 6c; Table 2). In addition, eight pathways related to skin appendage development and differentiation were enriched, including cell cycle, MAPK, Rap1, Hippo, VEGF, TGF-beta, and PPAR signaling pathways. It is noteworthy that *SFN*,* BMP8*,* Wnt3*,* Wnt10*,* MYH*, and *SFN* were found in both annotation results, suggesting they are closely related to differentiation of keratinocytes and epidermal cells.

**Table 2:** The candidate pathway and genes related to spine and hair development and differentiation.

ID	Pathway	*p* value	Representative genes
ko04115	P53	< 0.001	SFN, RCHY1_PIRH2
ko04530	Tight junction	< 0.001	ACTB_G1, MYL9, MYH, CLDN, SHROOM
ko04110	Cell cycle	0.014	SFN
ko04010	MAPK	0.291	SFN, HSPA1_8, CACNA1H, CACNA1I, HSPB1
ko04015	Rap1	0.490	FGF, ACTB_G1, DRD2, CSF1R, RAPGEF3, ADCY8
ko04390	Hippo	0.525	ACTB_G1, BMP8, WNT3, WNT10
ko04510	Focal adhesion	0.705	ACTB_G1, MYL9, FLNA, PAK6, MYLK, PARV, SPP1
ko04370	VEGF	0.839	HSPB1
ko04350	TGF-beta	0.855	BMP8
ko03320	PPAR	0.999	UBC

**Figure 7:**
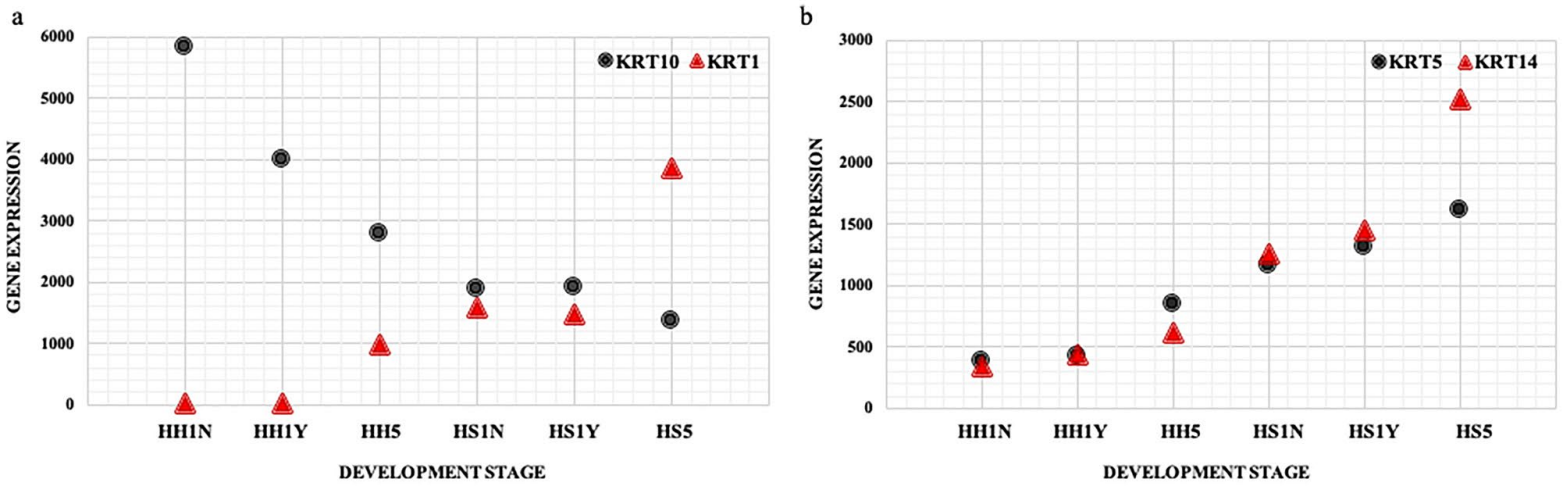
The expression of four DEGs keratin genes in two types tissue. (**a**) KRT1 and KRT10 expression; (**b**) KRT5 and KRT14 expression.

In our data, keratin-related genes were highly abundant and also significantly overexpressed in the dorsal spine-type tissues. In the hedgehog transcriptome, more than 3,000 transcripts were annotated to 5 keratin genes (*KRT1*,* KRT2*,* KRT5*,* KRT10* and *KRT14*) and approximately 20 keratin-associated proteins. *KRT1* and *KRT10* (Fig. 7a), *KRT5* and *KRT14* (Fig. 7b) often form heterodimers in the epidermis and interfollicular epidermis of hair. However, in our transcriptome data, *KRT1* and *KRT10* were not co-expressed, *KRT1* was significantly up-regulated in spine-type tissue, while *KRT10* was down-regulated. Through alignment of *KRT1* gene sequences from 20 species, we found that the hedgehog *KRT1* gene sequence has a deletion of 45 bp compared with other species (Fig. 6d). The two hedgehog species were isolated into one clade in the bayesian phylogenetic tree (Fig. 6e), suggesting that *KRT1* may be one of the important gene involved in the development and differentiation of hedgehog skin appendages.

### Immune-related genes in epidermis of hedgehog

**Figure 8:**
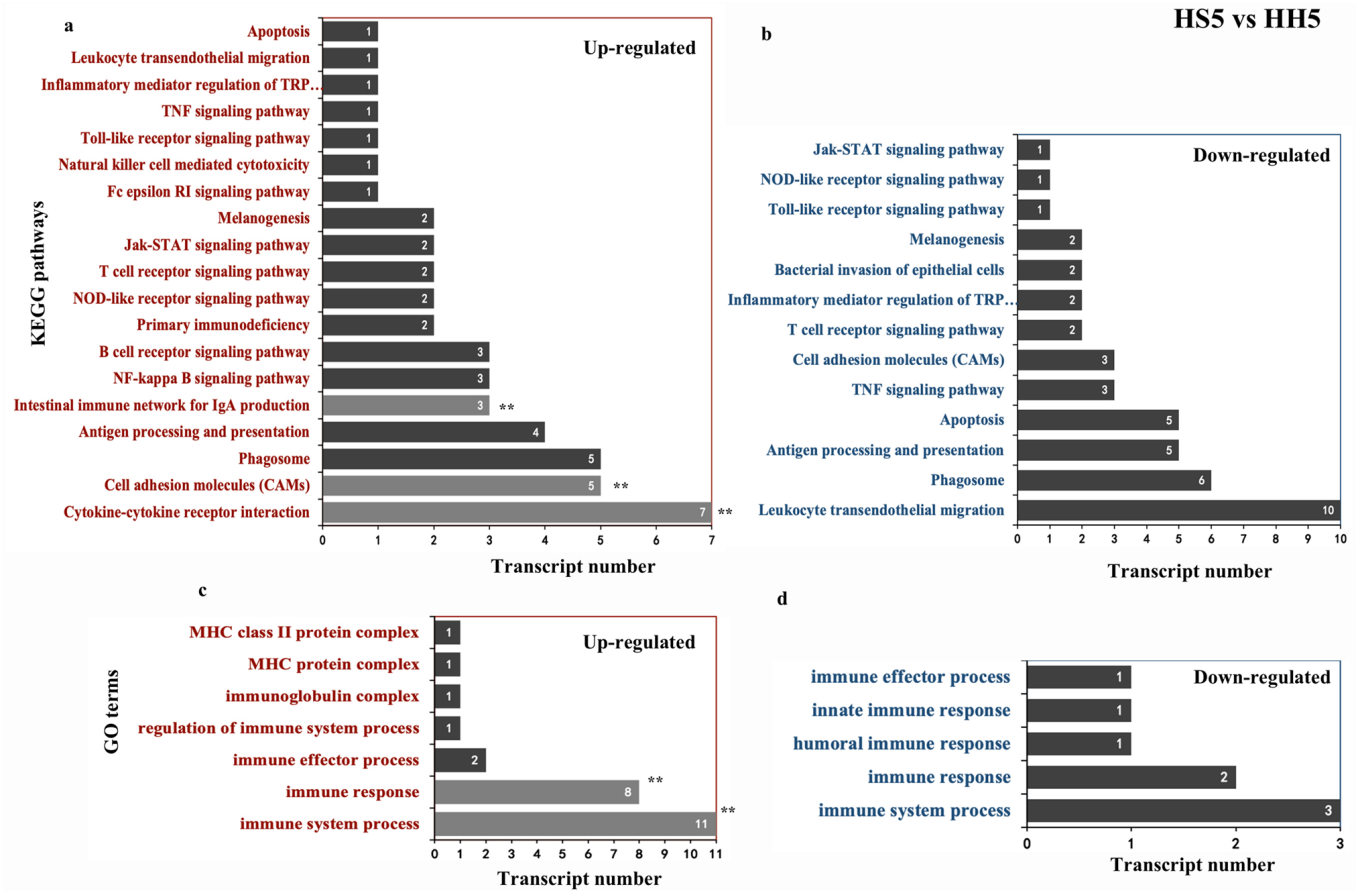
KEGG and GO enrichment analysis of DEGs related to immunity. (**a**, **b**) up- and downregulated KEGG signaling pathways and key genes related to immunity. (**c**, **d**) up- and downregulated GO terms and key genes related to immunity.

In this study, we also found many immune-related genes comparing HH5 and HS5. After KEGG enrichment analysis, we found no significant enrichment of immune-related pathways in spine-type tissues, and fewer immune-related pathways and genes than in hair-type tissues. Hair-type tissues exhibited 19 enriched immune-related pathways, of which cytokine-cytokine receptor interaction, intestinal immune network for IgA production and cell adhesion molecules (CAMs) pathway were significantly enriched, and including 6 immune-related genes: *CD27*,* CCR7*,* CCL5*,* LTB*,* PIGR* and *MHC2* (Fig. 8a,b). In addition, we observed similar GO enrichment analysis results. The number of terms and genes enriched in hair-type tissues was significantly higher than in spine-type tissues, of which immune response and immune system process terms were enriched considerrably, and 6 genes related to immunity: *MHC2*,* CCL5*,* END3*,* LTB*,* CXCL5* and *C1QA* (Fig. 8c,d).

## Discussion

The genetic basis of morphological variation, both within and between species, provides a major topic in evolutionary biology. In mammals, the development of skin appendages such as hair, tooth, and scale involves complex interactions between the epidermis and the underlying mesenchyme as part of an established hierarchical morphogenetic process^21^. Specifically, mammals develop a coat containing many distinct types of hair. Such diversity is associated with molecular and signaling pathways that drive formation and induction in a specific spatial and temporal manner^22^. Reciprocal interactions between epithelial and mesenchymal tissues constitute a central mechanism that determines the location, size, and shape of organs^23^.

In the current study, we aimed to explore the genetic basis for hedgehog skin appendage differentiation and development and the resulting expression of the spine trait. We identified 328,576 unigenes in our transcriptome, all of which were annotated in 7 databases (Table 1). Taken together, our de novo assemblies revealed higher quality compared with previous studies^24,25^ that formed the foundation of all our subsequent analyses. According to GO and KEGG annotation analysis, a total of 70,833 and 36,238 unigenes were mapped to 56 sub-categories and 232 biological pathways, respectively (Figs. 2, 3). We identified some of the key pathways involved in skin development; these annotations may provide a valuable resource for further understanding the specific functions and pathways in *A. albiventris*.

Newborn hedgehogs begin to develop hair/spines approximately 2 h after birth. We found 6 shared genes (*APOE*,* COX2*,* COX3*,* FCN*,* RP-L11e*,* SH3GL*) through analysis of DEGs before and after hair/spine development, indicating that these genes are essential regulatory genes in the development of skin appendages, whether hair or spine. Further, we compared the gene expression profiles of spine- and hair-type tissues to systematically assess key regulatory genes for spine and hair differentiation in *A. albiventris*. *SFN*, upregulated in spine-type tissues, is a regulator of mitotic translation that interacts with a variety of translation and initiation factors^26^, is enriched in the p53 signaling pathway, and has a role in keratinocyte differentiation and skin barrier establishment in the BP category (GO). *SFN* plays an important role in maintaining hair follicle development, especially affecting the formation of hair shaft structure^27^. Bu et al.^28^ determined that SFN gene and protein were significantly highly expressed in the thicker, longer, and harder skin of wool, indicating that *SFN* was involved in the regulation of wool character development. In addition, we identified *FGF* as the upregulated DEG enriched in the MAPK and Rap1 signaling pathways. These pathways often act together by forming signaling loops during organogenesis^29^ and induce the most fundamental biological processes, such as the formation of periodic patterns. Hebert et al. (1994) demonstrated that *FGF* plays an important role in regulation of the hair cycle growth, and functions as an inhibitor of hair elongation by promoting progression from anagen stage, the growth phase of the hair follicle, to catagen stage, the apoptosis-induced regression phase^16,30^. Hence, we believe that *SFN* and *FGF* may be important regulators that affect spine development and differentiation in *A. albiventris*.

At present, keratin and keratin associated proteins are the main structural proteins in model organisms^31,32^, which determine the basic properties of hair, such as fineness, length, hardness and luster, etc^33^, and in the process of hair formation, a series of keratin genes also determine the process of hair follicle cell differentiation^34^. In addition, many skin and hair-related diseases are also associated with mutations and expression of keratin family genes^35–37^. In this transcriptome study, it was found that there were significant differences in keratin gene expression in different skin regions at different development stages, such as *KRT1*,* KRT2*,* KRT5*,* KRT10*,* KRT14*,* KRT6A*,* KRT75*,* KRATP9-2*,* KRTAP13-1*,* KRTAP19-2*, etc. Parson et al.^38^ and Rogers et al.^39^ found that the differential expression of keratin and keratin associated proteins was related to the fineness and density of hair at different development stages. Khan et al.^40^ found that the unique hair-related phenotypes, such as scales (armadillo) and spines (hedgehog), were correlated changes in the expression variation probably also influences hair diversification patterns. And the number and size of differences in gene family replication, loss and pseudogenization are very important for the evolutionary history of mammals^41,42^.

We also compared *KRT1* gene sequences of 20 mammals with that of the hedgehog, in which we identified a 45-bp deletion in the latter. Keratins, the major structural proteins of epithelia, are a diverse group of cytoskeletal scaffolding proteins that form intermediate filament networks and provide structural support to keratinocytes that maintain skin integrity^43^. In general, *KRT1* is highly conserved in mammals, however, molecular defects in keratin intermediate filament-related genes can cause keratinocyte and tissue-specific fragility, accounting for a large number of genetic disorders in skin and its appendages^44,45^. Therefore, whether the deletion phenomenon in hedgehog *KRT1* has special significance for spine development and differentiation in the hedgehog warrants further research and verification.

Lastly, we analyzed immune-related genes in the skin transcriptome of *A. albiventris*. We found that hair-type skin had more immune-related genes than spine-type skin. Choo et al. found that interferon epsilon (*IFNE*), which was exclusively expressed in epithelial cells and was important to mucosal immunity, was pseudogenized in pangolins. They proposed that scale development provided protection against injuries or stress and reduced pangolin vulnerability to infection, thus protection by scales on the pangolin body compensated for the low immunity of this species to a certain extent^46^. From our current data, we speculate that there may be significant differences in the immune function of skin with different appendage types, and that the occurrence of spines may be an innovative physical protection for dorsal skin with relatively low immunity.

## Conclusions

In the current study, we conducted a comprehensive transcriptome analysis of *A. albiventris* to explore the genetic basis for the growth of skin appendages. Transcriptome analysis provided a rich list of unigenes expressed in hair- and spine-type tissues at three different developmental stages, of which 328,576 unigenes and 3,598 DEGs were identified. Candidate genes were identified that are likely involved in the regulation of hair and spine growth and differentiation. The knowledge acquired in this study of the molecular and signaling pathways related to hair and spine expression greatly contributes to the current genetic resources for the hedgehog and mammalian species that harbor shaggy appendages, as well as traits that could be potentially exploited for curing skin diseases of other animals, even humans.

## Methods

### Biological samples

According to observations of the growth characteristics of hedgehogs, hairs and spines on the back and abdomen begin to appear approximately 2 h after birth; therefore, we obtained 8 hedgehogs at 3 different stages of appendage development from a commercial animal farm (Dongguan City, China). Hedgehog hair (HH) and spine (HS) tissues were collected from 2 specimens within 2 h of birth (Stage I, HH1N/HS1N), 3 specimens after 2 h but within the first birth day (Stage II, HH1Y/HS1Y), and 3 specimens 5 days after birth (Stage III, HH5/HS5). Prior to skin sampling, follow the standard operating procedures for euthanasia, conduct euthanasia for experimental animals, minimize the pain of small animals, do not affect the results of animal experiments, and shorten the time of death as far as possible. Sixteen skin tissue samples representing the two types of appendages (abdomen hair-type and dorsal spine-type) were rapidly excised, immediately snap-frozen on dry ice, and stored at − 80 °C until RNA extraction.

### RNA extraction and RNA-seq

Total RNA from each tissue sample was extracted using the RNeasy Kit (Qiagen, Hilden, Germany). RNA purity was determined using a NanoPhotometer spectrophotometer (Implen, Inc., Westlake Village, CA, USA). All unigenes were annotated using the basic local alignment search tool (BLASTX), considering hits with e-values of 1E−5 against seven databases: NCBI non-redundant protein sequences (Nr); NCBI non-redundant nucleotide sequences (Nt); Protein family (Pfam) database; Clusters of Orthologous Groups of Proteins (KOG/COG) database; Swiss-Prot (a manually annotated and reviewed protein sequence database); KEGG Ortholog (KO) database^47,48^; Gene Ontology (GO) database. Differentially expressed genes (DEGs) were identified by comparing gene expression levels between samples (or sample groups). Bowtie2^49^ was used to align clean reads with all unigenes and RSEM^50^ was used to calculated gene expression levels for each sample. Differential gene expression was analyzed using DESeq270 V1.16.1 in RStudio71 V1.0.143 running R72 V3.4.1. All sequencing was conducted by Novogene Bioinformatics Institute (Beijing, China).

### Quantitative real-time reverse-transcription PCR (RT-qPCR)

RNA was extracted from skin appendage tissue samples using TRIzol reagent (Invitrogen, Carslbad, CA, USA). Then, cDNA was synthesized using the Toyobo reverse-transcription kit (Toyobo, Osaka, Japan). Eleven genes related to hair and/or spine development were selected for analysis based on functional annotation data, and fluorescent qPCR primers were designed accordingly (Supplementary Table 2). RT-qPCR was performed in a 20-μL reaction volume, with four technical replicates for each sample, using the TransStart Top Green qPCR SuperMix kit (TransGen Biotech, Beijing, China). Relative gene expression levels were analyzed using the 2^−ΔΔCT^ method^51^. PCR conditions were as follows: pre-denaturation at 95 °C for 10 min; 40 cycles of 15 s at 95 °C (denaturation), 30 s at 58 °C (annealing), and 20 s at 72 °C (extension); and a final melting curve stage from 60 to 95 °C to verify the specificity of the amplicons.

### Phylogenetic analysis

*KRT1* sequence authenticity was verified by BLAST search in GenBank (Supplementary Table 6). Sequences were edited using MAFFT v 6.81b^52^ and Mesquite^53^. MRMODELTEST v.2.3^54^ was used to select the best-fit model of nucleotide substitution under the Akaike information criterion (AIC)^55^. Bayesian inference of phylogeny was performed using BEAST v 1.6.1^56^ with default settings except for GRT + I + G model. An uncorrelated relaxed clock fixed to lognormal distribution was employed as the site model using a Yule speciation tree prior sampled every 10,000th generation for 100 million generations. Effective sample seizes (ESS) were verified using Tracer v1.5 and a consensus tree was constructed in TreeAnnotator v1.6.1 with 20% burn-in. For the concatenated dataset, all parameters were estimated independently for each partition and displayed using FigTree v1.4.2. (https://www.geospiza.com/finchtv).

### Ethics approval and consent to participate

All animal procedures in the study were approved by the ethics committee for animal experiments at the Guangdong Institute of Applied Biological Resources (reference number G2ABR20170523) and followed basic principles. We confirm that all methods were performed in accordance with relevant guidelines and regulations.

## Supplementary information

Supplementary Information.

## Data Availability

Data analyzed in the current study are included within the article and its supplementary material. All unigene sequences from *A. albiventris* have been deposited in the GenBank Sequence Read Archive (SRA) under accession number PRJNA561241 for SUB6195278. We have uploaded supplemental material to figshare via the GSA Portal. Supplementary Table 1 contains the summary of sequencing statistics for the transcriptomes; Supplementary Table 2 contains the summary of primer information used in real-time PCR analysis; Supplementary Table 3 contains the DEGs between HS1Y and HS1N; Supplementary Table 4 contains the DEGs between HH1Y and HH1N; Supplementary Table 5 contains the DEGs between HH5 and HS5; Supplementary Table 6 contains the species name and NCBI serial numbers of 19 additional species for phylogenetic analysis.

## Abbreviations

*A. albiventris*: *Atelerix albiventris*
Hiseq: High-throughput sequencing
PCA: Principal component analysis
KRT: Keratin
FGF: Fibroblast growth factor
SHH: Sonic hedgehog
BMP: Bone morphogenetic protein
DEGs: Differentially expressed genes
RT-qPCR: Quantitative real time polymerase chain reaction
SOM: Self-organizing feature map

## References

[1] Gandolfi B (2010). The naked truth: Sphynx and Devon Rex cat breed mutations in KRT71. Mamm. Genome.

[2] Di-Poï N, Milinkovitch MC (2016). The anatomical placode in reptile scale morphogenesis indicates shared ancestry among skin appendages in amniotes. Sci. Adv..

[3] Guo J (2013). The Morphological Structure and Development of Hedgehog Quill.

[4] Millar SE (2002). Molecular mechanisms regulating hair follicle development. J. Invest. Dermatol..

[5] Paus R (1999). A comprehensive guide for the recognition and classification of distinct stages of hair follicle. J. Invest. Dermatol..

[6] Won HI (2018). De novo assembly of the burying beetle *Nicrophorus orbicollis* (Coleoptera: Silphidae) transcriptome across developmental stages with identification of key immune transcripts. J. Genomics.

[7] Noramly S, Freeman A, Morgan BA (1999). Beta-catenin signaling can initiate feather bud development. Development.

[8] Andl T, Reddy ST, Gaddapara T, Millar SE (2002). WNT signals are required for the initiation of hair follicle development. Dev. Cell.

[9] Tsai SY (2014). Wnt/β-catenin signaling in dermal condensates is required for hair follicle formation. Dev. Biol..

[10] Bitgood MJ, McMahon AP (1995). Hedgehog and Bmp genes are coexpressed at many diverse sites of cell–cell interaction in the mouse embryo. Dev. Biol..

[11] Iseki S (1996). Sonic hedgehog is expressed in epithelial cells during development of whisker, hair, and tooth. Biochem. Biophys. Res. Commun..

[12] Luetteke NC (1993). TGFα deficiency results in hair follicle and eye abnormalities in targeted and waved-1 mice. Cell.

[13] Yamamoto H (1998). Defective trophoblast function in mice with a targeted mutation of Ets2. Genes Dev..

[14] Godwin AR, Capecchi MR (1998). Hoxc13 mutant mice lack external hair. Genes Dev..

[15] Potter CS (2011). The nude mutant gene Foxn1 Is a HOXC13 regulatory target during hair follicle and nail differentiation. J. Invest. Dermatol..

[16] Cadieu E (2009). Coat variation in the domestic dog is governed by variants in three genes. Science.

[17] Koch PJ (2007). Mice expressing a mutant Krt75 (K6hf) allele develop hair and nail defects resembling pachyonychia congenita. J. Invest. Dermatol..

[18] Mou C, Jackson B, Overbeek PA, Schneider P, Headon DJ (2006). Generation of the primary hair follicle pattern. Proc. Natl. Acad. Sci..

[19] Pummila M (2006). Ectodysplasin has a dual role in ectodermal organogenesis: inhibition of Bmp activity and induction of Shh expression. Development.

[20] Kamberov YG (2013). Modeling recent human evolution in mice by expression of a selected EDAR variant. Cell.

[21] Duverger O, Morasso MI (2009). Epidermal patterning and induction of different hair types during mouse embryonic development. Birth Defects Res. C Embryo Today Rev..

[22] Hardy MH (1992). The secret life of the hair follicle. Trends Genet..

[23] Laurikkala J (2002). Regulation of hair follicle development by the TNF signal ectodysplasin and its receptor Edar. Development.

[24] Qiao L, Yang W, Fu J, Song Z (2013). Transcriptome profile of the green odorous frog (*Odorrana margaretae*). PLoS ONE.

[25] Shao Y (2015). Transcriptomes reveal the genetic mechanisms underlying ionic regulatory adaptations to salt in the crab-eating frog. Sci. Rep..

[26] Kim S, Wong P, Coulombe PA (2006). A keratin cytoskeletal protein regulates protein synthesis and epithelial cell growth. Nature.

[27] Owens P (2008). Smad4-dependent desmoglein-4 expression contributes to hair follicle integrity. Dev. Biol..

[28] Bu R, Li J, Liu KD, Liu JF, Liu N (2014). Study on the differential expression of the SFN gene in fine-wool sheep. Heilongjiang Anim. Sci. Vet. Med..

[29] Jung HS (1998). Local inhibitory action of BMPs and their relationships with activators in feather formation: Implications for periodic patterning. Dev. Biol..

[30] Hébert JM, Rosenquist T, Götz J, Martin GR (1994). FGF5 as a regulator of the hair growth cycle: Evidence from targeted and spontaneous mutations. Cell.

[31] Schweizer J (2006). New consensus nomenclature for mammalian keratins. J. Cell Biol..

[32] Plowman JE (2003). Proteomic database of wool components. J. Chromatogr. B Anal. Technol. Biomed. Life Sci..

[33] He J-M (2017). Analysis of genetic effects of KAP16 gene with main economic traits in fine wool sheep. Southwest China J. Agric. Sci..

[34] Wang, L. *Expression and Analysis of KAP7.1, KAP8.2 Gene in Liaoning New-Breed Cashmere Goat Hair Follicle*. Thesis (Liaoning Normal University, 2010). 10.1007/s11033-010-9968-6 20151326

[35] Akita M (2009). Characterization of hair follicles in Hirosaki hairless rats with deletion of basic hair keratin genes. Enlarged medulla, loss of cuticle and long catagen. Hirosaki Med. J..

[36] Connors JB, Rahil AK, Smith FJD, McLean WHI, Milstone LM (2001). Delayed-onset pachyonychia congenita associated with a novel mutation in the central 2B domain of keratin 16. Br. J. Dermatol..

[37] Smith FJD (1998). A mutation in human keratin K6b produces a phenocopy of the K17 disorder pachyonychia congenita type 2. Hum. Mol. Genet..

[38] Parsons YM, Cooper DW, Piper LR (1994). Evidence of linkage between high-glycine-tyrosine keratin gene loci and wool fibre diameter in a Merino half-sib family. Anim. Genet..

[39] Rogers MA (2001). Characterization of a cluster of human high/ultrahigh sulfur keratin-associated protein genes embedded in the type I keratin gene domain on chromosome 17q12-21. J. Biol. Chem..

[40] Khan I (2014). Mammalian keratin associated proteins (KRTAPs) subgenomes: disentangling hair diversity and adaptation to terrestrial and aquatic environments. BMC Genomics.

[41] Hoffmann FG, Opazo JC, Storz JF (2008). Rapid rates of lineage-specific gene duplication and deletion in the α-globin gene family. Mol. Biol. Evol..

[42] Opazo JC, Hoffmann FG, Storz JF (2008). Differential loss of embryonic globin genes during the radiation of placental mammals. Proc. Natl. Acad. Sci. U. S. A..

[43] Chamcheu JC (2011). Keratin gene mutations in disorders of human skin and its appendages. Arch. Biochem. Biophys..

[44] Toivola DM, Boor P, Alam C, Strnad P (2015). Keratins in health and disease. Curr. Opin. Cell Biol..

[45] Magin TM, Vijayaraj P, Leube RE (2007). Structural and regulatory functions of keratins. Exp. Cell Res..

[46] Choo SW (2016). Pangolin genomes and the evolution of mammalian scales and immunity. Genome Res..

[47] Manehisa M, Goto S (2000). KEGG: Kyoto encyclopedia of genes and genomes. Nucleic Acids Res..

[48] Kanehisa M, Sato Y, Furumichi M, Morishima K, Tanabe M (2019). New approach for understanding genome variations in KEGG. Nucleic Acids Res..

[49] Langmead B, Salzberg S, Langmead B, Salzberg SL (2012). Fast gapped-read alignment with Bowtie 2. Nat. Methods.

[50] Li B, Dewey CN (2011). RSEM: accurate transcript quantification from RNA-Seq data with or without a reference genome. Bioinformatics.

[51] Bustin, S. A., et al. MIQE précis: Practical implementation of minimum standard guidelines for fluorescence-based quantitative real-time PCR experiments. *BMC Mol. Biol.* **11**, 10.1186/1471-2199-11-74 (2010). 10.1186/1471-2199-11-74 PMC2955025 20858237

[52] Katoh K, Asimenos G, Toh H (2009). Multiple alignment of DNA sequneces with MAFFT. Methods Mol. Biol..

[53] Maddison WP (2009). Mesquite: a molecular system for evolutionary analysis. Evolution.

[54] Nylander JA (2004). MrModeltest v2. Program distributed by the author.

[55] Posada D, Crandall KA (1998). MODELTEST: testing the model of DNA substitution. Bioinformatics.

[56] Bouckaert R (2014). BEAST 2: a software platform for Bayesian evolutionary analysis. PLoS Comput. Biol..

