# Supplementary material for: Transcriptome analysis reveals the genetic basis underlying the development of skin appendages and immunity in hedgehog (Atelerix albiventris)
Source: Sci Rep. 2020 Aug 18;10:13920. doi: 10.1038/s41598-020-70844-y (PMC7435191; doi:10.1038/s41598-020-70844-y)
Supplement: Supplementary file 1 — Supplementary Information. [file 41598_2020_70844_MOESM1_ESM.pdf]

Transcriptome analysis reveals the genetic basis underlying the development of skin appendages and immunity in hedgehog (*Atelerix albiventris*)

Hui-Ming Li[1], Bi-Ze Yang[1], Xiu-Juan Zhang[1], Hai-Ying Jiang[1], Lin-Miao Li[1], Hafiz Ishfaq Ahmad[1], Jin-Ping Chen[1*]

[1]Guangdong Key Laboratory of Animal Conservation and Resource Utilization, Guangdong Public Laboratory of Wild Animal Conservation and Utilization, Guangdong Institute of Applied Biological Resources, Guangdong Academy of Science, Guangzhou 510260, China

*Corresponding author: chenjp@giabr.gd.cn

Supplementary table 1 Sample IDs for transcriptome sequencing

**Sample ID**	**RNA integrity number （RIN）**	**Concen. (ng/ul)**	**Raw Reads**	**Clean reads**	**Clean bases (G)**	**Error (%)**	**Q20 (%)**	**Q30 (%)**	**GC (%)**
HH1_N1	9.3	414	84746676	82076208	12.3	0.03	96.85	92.17	58.14
HH1_N2	7.6	252	91823824	89358204	13.4	0.03	97.2	93.01	58.02
HS1_N1	8.4	1455	87533090	84776044	12.72	0.03	96.44	91.23	58.13
HS1_N2	7.8	1986	80827066	78473126	11.77	0.03	95.92	90.09	57
HH1_Y1	8.6	324	78351662	76208670	11.43	0.03	97.01	92.49	57.67
HH1_Y2	8.4	416	83163428	80515340	12.08	0.03	97	92.5	58.74
HH1_Y3	8.9	674	91357512	88846572	13.33	0.03	97.3	93.23	58.61
HS1_Y1	8.9	1290	97436460	94259192	14.14	0.03	96.46	91.35	58.29
HS1_Y2	8.6	1350	92684804	89648842	13.45	0.03	96.4	91.22	58.46
HS1_Y3	6.6	90	86012194	84258058	12.64	0.03	97.05	92.27	49.4
HH5_1	8.3	134	81247180	78253286	11.74	0.03	97.3	93.26	59.02
HH5_2	9.5	42	92765874	90002040	13.5	0.03	96.84	92.14	54.7
HH5_3	7.8	338	87389764	84746764	12.71	0.03	97.1	92.81	58.38
HS5_1	8.8	204	100102332	97325590	14.6	0.03	96.42	91.29	58.04
HS5_2	7.1	204	98119576	95197288	14.28	0.03	96.36	91.15	56.88
HS5_3	6.4	78	87681000	84955022	12.74	0.03	96.35	91.06	55.88

Supplementary table 2 Summary of primers of real-time PCR assay

Gene ID	Symbol	Primer-F (5'-3')	Primer-R (3'-5')
Cluster-33503.52810	ARRB	AAAACAGAAAGGGAAACG	AGGAGGTGGGCGAAGA
XM_007527837.2	GAPDH	ACTCCACTCACGGCAAAT	GTACTCGGCACCAGCATC
Cluster-33503.134689	GRB2	GGGGTTTGATGCGGATAT	GCCACCGAAAGTGACGAG
Cluster-33503.28078	HOXC13	GTGACCCTGGAGCAAA	CTATCTATTAGTGGGACTTGG
Cluster-33503.122683	KRT1	AGCCGCAGTTGCCCACA	CCAGCACGATGCCTTACA
Cluster-33503.128985	LEF1	TTTCCGTAGTTGTCCCG	TGAATGCGTTCATGCTGT
Cluster-33503.47741	RSPO2	CCGGAGACCCTGGAGTTGT	GGACCGCCAGAGGCAATT
Cluster-33503.95190	SFN	CTCACTTCACAGAGCCTTTC	GCTGCTGCGAGACAACCT
Cluster-33503.30444	TCF7	CAAAGTGATTGCCGAGTG	TTGTCCCGTGCTGACC
Cluster-33503.107807	TCNNB1	CTTCTGGGCTACGATGAC	CAACTCTGCTTCCTGGTG
Cluster-33503.38152	TGFB2	GAACCCGACTGTGCTGA	TGCCTCCGTCCTCTTTA
Cluster-33503.47776	WIF1	AAGTTCGTCTGTAGCGTGAT	CCTTCTCCAATGTTCCCT

Supplementary table 3 DEGs between HS1Y and HS1N

Gene ID	Gene name	HS1Y readcount	HS1N readcount	log2FoldChange	pval	padj
Cluster-33503.93079	APOE	0.000	135.036	-9.5605	1.45E-12	7.61E-08
Cluster-33503.83755	COX3	0.000	1125.958	-25.647	3.11E-09	7.26E-05
Cluster-33503.78173	--	0.000	398.766	-24.306	1.96E-08	0.00037414
Cluster-33503.96104	RP-L11e, RPL11	0.000	289.635	-23.902	3.35E-08	0.00058611
Cluster-33503.43551	COX2	0.000	188.780	-23.303	7.31E-08	0.0011806
Cluster-33503.93070	APOE	0.000	30.378	-7.4075	2.73E-06	0.023932
Cluster-33503.40525	SQLE	4.297	90.360	-4.4067	4.09E-07	0.0053651
Cluster-33503.71791	APOE	5.005	1155.373	-7.8257	2.25E-32	2.36E-27
Cluster-33503.74871	APOE	6.712	2263.274	-8.3611	3.22E-39	6.77E-34
Cluster-33503.64008	MYLK	19.914	192.079	-3.2642	2.88E-07	0.0043206
Cluster-33503.84059	ACTG1	25.821	409.076	-3.9892	3.70E-12	1.56E-07
Cluster-33503.71896	SH3GL	30.451	0.000	20.935	2.06E-06	0.019828
Cluster-33503.160189	PIGR	43.129	0.000	7.8749	2.08E-06	0.019828
Cluster-33503.115414	LRRC14B	57.989	1.453	5.2885	2.29E-06	0.020932
Cluster-33503.125788	LOC103118587	63.244	0.000	21.71	8.46E-07	0.0098759
Cluster-33503.30824	ANGPTL8	71.482	514.249	-2.8474	5.04E-06	0.037789
Cluster-33503.128444	--	86.082	0.000	22.347	3.99E-07	0.0053651
Cluster-33503.69414	ACTG1	87.841	1973.584	-4.4903	7.56E-20	5.29E-15
Cluster-82032.0	HMX1	172.142	18.835	3.1884	9.18E-07	0.010153
Cluster-33503.121879	FCN	236.844	25.913	3.1921	7.28E-07	0.0089964
Cluster-33503.50680	--	336.639	21.814	3.9508	5.45E-10	1.64E-05
Cluster-33503.127585	LOC103122410	558.088	42.529	3.7159	2.27E-11	7.94E-07
Cluster-33503.107454	TAGLN	673.046	7002.950	-3.3788	2.45E-09	6.44E-05
Cluster-33503.89984	--	896.546	96.115	3.2208	4.10E-06	0.033124
Cluster-33503.107453	TAGLN	896.666	4977.640	-2.4726	3.93E-06	0.03305
Cluster-33503.86800	HBA	1356.214	9843.402	-2.8594	4.34E-06	0.033743
Cluster-33503.129745	KRATP13-1	1414.024	160.601	3.1376	7.34E-09	0.00015421
Cluster-33503.40595	CRABP1	2226.207	11872.357	-2.4149	1.77E-06	0.018625

Supplementary table 4 DEGs between HH1Y and HH1N

Gene ID	Gene name	HH1Y readcount	HH1N readcount	log2FoldChange	pval	padj
Cluster-33503.83755	COX3	0.000	1722.539	-26.309	1.21E-09	1.07E-05
Cluster-33503.103697	COX1	0.000	1325.376	-25.546	3.58E-09	2.85E-05
Cluster-33503.78173	--	0.000	795.226	-25.235	5.53E-09	3.82E-05
Cluster-33503.96104	RP-L11e, RPL11	0.000	259.666	-23.658	4.61E-08	0.00026318
Cluster-33503.95063	--	0.000	186.635	-23.214	8.19E-08	0.00037165
Cluster-33503.93079	APOE	0.683	64.059	-6.5507	8.67E-08	0.00038271
Cluster-33503.85379	COX2	0.702	99.629	-7.1757	1.90E-06	0.0054054
Cluster-33503.74871	APOE	0.721	1137.660	-10.697	1.26E-22	9.98E-18
Cluster-33503.114665	LOC102243107	1.027	111.800	-6.7686	7.14E-07	0.0024226
Cluster-33503.71791	APOE	2.450	710.557	-8.2083	1.22E-33	1.94E-28
Cluster-33503.76770	APOE	10.655	95.660	-3.1688	5.44E-07	0.0019647
Cluster-33503.35715	SLC6A19	16.404	124.390	-2.9238	1.21E-05	0.025556
Cluster-33503.84059	ACTG1	18.922	323.389	-4.0987	4.40E-17	1.75E-12
Cluster-33503.27714	CTH	25.628	180.362	-2.8089	1.57E-05	0.032501
Cluster-33503.71896	SH3GL	30.290	0.000	21.031	1.85E-06	0.0053558
Cluster-33503.27650	CHR1	31.011	0.000	21.061	1.79E-06	0.0053558
Cluster-24458.2	MYH	32.181	0.000	21.108	1.70E-06	0.0052886
Cluster-23971.0	LOC107862771	35.422	0.000	7.6936	1.78E-06	0.0053558
Cluster-33503.78577	PDLIM7	42.477	239.314	-2.4952	1.21E-05	0.025556
Cluster-27220.0	(LOC107862766	53.885	3.606	3.878	2.76E-06	0.0074259
Cluster-33503.125440	KRT2	55.892	0.000	21.854	7.16E-07	0.0024226
Cluster-33503.28873	CLRN2	62.274	1.382	5.4875	3.64E-06	0.0093249
Cluster-33503.110885	--	66.637	0.000	22.096	5.38E-07	0.0019647
Cluster-33503.48696	CFD	71.853	6.429	3.4766	1.35E-06	0.0042948
Cluster-33503.69414	ACTG1	82.547	1457.644	-4.1441	4.51E-22	2.39E-17
Cluster-33503.35295	FABP7	83.658	356.331	-2.092	2.22E-05	0.043584
Cluster-33503.33273	HIF3A	93.631	576.072	-2.6223	8.61E-06	0.020121
Cluster-50926.0	LOC107860006	96.819	0.902	6.71	1.04E-08	6.86E-05
Cluster-33503.50604	CFD	99.929	8.349	3.5813	6.44E-08	0.00031024
Cluster-33503.82604	ALDOA	106.464	17.120	2.6336	2.53E-06	0.0069223
Cluster-33503.142390	AOX	109.915	14.180	2.9497	1.15E-05	0.025083
Cluster-33503.45503	CFD	111.498	6.967	4.001	3.33E-09	2.79E-05
Cluster-33503.63866	USMG5, DAPIT	114.101	11.347	3.3365	1.05E-07	0.00045271
Cluster-33503.46837	CFD	116.902	15.738	2.8927	1.83E-06	0.0053558
Cluster-33503.83099	--	118.143	17.864	2.7299	3.00E-06	0.0079505
Cluster-33503.115414	LRRC14B	120.413	6.575	4.202	5.87E-10	5.49E-06
Cluster-33503.93776	NPPA	163.972	12.190	3.7533	8.46E-12	1.22E-07
Cluster-33503.99455	CKM	168.166	29.614	2.5049	3.03E-07	0.0012029
Cluster-33503.99443	CKM	203.106	30.358	2.7441	1.26E-07	0.00052598
Cluster-33503.50610	CFD	206.593	18.139	3.5106	5.67E-10	5.49E-06

Cluster-33503.67947	SLIT3	245.385	46.929	2.388	3.29E-07	0.0012764
Cluster-33503.146366	MASP1	253.823	64.334	1.9797	2.11E-05	0.042496
Cluster-33503.50611	CFD	272.985	18.531	3.88	2.69E-13	7.12E-09
Cluster-33503.75382	chaC	317.223	939.250	-1.5656	9.69E-06	0.021966
Cluster-33503.27620	RBP4	317.285	91.225	1.7969	9.81E-06	0.021966
Cluster-33503.101049	--	326.998	103.639	1.6574	1.72E-05	0.035084
Cluster-33503.74547	LRRC30	343.894	71.064	2.2767	6.33E-06	0.015721
Cluster-33503.38566	UCP2_3	350.275	49.498	2.8215	2.98E-10	3.16E-06
Cluster-33503.82343	--	364.933	95.154	1.9382	7.03E-07	0.0024226
Cluster-33503.121879	FCN	369.511	55.201	2.7422	1.25E-12	2.49E-08
Cluster-33503.100489	TCAP	380.445	129.951	1.5494	2.36E-05	0.045819
Cluster-33503.52438	GPNMB	396.661	22.753	4.1295	1.07E-12	2.43E-08
Cluster-33503.50680	--	468.965	106.098	2.1445	2.40E-08	0.00015286
Cluster-33503.50612	CFD	491.173	53.877	3.1887	2.24E-10	2.54E-06
Cluster-33503.30717	SERPINB1	531.953	139.844	1.9288	1.10E-05	0.024209
Cluster-33503.29591	ART3_5	535.599	103.148	2.3771	7.70E-06	0.018372
Cluster-33503.47433	SERPINB	547.822	139.385	1.9753	5.97E-08	0.00030247
Cluster-33503.46861	SERPINB1	662.681	216.950	1.6107	2.16E-05	0.043
Cluster-33503.65963	FCN	728.915	183.418	1.9904	5.16E-08	0.00028299
Cluster-33503.127585	LOC103122410	758.901	171.950	2.1422	6.02E-08	0.00030247
Cluster-33503.99442	E2.7.3.2	820.536	159.326	2.3635	1.25E-06	0.0040544
Cluster-33503.50605	CFD	946.809	102.246	3.2117	2.29E-11	2.80E-07
Cluster-33503.40311	GPX6	1055.103	7501.224	-2.8298	1.35E-11	1.79E-07
Cluster-33503.29548	PAMR1	1092.506	300.874	1.8604	5.04E-07	0.0019061
Cluster-33503.50603	CFD	1118.064	127.726	3.13	2.65E-12	4.22E-08
Cluster-33503.99447	CKM	1160.856	226.938	2.3547	5.41E-09	3.82E-05
Cluster-33503.122220	FCN	1220.254	386.136	1.6596	7.75E-06	0.018372
Cluster-33503.67786	FCN	1446.210	388.383	1.8967	6.09E-08	0.00030247
Cluster-33503.142217	NUP205, NUP192	1510.353	362.757	2.0577	4.61E-09	3.49E-05
Cluster-33503.100134	FABP3	1589.090	453.135	1.8099	9.05E-06	0.020841
Cluster-33503.93217	SLPI	1591.578	493.620	1.6888	6.43E-06	0.015728
Cluster-33503.54524	TF	1651.530	329.244	2.3271	3.89E-08	0.0002379
Cluster-33503.98980	FCN	1736.197	508.772	1.7707	7.04E-08	0.00032883
Cluster-33503.93054	APOE	1952.662	676.060	1.5302	2.47E-06	0.0068841
Cluster-33503.99016	FCN	2135.446	629.724	1.7615	8.88E-07	0.0029411
Cluster-33503.50332	CIDEC	2184.052	712.572	1.616	1.24E-05	0.025979
Cluster-33503.50609	CFD	2496.305	238.179	3.3895	7.60E-14	2.41E-09
Cluster-33503.99017	FCN	3018.019	840.049	1.8451	4.64E-08	0.00026318
Cluster-33503.99433	CKM	3678.842	1030.144	1.8364	4.84E-06	0.012221
Cluster-33503.46860	MUSTN1	3761.834	1229.407	1.6134	3.36E-06	0.0087486
Cluster-33503.72876	FCN	6280.950	1685.051	1.8982	1.87E-07	0.00076126
Cluster-33503.98985	FCN	58233.126	10642.132	2.452	2.11E-12	3.73E-08

Supplementary table 5 DEGs between HH5 and HS5

Gene ID	KO Name	HH5 readcount	HS5 readcount	log2FoldChange	pval	padj	NT Description
Cluster-33503.37077	ZIC4	5.779	308.149	-5.7335	9.28E-22	6.00E-18	Macaca fascicularis complete genome, chromosome chr2
Cluster-33503.5792	ZIC4	0.000	76.701	-8.7764	4.08E-10	2.96E-07	PREDICTED: Erinaceus europaeus Zic family member 4 (ZIC4), transcript variant X1, mRNA
Cluster-33503.36616	ZIC1	4.419	300.859	-6.2146	8.06E-20	4.13E-16	PREDICTED: Erinaceus europaeus Zic family member 1 (ZIC1), mRNA
Cluster-33503.129343	WNT3	105.876	463.166	-2.13	0.00010649	0.022074	PREDICTED: Ovis aries musimon wingless-type MMTV integration site family member 3 (WNT3), transcript variant X2, mRNA
Cluster-33503.183148	WNT10	186.733	33.339	2.4899	9.48E-05	0.020011	PREDICTED: Erinaceus europaeus Wnt family member 10B (WNT10B), mRNA
Cluster-33503.47776	WIF1	2471.179	535.001	2.208	3.85E-05	0.0091392	PREDICTED: Erinaceus europaeus WNT inhibitory factor 1 (WIF1), mRNA
Cluster-33503.165150	VAX	10.056	126.521	-3.6921	5.39E-10	3.77E-07	Homo sapiens engrailed homeobox 1 (EN1), RefSeqGene on chromosome 2
Cluster-33503.29616	UBC	0.000	25.302	-7.1776	2.24E-06	0.00071988	PREDICTED: Gossypium arboreum polyubiquitin (LOC108453253), mRNA
Cluster-33503.181682	TYRP1	1.199	169.712	-7.4414	1.94E-13	3.28E-10	PREDICTED: Erinaceus europaeus tyrosinase-related protein 1 (TYRP1), mRNA
Cluster-24090.0	TUBA	0.000	46.841	-8.063	1.02E-08	5.37E-06	Gossypium hirsutum tubulin alpha-4 chain (LOC107905189), mRNA >gb|FJ594490.1| Gossypium hirsutum alpha-tubulin 10 mRNA, complete cds

Cluster-71241.0	TUBA	0.000	23.956	-7.0939	4.40E-06	0.0013176	PREDICTED: Gossypium arboreum tubulin alpha-4 chain-like (LOC108475898), mRNA
Cluster-33503.150625	TRPV3	177.314	1091.230	-2.6184	1.24E-08	6.36E-06	PREDICTED: Erinaceus europaeus transient receptor potential cation channel subfamily V member 3 (TRPV3), mRNA
Cluster-33503.21249	TRPV3	116.409	649.872	-2.4759	7.93E-08	3.44E-05	PREDICTED: Erinaceus europaeus transient receptor potential cation channel subfamily V member 3 (TRPV3), mRNA
Cluster-33503.81264	TPM2	40.544	495.896	-3.6277	2.95E-09	1.75E-06	PREDICTED: Erinaceus europaeus tropomyosin 2 (beta) (TPM2), transcript variant X4, mRNA
Cluster-33503.81267	TPM2	32.586	488.748	-3.9269	4.15E-09	2.37E-06	PREDICTED: Erinaceus europaeus tropomyosin 2 (beta) (TPM2), transcript variant X4, mRNA
Cluster-33503.177400	TPM2	3.057	75.760	-4.6983	8.00E-08	3.46E-05	PREDICTED: Erinaceus europaeus tropomyosin 2 (beta) (TPM2), transcript variant X4, mRNA
Cluster-33503.103239	TPM2	12.215	154.361	-3.707	1.30E-07	5.36E-05	--
Cluster-33503.74157	TPM2	5.338	52.639	-3.3384	1.79E-05	0.0046019	PREDICTED: Erinaceus europaeus tropomyosin 2 (beta) (TPM2), transcript variant X3, mRNA
Cluster-33503.78625	TPM1	38.168	747.780	-4.3108	2.39E-18	9.68E-15	PREDICTED: Erinaceus europaeus tropomyosin 1 (alpha) (TPM1), transcript variant X7, mRNA
Cluster-33503.77913	TPM1	103.382	1773.061	-4.1077	1.39E-16	4.49E-13	PREDICTED: Erinaceus europaeus tropomyosin 1 (alpha) (TPM1), transcript variant X7, mRNA
Cluster-33503.77755	TPM1	6.316	108.477	-4.1871	1.74E-08	8.63E-06	PREDICTED: Erinaceus europaeus tropomyosin 1 (alpha) (TPM1), transcript variant X7, mRNA
Cluster-33503.78635	TPM1	9.738	144.931	-3.9548	3.65E-08	1.72E-05	PREDICTED: Erinaceus europaeus tropomyosin 1 (alpha) (TPM1), transcript variant X7, mRNA
Cluster-33503.78633	TPM1	5.962	95.858	-4.0614	4.48E-08	2.07E-05	PREDICTED: Erinaceus europaeus tropomyosin 1 (alpha) (TPM1), transcript variant X7, mRNA

Cluster-33503.78622	TPM1	1.959	51.695	-4.9163	3.06E-07	0.00011635	PREDICTED: Erinaceus europaeus tropomyosin 1 (alpha) (TPM1), transcript variant X7, mRNA
Cluster-33503.36473	TNS	275.872	1061.541	-1.9438	5.45E-06	0.0015904	PREDICTED: Erinaceus europaeus tensin 1 (TNS1), transcript variant X6, mRNA
Cluster-33503.36470	TNS	638.621	2415.750	-1.9192	8.23E-06	0.002296	PREDICTED: Erinaceus europaeus tensin 1 (TNS1), transcript variant X6, mRNA
Cluster-33503.36474	TNS	190.983	753.206	-1.98	2.19E-05	0.0054947	PREDICTED: Erinaceus europaeus tensin 1 (TNS1), transcript variant X6, mRNA
Cluster-33503.36471	TNS	234.095	909.154	-1.9539	0.00014292	0.028347	PREDICTED: Erinaceus europaeus tensin 1 (TNS1), transcript variant X6, mRNA
Cluster-33503.36469	TNS	33.000	146.141	-2.1295	0.00020183	0.038138	PREDICTED: Erinaceus europaeus tensin 1 (TNS1), transcript variant X6, mRNA
Cluster-33503.25346	TNFRSF7, CD27	42.124	1.279	4.9978	9.52E-05	0.02007	PREDICTED: Erinaceus europaeus CD27 molecule (CD27), mRNA
Cluster-33503.133986	TGM6	196.932	1657.425	-3.0715	1.20E-08	6.18E-06	PREDICTED: Erinaceus europaeus transglutaminase 6 (TGM6), mRNA
Cluster-33503.35131	TGM6	71.854	358.227	-2.3103	0.00013858	0.027567	PREDICTED: Erinaceus europaeus transglutaminase 6 (TGM6), mRNA
Cluster-33503.108722	TGM3	1036.475	4995.244	-2.2693	1.01E-07	4.28E-05	PREDICTED: Erinaceus europaeus transglutaminase 3 (TGM3), mRNA
Cluster-33503.112838	TGM3	2.080	47.519	-4.3902	1.43E-06	0.0004806	PREDICTED: Erinaceus europaeus transglutaminase 3 (TGM3), mRNA
Cluster-33503.39189	TBX5, HOS	116.511	2.964	5.3585	1.81E-10	1.48E-07	PREDICTED: Erinaceus europaeus T-box 5 (TBX5), mRNA
Cluster-33503.86068	TBX3	801.039	244.187	1.7148	0.00010179	0.021262	PREDICTED: Erinaceus europaeus T-box 3 (TBX3), transcript variant X1, mRNA
Cluster-33503.117305	STAT4	1.020	26.645	-4.5424	5.86E-05	0.01317	PREDICTED: Erinaceus europaeus signal transducer and activator of transcription 4 (STAT4), mRNA

Cluster-33503.130257	STAB2, HARE	232.001	33.031	2.8126	0.00015172	0.029918	PREDICTED: Erinaceus europaeus stabilin 2 (STAB2), mRNA
Cluster-33503.85491	SPP1, BNSP, OPN	854.899	4822.446	-2.496	0.00015582	0.030484	PREDICTED: Erinaceus europaeus secreted phosphoprotein 1 (SPP1), mRNA
Cluster-33503.35675	SPINT1	958.767	2983.027	-1.6374	0.00010577	0.021941	PREDICTED: Erinaceus europaeus serine peptidase inhibitor, Kunitz type 1 (SPINT1), transcript variant X1, mRNA
Cluster-33503.36292	SPEG	150.934	784.548	-2.3788	2.59E-06	0.00081866	PREDICTED: Erinaceus europaeus striated muscle-specific serine/threonine-protein kinase-like (LOC107522988), mRNA
Cluster-33503.135371	SPEG	187.378	868.588	-2.2124	3.07E-06	0.00095303	PREDICTED: Erinaceus europaeus striated muscle preferentially expressed protein kinase (LOC103121502), transcript variant X1, mRNA
Cluster-4286.0	speD, AMD1	0.000	31.219	-7.4807	1.32E-06	0.00044733	PREDICTED: Gossypium arboreum S-adenosylmethionine decarboxylase proenzyme-like (LOC108465128), mRNA
Cluster-33503.136941	SNX20	60.305	9.230	2.7234	0.00010419	0.021664	PREDICTED: Erinaceus europaeus sorting nexin 20 (SNX20), mRNA
Cluster-33503.37413	SLCO5A	59.728	310.304	-2.3718	3.16E-05	0.0076234	PREDICTED: Erinaceus europaeus solute carrier organic anion transporter family member 5A1 (SLCO5A1), transcript variant X1, mRNA
Cluster-33503.21686	SLC7A11	83.524	634.221	-2.9208	2.80E-11	2.74E-08	PREDICTED: Erinaceus europaeus solute carrier family 7 member 11 (SLC7A11), mRNA
Cluster-33503.2265	SLC5A10, SGLT5	9.717	84.871	-3.168	2.35E-06	0.00075044	PREDICTED: Erinaceus europaeus solute carrier family 5 member 10 (SLC5A10), transcript variant X2, mRNA
Cluster-33503.169197	SLC42A, RHAG,	121.579	6588.525	-5.7627	9.03E-23	8.17E-19	PREDICTED: Erinaceus europaeus Rh family C glycoprotein (RHCG), transcript variant X1, mRNA

	RHBG, RHCG						
Cluster-33503.136672	SLC20A, PIT	480.696	1639.261	-1.7702	4.92E-05	0.011336	PREDICTED: Erinaceus europaeus solute carrier family 20 member 2 (SLC20A2), mRNA
Cluster-33503.45763	SLC10A2, ASBT	2.578	52.346	-4.4744	8.37E-07	0.00029238	PREDICTED: Erinaceus europaeus solute carrier family 10 member 2 (SLC10A2), mRNA
Cluster-33503.146529	SHROOM	432.490	1566.512	-1.8566	1.25E-05	0.0033761	PREDICTED: Erinaceus europaeus shroom family member 3 (SHROOM3), mRNA
Cluster-33503.28461	SHH	1003.330	152.174	2.7189	5.31E-05	0.012058	PREDICTED: Erinaceus europaeus sonic hedgehog (SHH), mRNA
Cluster-33503.135278	SFRP4	523.331	148.584	1.8158	0.00010398	0.021637	PREDICTED: Erinaceus europaeus secreted frizzled related protein 4 (SFRP4), mRNA
Cluster-33503.95188	SFN	3406.447	15040.301	-2.1427	2.52E-07	9.75E-05	PREDICTED: Erinaceus europaeus stratifin (SFN), mRNA
Cluster-33503.95190	SFN	4371.564	21593.306	-2.3045	2.67E-07	0.00010223	PREDICTED: Erinaceus europaeus stratifin (SFN), mRNA
Cluster-33503.67818	SFN	2066.952	10690.202	-2.3711	6.05E-07	0.00021657	PREDICTED: Erinaceus europaeus stratifin (SFN), mRNA
Cluster-33503.106990	SFN	914.026	4469.236	-2.2906	6.68E-07	0.00023724	PREDICTED: Erinaceus europaeus stratifin (SFN), mRNA
Cluster-33503.103241	SFN	334.849	1479.978	-2.146	1.19E-06	0.00040528	PREDICTED: Erinaceus europaeus stratifin (SFN), mRNA
Cluster-33503.95187	SFN	156.324	779.102	-2.322	1.32E-06	0.00044733	PREDICTED: Erinaceus europaeus stratifin (SFN), mRNA
Cluster-33503.67821	SFN	53.773	249.863	-2.2245	2.06E-06	0.00066563	PREDICTED: Erinaceus europaeus stratifin (SFN), mRNA
Cluster-33503.53756	SFN	37.470	176.381	-2.2405	4.11E-06	0.0012425	PREDICTED: Erinaceus europaeus stratifin (SFN), mRNA

Cluster-33503.106810	SERPINE2	1412.907	5606.239	-1.9887	9.47E-05	0.020011	PREDICTED: Erinaceus europaeus serpin family E member 2 (SERPINE2), transcript variant X2, mRNA
Cluster-33503.192177	SERPIND1, HCF2	12.963	81.933	-2.6396	0.00015252	0.030011	PREDICTED: Erinaceus europaeus serpin family D member 1 (SERPIND1), mRNA
Cluster-33503.177790	SERPINB11_12	7.436	563.866	-6.2382	9.61E-17	3.22E-13	PREDICTED: Erinaceus europaeus serpin family B member 11 (SERPINB11), mRNA
Cluster-33503.27879	SERPINB11_12	74.255	3428.571	-5.5344	1.78E-16	5.62E-13	PREDICTED: Erinaceus europaeus serpin family B member 11 (SERPINB11), mRNA
Cluster-33503.38330	SERPINB	49.185	1260.263	-4.6697	2.33E-10	1.82E-07	PREDICTED: Erinaceus europaeus serpin B3-like (LOC103114456), mRNA
Cluster-33503.164762	SERPINB	73.497	1027.316	-3.8091	3.98E-09	2.29E-06	PREDICTED: Erinaceus europaeus serpin B13 (LOC103114269), transcript variant X1, mRNA
Cluster-33503.164761	SERPINB	231.256	3782.107	-4.0333	2.48E-08	1.19E-05	PREDICTED: Erinaceus europaeus serpin B13 (LOC103114269), transcript variant X1, mRNA
Cluster-5997.0	SERPINA1, AAT	0.000	16.812	-6.6019	0.0001995	0.037803	Mus musculus serine (or cysteine) preptidase inhibitor, clade A, member 1B (Serpina1b), mRNA
Cluster-33503.68110	SELL	160.743	32.570	2.3089	1.69E-05	0.0043824	PREDICTED: Erinaceus europaeus selectin L (SELL), transcript variant X1, mRNA
Cluster-4305.0	RP-L3e, RPL3	0.000	12.688	-6.177	0.00027593	0.049489	PREDICTED: Gossypium arboreum 60S ribosomal protein L3-like (LOC108453632), mRNA
Cluster-33503.5273	RH	28.290	272.985	-3.2689	2.71E-06	0.00085264	PREDICTED: Erinaceus europaeus Rh blood group CcEe antigens (RHCE), mRNA
Cluster-73088.0	REN	0.880	43.738	-5.332	4.56E-06	0.001359	PREDICTED: Erinaceus europaeus renin (REN), mRNA
Cluster-33503.128115	RECK, ST15	931.621	293.753	1.6639	0.00014359	0.028439	PREDICTED: Orcinus orca reversion-inducing-cysteine-rich protein with kazal motifs (RECK), transcript variant X1, mRNA

Cluster-54788.0	RCHY1, PIRH2	16.454	0.000	6.4397	4.56E-05	0.010644	PREDICTED: Capsicum annuum E3 ubiquitin-protein ligase MIEL1 (LOC107839088), transcript variant X3, mRNA
Cluster-59230.0	RBM3	0.000	27.946	-7.3195	1.02E-06	0.00035057	PREDICTED: Gossypium hirsutum glycine-rich RNA-binding protein GRP1A-like (LOC107942897), mRNA
Cluster-33503.27164	RASL12, RIS	413.515	2003.283	-2.2773	3.80E-06	0.0011621	PREDICTED: Erinaceus europaeus RAS like family 12 (RASL12), transcript variant X2, mRNA
Cluster-33503.27163	RASL12, RIS	95.692	449.304	-2.2343	4.85E-06	0.0014389	PREDICTED: Erinaceus europaeus RAS like family 12 (RASL12), transcript variant X1, mRNA
Cluster-33503.147595	RAPGEF3, EPAC1	107.869	363.552	-1.7492	0.00025609	0.046545	PREDICTED: Erinaceus europaeus Rap guanine nucleotide exchange factor 3 (RAPGEF3), mRNA
Cluster-33503.16470	RAP1GAP2	361.545	1088.553	-1.5904	6.03E-05	0.013476	PREDICTED: Erinaceus europaeus RAP1 GTPase activating protein 2 (RAP1GAP2), transcript variant X2, mRNA
Cluster-33503.34070	PTCH2	1486.021	369.442	2.0067	7.54E-05	0.016398	PREDICTED: Erinaceus europaeus patched 2 (PTCH2), transcript variant X1, mRNA
Cluster-33503.31290	PPP1R1B, DARPP32	544.614	44.963	3.5983	5.47E-09	3.03E-06	PREDICTED: Erinaceus europaeus protein phosphatase 1 regulatory inhibitor subunit 1B (PPP1R1B), mRNA
Cluster-33503.59519	PPP1R15A, GADD34	1389.383	6889.254	-2.31	1.78E-05	0.0045781	PREDICTED: Erinaceus europaeus protein phosphatase 1 regulatory subunit 15A (PPP1R15A), mRNA
Cluster-33503.32047	PPP1R14A, CPI17	188.683	1329.855	-2.8188	5.22E-07	0.00018837	PREDICTED: Erinaceus europaeus protein phosphatase 1 regulatory inhibitor subunit 14A (PPP1R14A), transcript variant X1, mRNA
Cluster-33503.32051	PPP1R14A, CPI17	14.499	128.079	-3.1572	2.36E-06	0.00075223	PREDICTED: Erinaceus europaeus protein phosphatase 1 regulatory inhibitor subunit 14A (PPP1R14A), transcript variant X1, mRNA

Cluster-4912.0	PPIF	0.000	28.254	-7.335	1.51E-06	0.00050426	PREDICTED: Gossypium hirsutum peptidyl-prolyl cis-trans isomerase (LOC107922329), mRNA
Cluster-33503.56414	PPIF	634.887	3468.263	-2.4496	4.99E-05	0.011464	PREDICTED: Erinaceus europaeus peptidylprolyl isomerase F (PPIF), mRNA
Cluster-33503.7007	POU3F, OTF	0.440	42.624	-6.2064	1.44E-06	0.00048292	PREDICTED: Erinaceus europaeus POU class 3 homeobox 3 (POU3F3), mRNA
Cluster-33503.132884	PLK3, CNK	382.886	1875.231	-2.2918	7.68E-06	0.0021694	PREDICTED: Erinaceus europaeus polo like kinase 3 (PLK3), mRNA
Cluster-33503.42804	PLA2G, SPLA2	300.653	82.705	1.8606	0.00016068	0.031277	PREDICTED: Erinaceus europaeus phospholipase A2 group IIF (PLA2G2F), mRNA
Cluster-33503.22526	PLA2G, SPLA2	315.809	1142.306	-1.8551	0.00018321	0.035109	PREDICTED: Erinaceus europaeus phospholipase A2 group IIE (PLA2G2E), mRNA
Cluster-33503.157931	PKP2	256.026	904.740	-1.8237	0.00010768	0.022253	PREDICTED: Erinaceus europaeus plakophilin 2 (PKP2), mRNA
Cluster-33503.46553	PKP1	463.274	1675.199	-1.855	7.79E-05	0.016915	PREDICTED: Erinaceus europaeus plakophilin 1 (PKP1), mRNA
Cluster-33503.46543	PKP1	278.911	1063.432	-1.9321	8.38E-05	0.01802	PREDICTED: Erinaceus europaeus plakophilin 1 (PKP1), mRNA
Cluster-33503.46535	PKP1	70.340	251.690	-1.8373	0.00020658	0.038874	PREDICTED: Erinaceus europaeus plakophilin 1 (PKP1), mRNA
Cluster-33503.46536	PKP1	70.340	251.690	-1.8373	0.00020658	0.038874	PREDICTED: Erinaceus europaeus plakophilin 1 (PKP1), mRNA
Cluster-33503.46540	PKP1	1778.422	5345.287	-1.5877	0.0002207	0.041019	PREDICTED: Erinaceus europaeus plakophilin 1 (PKP1), mRNA
Cluster-33503.21260	PIK3AP1, BCAP	248.560	76.123	1.7071	0.00022589	0.041812	PREDICTED: Erinaceus europaeus phosphoinositide-3-kinase adaptor protein 1 (PIK3AP1), partial mRNA

Cluster-33503.160189	PIGR	62.913	5.847	3.4664	0.00013446	0.026826	PREDICTED: Erinaceus europaeus polymeric immunoglobulin receptor (PIGR), transcript variant X1, mRNA
Cluster-6145.0	PGK, pgk	0.000	16.157	-6.5287	4.51E-05	0.010535	PREDICTED: Gossypium hirsutum phosphoglycerate kinase, cytosolic (LOC107922096), transcript variant X1, mRNA
Cluster-27719.0	petF	19.728	0.000	6.6987	2.20E-05	0.005496	PREDICTED: Capsicum annuum ferredoxin, chloroplastic (LOC107839912), mRNA
Cluster-33503.117122	PARV	59.751	10.774	2.4673	8.33E-05	0.017929	PREDICTED: Erinaceus europaeus parvin gamma (PARVG), mRNA
Cluster-33503.118714	PAK6	558.637	1880.593	-1.7522	2.59E-05	0.0063744	PREDICTED: Erinaceus europaeus p21 protein (Cdc42/Rac)-activated kinase 6 (PAK6), mRNA
Cluster-33503.111338	PAK6	235.167	704.872	-1.5848	0.00013701	0.027295	PREDICTED: Erinaceus europaeus p21 protein (Cdc42/Rac)-activated kinase 6 (PAK6), mRNA
Cluster-106769.0	PABPC	18.353	0.000	6.6063	2.26E-05	0.0056203	PREDICTED: Capsicum annuum polyadenylate-binding protein 8-like (LOC107840253), transcript variant X1, mRNA
Cluster-33503.129197	OVOL	73.555	472.358	-2.6915	8.41E-07	0.00029343	PREDICTED: Erinaceus europaeus ovo like zinc finger 1 (OVOL1), mRNA
Cluster-33503.129204	OVOL	15.255	110.234	-2.8927	1.48E-05	0.0039005	PREDICTED: Erinaceus europaeus ovo like zinc finger 1 (OVOL1), mRNA
Cluster-33503.129194	OVOL	24.423	145.924	-2.5959	3.98E-05	0.009408	PREDICTED: Erinaceus europaeus ovo like zinc finger 1 (OVOL1), mRNA
Cluster-33503.129192	OVOL	324.869	1110.808	-1.7735	0.00015716	0.030636	PREDICTED: Erinaceus europaeus ovo like zinc finger 1 (OVOL1), mRNA
Cluster-33503.21887	OTX1	87.246	9.569	3.2144	3.68E-07	0.00013753	PREDICTED: Erinaceus europaeus orthodenticle homeobox 1 (OTX1), mRNA

Cluster-33503.178776	OTUB2	555.539	2122.261	-1.9335	5.51E-06	0.0016064	PREDICTED: Erinaceus europaeus OTU deubiquitinase, ubiquitin aldehyde binding 2 (OTUB2), mRNA
Cluster-9747.0	OLFR	0.000	24.130	-7.1128	1.38E-06	0.00046535	PREDICTED: Erinaceus europaeus olfactory receptor 10J5-like (LOC103120178), mRNA
Cluster-33503.186690	OLFR	0.400	21.681	-5.983	8.49E-05	0.018172	PREDICTED: Erinaceus europaeus olfactory receptor 1044-like (LOC103117673), mRNA
Cluster-33503.44003	OASL	174.101	46.076	1.9216	7.01E-05	0.015441	PREDICTED: Erinaceus europaeus 2'-5'-oligoadenylate synthase-like protein 1 (LOC103115505), mRNA
Cluster-33503.127570	NTS	219.951	26.164	3.076	1.85E-05	0.0047355	PREDICTED: Erinaceus europaeus neurotensin (NTS), mRNA
Cluster-33503.38388	NNMT	215.441	21.574	3.3302	3.37E-07	0.00012671	PREDICTED: Erinaceus europaeus nicotinamide N-methyltransferase (NNMT), mRNA
Cluster-3459.1	ND4	0.000	20.335	-6.8718	2.15E-05	0.0054028	Mus musculus mitochondrial DNA, complete sequence, clone: B82-2748-95
Cluster-33503.182086	ND2	0.000	15.607	-6.4943	0.00027429	0.04926	Mus musculus mitochondrial DNA, complete sequence, clone: B82-2748-95
Cluster-33503.187998	ND1	0.000	19.365	-6.7896	8.98E-05	0.019061	Psoroptes cuniculi mitochondrion, complete genome
Cluster-33503.109305	NCR2	136.576	19.565	2.8127	7.74E-06	0.002183	PREDICTED: Erinaceus europaeus triggering receptor expressed on myeloid cells like 4 (TREML4), transcript variant X1, mRNA
Cluster-105170.0	NAPSA	69.633	5.687	3.6291	7.49E-05	0.01631	PREDICTED: Erinaceus europaeus napsin-A-like (LOC103120847), mRNA
Cluster-33503.125563	MYO18	493.856	2049.739	-2.0518	2.76E-05	0.0067449	PREDICTED: Erinaceus europaeus myosin XVIIIB (MYO18B), mRNA
Cluster-33503.41416	MYO18	105.038	416.453	-1.9795	0.00017069	0.032965	PREDICTED: Erinaceus europaeus myosin XVIIIB (MYO18B), mRNA

Cluster-33503.64009	MYLK	953.195	8206.299	-3.1064	9.50E-12	1.09E-08	PREDICTED: Erinaceus europaeus myosin light chain kinase (MYLK), transcript variant X2, mRNA
Cluster-33503.111513	MYLK	314.532	2200.077	-2.8062	9.81E-12	1.11E-08	PREDICTED: Erinaceus europaeus myosin light chain kinase (MYLK), transcript variant X1, mRNA
Cluster-33503.64010	MYLK	21.883	142.539	-2.7129	1.50E-06	0.00050192	PREDICTED: Erinaceus europaeus myosin light chain kinase (MYLK), transcript variant X2, mRNA
Cluster-33503.79610	MYL9	275.516	1344.737	-2.2898	1.98E-06	0.00064093	PREDICTED: Erinaceus europaeus myosin light chain 9 (MYL9), mRNA
Cluster-33503.96346	MYL9	188.918	887.295	-2.2356	5.28E-05	0.012015	PREDICTED: Erinaceus europaeus myosin light chain 9 (MYL9), mRNA
Cluster-33503.112316	MYL9	277.915	1265.849	-2.1903	7.37E-05	0.016108	PREDICTED: Erinaceus europaeus myosin light chain 9 (MYL9), mRNA
Cluster-33503.97766	MYL9	19.456	100.110	-2.3919	0.00014304	0.028351	PREDICTED: Erinaceus europaeus myosin light chain 9 (MYL9), mRNA
Cluster-33503.119126	MYH	457.752	8561.581	-4.2253	1.71E-22	1.32E-18	PREDICTED: Erinaceus europaeus myosin, heavy chain 11, smooth muscle (MYH11), transcript variant X2, mRNA
Cluster-33503.119123	MYH	211.926	3687.105	-4.1192	3.48E-22	2.42E-18	PREDICTED: Erinaceus europaeus myosin, heavy chain 11, smooth muscle (MYH11), transcript variant X2, mRNA
Cluster-33503.73398	MYH	40.471	746.877	-4.1878	1.08E-15	2.96E-12	PREDICTED: Erinaceus europaeus myosin, heavy chain 11, smooth muscle (MYH11), transcript variant X2, mRNA
Cluster-33503.125466	MYH	29.661	487.507	-4.0261	1.27E-15	3.38E-12	PREDICTED: Erinaceus europaeus myosin, heavy chain 11, smooth muscle (MYH11), transcript variant X2, mRNA

Cluster-33503.132056	MYH	1.279	33.624	-4.562	7.78E-06	0.0021882	PREDICTED: Erinaceus europaeus myosin, heavy chain 11, smooth muscle (MYH11), transcript variant X2, mRNA
Cluster-33503.49666	MYH	1134.398	138.752	3.0306	1.20E-05	0.0032499	PREDICTED: Erinaceus europaeus myosin-1 (LOC103113192), transcript variant X1, mRNA
Cluster-33503.48403	MYH	223.506	22.713	3.3089	7.05E-05	0.015504	PREDICTED: Erinaceus europaeus myosin-1 (LOC103113192), transcript variant X2, mRNA
Cluster-33503.50727	MYH	251.549	33.225	2.9295	9.54E-05	0.020087	PREDICTED: Erinaceus europaeus myosin-1 (LOC103113192), transcript variant X2, mRNA
Cluster-33503.123878	MYH	224.516	26.567	3.0897	0.00014529	0.028734	PREDICTED: Erinaceus europaeus myosin-1 (LOC103113192), transcript variant X2, mRNA
Cluster-33503.45922	MYBPC2	340.383	37.394	3.1906	3.59E-06	0.0010989	PREDICTED: Erinaceus europaeus myosin binding protein C, fast type (MYBPC2), mRNA
Cluster-33503.81601	MXD, MAD	510.073	1560.119	-1.6119	0.00015284	0.030031	PREDICTED: Rousettus aegyptiacus MAX dimerization protein 1 (MXD1), mRNA
Cluster-33503.11109	MRVI1, IRAG	85.387	1056.425	-3.6269	2.16E-18	8.88E-15	PREDICTED: Erinaceus europaeus murine retrovirus integration site 1 homolog (MRVI1), mRNA
Cluster-33503.139349	MRGPRX	130.749	30.336	2.1139	0.00012977	0.026044	PREDICTED: Erinaceus europaeus mas-related G-protein coupled receptor member X4-like (LOC103109608), mRNA
Cluster-33503.38775	MRGPRX	379.315	97.493	1.9609	0.00023618	0.043363	TPA_inf: Erinaceus europaeus MGRG1 gene for Mas-related G protein-coupled receptor G1
Cluster-33503.106875	MHC2	1796.490	384.029	2.2261	7.67E-05	0.016659	PREDICTED: Erinaceus europaeus DLA class II histocompatibility antigen, DR-1 beta chain-like (LOC103117417), mRNA
Cluster-6154.0	maiA, GSTZ1	0.000	21.297	-6.9237	8.10E-06	0.0022719	PREDICTED: Gossypium raimondii glutathione S-transferase U17-like (LOC105804293), misc_RNA

Cluster-33503.60736	LTB, TNFC	267.995	22.553	3.5735	6.87E-06	0.001968	PREDICTED: Erinaceus europaeus lymphotoxin beta (LTB), transcript variant X1, mRNA
Cluster-33503.112271	LIPH_I	125.864	625.092	-2.3109	8.20E-07	0.00028687	PREDICTED: Erinaceus europaeus lipase H (LIPH), mRNA
Cluster-33503.97351	KRT2	26.190	1428.048	-5.7862	3.32E-31	1.29E-26	PREDICTED: Erinaceus europaeus keratin, type II cytoskeletal 6A (LOC103109181), mRNA
Cluster-33503.72554	KRT2	30.027	1856.636	-5.9706	3.57E-28	9.70E-24	PREDICTED: Erinaceus europaeus keratin, type II cytoskeletal 6A (LOC103109181), mRNA
Cluster-33503.72555	KRT2	134.375	8305.814	-5.9539	5.04E-28	1.16E-23	PREDICTED: Erinaceus europaeus keratin, type II cytoskeletal 6A (LOC103109181), mRNA
Cluster-33503.72567	KRT2	583.696	35400.322	-5.9236	5.11E-28	1.16E-23	PREDICTED: Erinaceus europaeus keratin, type II cytoskeletal 6A (LOC103109181), mRNA
Cluster-33503.72548	KRT2	431.294	25509.208	-5.8877	1.11E-27	2.14E-23	PREDICTED: Erinaceus europaeus keratin, type II cytoskeletal 6A (LOC103109181), mRNA
Cluster-33503.72552	KRT2	1422.400	81701.264	-5.8445	2.78E-27	5.04E-23	PREDICTED: Erinaceus europaeus keratin, type II cytoskeletal 6A (LOC103109181), mRNA
Cluster-33503.109240	KRT2	4.477	674.808	-7.2703	1.08E-26	1.72E-22	PREDICTED: Erinaceus europaeus keratin, type II cytoskeletal 6A (LOC103109181), mRNA
Cluster-33503.72574	KRT2	13.875	924.353	-6.0852	1.04E-26	1.72E-22	PREDICTED: Erinaceus europaeus keratin, type II cytoskeletal 6A (LOC103109181), mRNA
Cluster-33503.177708	KRT2	45.039	2056.814	-5.5262	2.50E-26	3.39E-22	PREDICTED: Erinaceus europaeus keratin, type II cytoskeletal 6A (LOC103109181), mRNA
Cluster-33503.126636	KRT2	349.852	15541.459	-5.4746	7.67E-25	9.05E-21	PREDICTED: Erinaceus europaeus keratin, type II cytoskeletal 75-like (LOC103109183), mRNA
Cluster-33503.66635	KRT2	30.087	1162.164	-5.2857	1.69E-24	1.84E-20	PREDICTED: Erinaceus europaeus keratin, type II cytoskeletal 6A (LOC103109181), mRNA
Cluster-33503.107316	KRT2	9.077	612.642	-6.1188	1.82E-23	1.83E-19	PREDICTED: Erinaceus europaeus keratin, type II cytoskeletal 6A-like (LOC103109273), mRNA

Cluster-33503.69006	KRT2	591.098	27755.974	-5.5543	5.38E-23	5.22E-19	PREDICTED: Erinaceus europaeus keratin, type II cytoskeletal 6A (LOC103109181), mRNA
Cluster-33503.47332	KRT2	80.616	2572.705	-5.0025	6.55E-23	6.13E-19	PREDICTED: Erinaceus europaeus keratin, type II cytoskeletal 75-like (LOC103109183), mRNA
Cluster-33503.42601	KRT2	19.734	668.180	-5.0892	1.03E-22	8.98E-19	PREDICTED: Erinaceus europaeus keratin, type II cytoskeletal 75-like (LOC103109183), mRNA
Cluster-33503.123645	KRT2	88.524	3277.389	-5.2176	1.07E-21	6.76E-18	PREDICTED: Erinaceus europaeus keratin, type II cytoskeletal 75-like (LOC103109183), mRNA
Cluster-33503.122547	KRT2	38.024	848.811	-4.49	3.33E-21	1.96E-17	PREDICTED: Erinaceus europaeus keratin, type II cuticular Hb6 (LOC103109202), mRNA
Cluster-33503.69001	KRT2	9.617	514.592	-5.775	3.97E-21	2.25E-17	PREDICTED: Erinaceus europaeus keratin, type II cytoskeletal 6A (LOC103109181), mRNA
Cluster-33503.68999	KRT2	3.919	319.845	-6.3977	1.57E-20	8.72E-17	PREDICTED: Erinaceus europaeus keratin, type II cytoskeletal 6A (LOC103109181), mRNA
Cluster-33503.68993	KRT2	10.895	498.207	-5.5564	7.90E-20	4.13E-16	PREDICTED: Erinaceus europaeus keratin, type II cytoskeletal 6A-like (LOC103109273), mRNA
Cluster-33503.125317	KRT2	468.300	13418.300	-4.8419	8.50E-20	4.27E-16	PREDICTED: Erinaceus europaeus keratin, type II cytoskeletal 75-like (LOC103109183), mRNA
Cluster-33503.69000	KRT2	4.537	434.366	-6.7045	3.02E-19	1.41E-15	PREDICTED: Erinaceus europaeus keratin, type II cytoskeletal 6A (LOC103109181), mRNA
Cluster-33503.72556	KRT2	7.734	529.340	-6.1745	3.90E-19	1.79E-15	PREDICTED: Erinaceus europaeus keratin, type II cytoskeletal 6A (LOC103109181), mRNA
Cluster-33503.126633	KRT2	24.356	714.694	-4.8979	1.11E-18	4.79E-15	PREDICTED: Erinaceus europaeus keratin, type II cytoskeletal 75-like (LOC103109183), mRNA
Cluster-33503.121996	KRT2	77.314	1223.229	-3.9893	1.62E-18	6.75E-15	PREDICTED: Erinaceus europaeus keratin, type II microfibrillar, component 7C (LOC103109188), mRNA

Cluster-33503.109507	KRT2	5.637	256.597	-5.5588	3.48E-17	1.24E-13	PREDICTED: Erinaceus europaeus keratin, type II cytoskeletal 6A (LOC103109181), mRNA
Cluster-33503.133783	KRT2	7.595	297.333	-5.3504	9.54E-17	3.22E-13	PREDICTED: Erinaceus europaeus keratin, type II cytoskeletal 6A (LOC103109181), mRNA
Cluster-33503.72549	KRT2	8.833	406.876	-5.583	1.39E-16	4.49E-13	PREDICTED: Erinaceus europaeus keratin, type II cytoskeletal 6A-like (LOC103109273), mRNA
Cluster-33503.48042	KRT2	4.578	209.281	-5.5604	2.16E-16	6.73E-13	PREDICTED: Erinaceus europaeus keratin, type II cytoskeletal 75-like (LOC103109183), mRNA
Cluster-33503.67794	KRT2	40.772	1765.577	-5.4529	3.18E-16	9.60E-13	PREDICTED: Erinaceus europaeus keratin, type II cytoskeletal 6A (LOC103109181), mRNA
Cluster-33503.69003	KRT2	1.640	181.164	-6.6776	9.73E-16	2.75E-12	PREDICTED: Erinaceus europaeus keratin, type II cytoskeletal 6A (LOC103109181), mRNA
Cluster-33503.45606	KRT2	13.575	384.908	-4.8573	1.84E-15	4.85E-12	PREDICTED: Erinaceus europaeus keratin, type II cytoskeletal 75-like (LOC103109183), mRNA
Cluster-33503.49535	KRT2	32.211	651.668	-4.3569	1.88E-15	4.90E-12	PREDICTED: Erinaceus europaeus keratin, type II cytoskeletal 75-like (LOC103109183), mRNA
Cluster-33503.128278	KRT2	1607.201	21027.351	-3.71	3.99E-15	9.94E-12	PREDICTED: Erinaceus europaeus keratin 5 (KRT5), mRNA
Cluster-33503.129472	KRT2	6.419	232.771	-5.2292	6.21E-15	1.52E-11	PREDICTED: Erinaceus europaeus keratin, type II cytoskeletal 75-like (LOC103109183), mRNA
Cluster-33503.47330	KRT2	9.758	281.424	-4.8858	1.28E-14	2.90E-11	PREDICTED: Erinaceus europaeus keratin, type II cytoskeletal 75-like (LOC103109183), mRNA
Cluster-33503.71611	KRT2	4.179	166.167	-5.3469	2.99E-14	6.29E-11	PREDICTED: Erinaceus europaeus keratin, type II cytoskeletal 6A-like (LOC103109273), mRNA
Cluster-33503.49329	KRT2	257.148	3544.650	-3.7879	4.07E-14	8.32E-11	PREDICTED: Erinaceus europaeus keratin, type II microfibrillar, component 7C (LOC103109188), mRNA

Cluster-33503.123013	KRT2	70.994	992.147	-3.8143	5.96E-14	1.16E-10	PREDICTED: Erinaceus europaeus keratin, type II microfibrillar, component 7C (LOC103109188), mRNA
Cluster-33503.177733	KRT2	11703.900	165621.743	-3.8229	7.08E-14	1.34E-10	PREDICTED: Erinaceus europaeus keratin, type II microfibrillar, component 7C (LOC103109188), mRNA
Cluster-33503.121990	KRT2	450.196	5644.659	-3.6499	1.04E-13	1.86E-10	PREDICTED: Erinaceus europaeus keratin, type II microfibrillar, component 7C (LOC103109188), mRNA
Cluster-33503.49979	KRT2	71.691	1087.738	-3.9261	2.12E-13	3.52E-10	PREDICTED: Erinaceus europaeus keratin, type II cuticular Hb6 (LOC103109202), mRNA
Cluster-33503.104018	KRT2	1.559	156.471	-6.8848	2.54E-13	4.15E-10	PREDICTED: Erinaceus europaeus keratin, type II cytoskeletal 6A-like (LOC103109182), mRNA
Cluster-33503.109239	KRT2	2.540	120.183	-5.5971	3.21E-13	5.15E-10	PREDICTED: Erinaceus europaeus keratin, type II cytoskeletal 6A (LOC103109181), mRNA
Cluster-33503.47328	KRT2	6.875	247.106	-5.2205	4.74E-13	7.32E-10	PREDICTED: Erinaceus europaeus keratin, type II cytoskeletal 75-like (LOC103109183), mRNA
Cluster-33503.121997	KRT2	651.950	8471.407	-3.7009	5.09E-13	7.82E-10	PREDICTED: Erinaceus europaeus keratin, type II microfibrillar, component 7C (LOC103109188), mRNA
Cluster-33503.178333	KRT2	4.779	184.880	-5.3709	5.58E-13	8.42E-10	PREDICTED: Erinaceus europaeus keratin, type II cytoskeletal 6A-like (LOC103109273), mRNA
Cluster-33503.49836	KRT2	2113.102	26680.320	-3.6587	5.57E-13	8.42E-10	PREDICTED: Erinaceus europaeus keratin, type II microfibrillar, component 7C (LOC103109188), mRNA
Cluster-33503.122008	KRT2	6592.402	85127.252	-3.6909	6.56E-13	9.64E-10	PREDICTED: Erinaceus europaeus keratin, type II cuticular Hb1 (LOC103109277), mRNA

Cluster-33503.122549	KRT2	9274.858	126383.874	-3.7684	7.06E-13	1.03E-09	PREDICTED: Erinaceus europaeus keratin, type II microfibrillar, component 7C (LOC103109188), mRNA
Cluster-33503.121985	KRT2	34.331	535.889	-3.9835	1.29E-12	1.77E-09	PREDICTED: Erinaceus europaeus keratin, type II microfibrillar, component 7C (LOC103109188), mRNA
Cluster-33503.122006	KRT2	83.700	1125.844	-3.7578	1.47E-12	1.96E-09	PREDICTED: Erinaceus europaeus keratin, type II microfibrillar, component 7C (LOC103109188), mRNA
Cluster-33503.121989	KRT2	738.007	9885.771	-3.7447	2.28E-12	2.95E-09	PREDICTED: Erinaceus europaeus keratin, type II microfibrillar, component 7C (LOC103109188), mRNA
Cluster-33503.45605	KRT2	1.100	124.293	-6.4803	2.54E-12	3.23E-09	PREDICTED: Erinaceus europaeus keratin, type II cytoskeletal 75-like (LOC103109183), mRNA
Cluster-33503.121994	KRT2	613.473	8485.205	-3.7912	3.17E-12	4.00E-09	PREDICTED: Erinaceus europaeus keratin, type II cuticular Hb6 (LOC103109202), mRNA
Cluster-33503.50559	KRT2	103.101	1661.892	-4.0174	3.22E-12	4.03E-09	PREDICTED: Erinaceus europaeus keratin, type II microfibrillar, component 7C (LOC103109188), mRNA
Cluster-33503.123625	KRT2	548.821	5934.715	-3.4359	6.31E-12	7.48E-09	PREDICTED: Erinaceus europaeus keratin, type II microfibrillar, component 7C (LOC103109188), mRNA
Cluster-33503.123555	KRT2	428.139	5456.877	-3.6736	7.60E-12	8.90E-09	PREDICTED: Papio anubis keratin 81 (KRT81), mRNA
Cluster-33503.122084	KRT2	61.417	771.683	-3.6632	8.72E-12	1.01E-08	PREDICTED: Erinaceus europaeus keratin, type II microfibrillar, component 7C (LOC103109188), mRNA

Cluster-33503.123512	KRT2	9.077	145.225	-4.0448	9.92E-12	1.12E-08	PREDICTED: Erinaceus europaeus keratin, type II microfibrillar, component 7C (LOC103109188), mRNA
Cluster-33503.48374	KRT2	1120.057	13221.084	-3.5619	1.05E-11	1.17E-08	PREDICTED: Erinaceus europaeus keratin, type II cuticular Hb6 (LOC103109202), mRNA
Cluster-33503.123533	KRT2	344.812	5418.360	-3.976	1.31E-11	1.44E-08	PREDICTED: Erinaceus europaeus keratin, type II cuticular Hb6 (LOC103109202), mRNA
Cluster-33503.50254	KRT2	5879.870	70205.196	-3.5779	1.70E-11	1.82E-08	PREDICTED: Erinaceus europaeus keratin, type II microfibrillar, component 7C (LOC103109188), mRNA
Cluster-33503.122001	KRT2	794.391	10139.352	-3.6749	1.74E-11	1.85E-08	PREDICTED: Erinaceus europaeus keratin, type II microfibrillar, component 7C (LOC103109188), mRNA
Cluster-33503.47331	KRT2	4.298	121.185	-4.8236	2.04E-11	2.10E-08	PREDICTED: Erinaceus europaeus keratin, type II cytoskeletal 75-like (LOC103109183), mRNA
Cluster-33503.122000	KRT2	4440.275	50695.077	-3.5133	2.25E-11	2.28E-08	PREDICTED: Erinaceus europaeus keratin, type II microfibrillar, component 7C (LOC103109188), mRNA
Cluster-33503.123532	KRT2	31.105	400.308	-3.7085	2.41E-11	2.42E-08	PREDICTED: Erinaceus europaeus keratin, type II cuticular Hb1 (LOC103109277), mRNA
Cluster-33503.122370	KRT2	513.452	6203.788	-3.5962	2.67E-11	2.65E-08	PREDICTED: Erinaceus europaeus keratin, type II cuticular Hb6 (LOC103109202), mRNA
Cluster-33503.122177	KRT2	623.414	9022.755	-3.8565	2.99E-11	2.89E-08	PREDICTED: Erinaceus europaeus keratin, type II cuticular Hb6 (LOC103109202), mRNA
Cluster-33503.50157	KRT2	1755.314	24059.526	-3.7772	3.74E-11	3.54E-08	PREDICTED: Erinaceus europaeus keratin, type II cuticular Hb6 (LOC103109202), mRNA
Cluster-33503.121986	KRT2	9333.617	133976.808	-3.8435	3.93E-11	3.69E-08	PREDICTED: Myotis brandtii keratin, type II cuticular Hb1-like (LOC102261888), mRNA

Cluster-33503.122583	KRT2	1017.775	10709.966	-3.3961	4.77E-11	4.43E-08	PREDICTED: Erinaceus europaeus keratin, type II microfibrillar, component 7C (LOC103109188), mRNA
Cluster-33503.127116	KRT2	443.173	4819.942	-3.4446	5.24E-11	4.79E-08	PREDICTED: Erinaceus europaeus keratin, type II cuticular Hb6 (LOC103109202), mRNA
Cluster-33503.121999	KRT2	2051.743	25133.265	-3.6151	6.30E-11	5.66E-08	PREDICTED: Erinaceus europaeus keratin, type II cuticular Hb6 (LOC103109202), mRNA
Cluster-33503.123453	KRT2	210.274	2855.251	-3.7665	6.87E-11	6.14E-08	PREDICTED: Erinaceus europaeus keratin, type II cuticular Hb1 (LOC103109277), mRNA
Cluster-33503.126105	KRT2	50.492	579.064	-3.5294	7.25E-11	6.45E-08	PREDICTED: Erinaceus europaeus keratin, type II cuticular Hb1 (LOC103109277), mRNA
Cluster-33503.122600	KRT2	699.576	7144.663	-3.3533	7.61E-11	6.73E-08	PREDICTED: Erinaceus europaeus keratin, type II cuticular Hb6 (LOC103109202), mRNA
Cluster-33503.49090	KRT2	13.693	194.505	-3.8656	7.73E-11	6.82E-08	PREDICTED: Erinaceus europaeus keratin, type II cuticular Hb1 (LOC103109277), mRNA
Cluster-33503.122590	KRT2	1361.092	15627.437	-3.5218	8.25E-11	7.23E-08	PREDICTED: Erinaceus europaeus keratin, type II microfibrillar, component 7C (LOC103109188), mRNA
Cluster-33503.49091	KRT2	10.198	129.578	-3.7004	8.80E-11	7.66E-08	PREDICTED: Erinaceus europaeus keratin, type II cuticular Hb1 (LOC103109277), mRNA
Cluster-33503.45604	KRT2	6.980	184.218	-4.7889	1.07E-10	9.18E-08	PREDICTED: Erinaceus europaeus keratin, type II cytoskeletal 75-like (LOC103109183), mRNA
Cluster-33503.122446	KRT2	137.318	1903.050	-3.798	1.33E-10	1.12E-07	PREDICTED: Erinaceus europaeus keratin, type II cuticular Hb6 (LOC103109202), mRNA
Cluster-33503.49180	KRT2	113.812	1029.996	-3.1836	1.49E-10	1.23E-07	PREDICTED: Erinaceus europaeus keratin, type II cuticular Hb5 (LOC103109186), mRNA

Cluster-33503.122546	KRT2	652.647	7379.385	-3.5003	1.54E-10	1.28E-07	PREDICTED: Erinaceus europaeus keratin, type II microfibrillar, component 7C (LOC103109188), mRNA
Cluster-33503.122607	KRT2	1020.401	11191.181	-3.4559	1.57E-10	1.30E-07	PREDICTED: Erinaceus europaeus keratin, type II cuticular Hb6 (LOC103109202), mRNA
Cluster-33503.122389	KRT2	16.011	263.669	-4.0783	1.83E-10	1.49E-07	PREDICTED: Erinaceus europaeus keratin, type II microfibrillar, component 7C (LOC103109188), mRNA
Cluster-33503.123103	KRT2	51.760	664.653	-3.6953	1.84E-10	1.50E-07	PREDICTED: Erinaceus europaeus keratin, type II cuticular Hb6 (LOC103109202), mRNA
Cluster-33503.122578	KRT2	32.383	449.877	-3.8158	1.88E-10	1.51E-07	PREDICTED: Erinaceus europaeus keratin, type II microfibrillar, component 7C (LOC103109188), mRNA
Cluster-33503.42373	KRT2	318.209	2116.396	-2.7354	2.00E-10	1.59E-07	PREDICTED: Erinaceus europaeus keratin, type II cuticular Hb5 (LOC103109186), mRNA
Cluster-33503.128407	KRT2	43.673	677.391	-3.971	2.07E-10	1.63E-07	PREDICTED: Erinaceus europaeus keratin, type II cuticular Hb6 (LOC103109202), mRNA
Cluster-33503.123626	KRT2	33.569	429.038	-3.6967	2.54E-10	1.96E-07	PREDICTED: Bubalus bubalis keratin, type II microfibrillar, component 5-like (LOC102389337), mRNA
Cluster-33503.49485	KRT2	415.966	5889.362	-3.8254	2.65E-10	2.02E-07	PREDICTED: Erinaceus europaeus keratin, type II cuticular Hb6 (LOC103109202), mRNA
Cluster-33503.48742	KRT2	118.213	1301.074	-3.4659	2.68E-10	2.03E-07	PREDICTED: Erinaceus europaeus keratin, type II cuticular Hb6 (LOC103109202), mRNA
Cluster-33503.122585	KRT2	147.824	1655.136	-3.4901	3.09E-10	2.32E-07	PREDICTED: Erinaceus europaeus keratin, type II microfibrillar, component 7C (LOC103109188), mRNA

Cluster-33503.48085	KRT2	4.239	91.470	-4.5235	3.17E-10	2.36E-07	PREDICTED: Erinaceus europaeus keratin, type II microfibrillar, component 7C (LOC103109188), mRNA
Cluster-33503.122545	KRT2	2017.797	24279.225	-3.5893	3.43E-10	2.53E-07	PREDICTED: Erinaceus europaeus keratin, type II microfibrillar, component 7C (LOC103109188), mRNA
Cluster-33503.50234	KRT2	242.516	3156.288	-3.7052	3.51E-10	2.57E-07	PREDICTED: Erinaceus europaeus keratin, type II microfibrillar, component 7C (LOC103109188), mRNA
Cluster-33503.50306	KRT2	148.706	1915.686	-3.6925	3.84E-10	2.80E-07	PREDICTED: Erinaceus europaeus keratin, type II microfibrillar, component 7C (LOC103109188), mRNA
Cluster-33503.48024	KRT2	104.593	716.521	-2.7815	4.16E-10	3.01E-07	PREDICTED: Erinaceus europaeus keratin, type II cuticular Hb5 (LOC103109186), mRNA
Cluster-33503.122821	KRT2	361.549	5077.623	-3.8139	4.36E-10	3.13E-07	PREDICTED: Erinaceus europaeus keratin, type II cuticular Hb6 (LOC103109202), mRNA
Cluster-33503.177864	KRT2	64.798	706.203	-3.4579	4.45E-10	3.18E-07	PREDICTED: Erinaceus europaeus keratin, type II cuticular Hb6 (LOC103109202), mRNA
Cluster-33503.122552	KRT2	102.422	1105.022	-3.4386	4.56E-10	3.25E-07	PREDICTED: Erinaceus europaeus keratin, type II microfibrillar, component 7C (LOC103109188), mRNA
Cluster-33503.121992	KRT2	94.735	1107.399	-3.5551	4.71E-10	3.34E-07	PREDICTED: Erinaceus europaeus keratin, type II microfibrillar, component 7C (LOC103109188), mRNA
Cluster-33503.122591	KRT2	83.775	961.760	-3.5289	4.85E-10	3.41E-07	PREDICTED: Erinaceus europaeus keratin, type II cuticular Hb6 (LOC103109202), mRNA
Cluster-33503.122594	KRT2	1119.246	14866.289	-3.7321	5.40E-10	3.77E-07	PREDICTED: Erinaceus europaeus keratin, type II cuticular Hb1 (LOC103109277), mRNA

Cluster-33503.50129	KRT2	8.655	130.528	-3.954	5.64E-10	3.91E-07	PREDICTED: Erinaceus europaeus keratin, type II microfibrillar, component 7C (LOC103109188), mRNA
Cluster-33503.122077	KRT2	370.974	4873.691	-3.7176	5.86E-10	4.05E-07	PREDICTED: Erinaceus europaeus keratin, type II cuticular Hb6 (LOC103109202), mRNA
Cluster-33503.124482	KRT2	15.370	234.509	-3.9413	6.19E-10	4.25E-07	PREDICTED: Erinaceus europaeus keratin, type II cuticular Hb1 (LOC103109277), mRNA
Cluster-33503.122404	KRT2	34.146	344.927	-3.3576	7.46E-10	5.00E-07	PREDICTED: Erinaceus europaeus keratin, type II microfibrillar, component 7C (LOC103109188), mRNA
Cluster-33503.122579	KRT2	15.453	209.326	-3.8016	9.58E-10	6.32E-07	PREDICTED: Erinaceus europaeus keratin, type II microfibrillar, component 7C (LOC103109188), mRNA
Cluster-33503.122007	KRT2	1404.430	8242.253	-2.5532	9.82E-10	6.44E-07	PREDICTED: Erinaceus europaeus keratin, type II cuticular Hb5 (LOC103109186), mRNA
Cluster-33503.49602	KRT2	691.795	9350.033	-3.7577	1.02E-09	6.65E-07	PREDICTED: Erinaceus europaeus keratin, type II cuticular Hb6 (LOC103109202), mRNA
Cluster-33503.122593	KRT2	12.899	168.022	-3.7334	1.42E-09	9.00E-07	PREDICTED: Erinaceus europaeus keratin, type II cuticular Hb6 (LOC103109202), mRNA
Cluster-33503.122820	KRT2	66.963	697.120	-3.3903	1.69E-09	1.06E-06	PREDICTED: Erinaceus europaeus keratin, type II cuticular Hb1 (LOC103109277), mRNA
Cluster-33503.127418	KRT2	3.017	90.756	-5.0584	1.71E-09	1.07E-06	PREDICTED: Erinaceus europaeus keratin, type II cuticular Hb1 (LOC103109277), mRNA
Cluster-33503.122232	KRT2	15.213	172.883	-3.5437	1.81E-09	1.13E-06	PREDICTED: Ailuropoda melanoleuca keratin, type II cuticular Hb1-like (LOC100480853), mRNA
Cluster-33503.123172	KRT2	18.530	192.639	-3.4054	2.02E-09	1.24E-06	PREDICTED: Erinaceus europaeus keratin, type II cuticular Hb6 (LOC103109202), mRNA

Cluster-33503.122605	KRT2	105.941	1038.659	-3.3001	2.32E-09	1.41E-06	PREDICTED: Erinaceus europaeus keratin, type II microfibrillar, component 7C (LOC103109188), mRNA
Cluster-33503.49971	KRT2	153.340	1517.752	-3.312	2.44E-09	1.48E-06	PREDICTED: Erinaceus europaeus keratin, type II microfibrillar, component 7C (LOC103109188), mRNA
Cluster-33503.124637	KRT2	343.832	4297.790	-3.646	3.41E-09	1.98E-06	PREDICTED: Erinaceus europaeus keratin, type II cuticular Hb6 (LOC103109202), mRNA
Cluster-33503.123007	KRT2	474.954	6469.383	-3.7693	4.00E-09	2.30E-06	PREDICTED: Erinaceus europaeus keratin, type II cuticular Hb1 (LOC103109277), mRNA
Cluster-33503.122670	KRT2	7.637	112.583	-3.9007	5.46E-09	3.03E-06	PREDICTED: Erinaceus europaeus keratin, type II cuticular Hb6 (LOC103109202), mRNA
Cluster-33503.121652	KRT2	0.360	89.832	-8.0401	5.74E-09	3.17E-06	PREDICTED: Erinaceus europaeus keratin, type II cytoskeletal 6A-like (LOC103109273), mRNA
Cluster-33503.125351	KRT2	53.233	707.933	-3.7467	6.01E-09	3.31E-06	PREDICTED: Erinaceus europaeus keratin, type II cuticular Hb6 (LOC103109202), mRNA
Cluster-33503.124371	KRT2	55.670	607.437	-3.4609	6.69E-09	3.64E-06	PREDICTED: Erinaceus europaeus keratin, type II cuticular Hb6 (LOC103109202), mRNA
Cluster-33503.127248	KRT2	12.216	150.175	-3.6689	7.50E-09	4.06E-06	PREDICTED: Erinaceus europaeus keratin, type II microfibrillar, component 7C (LOC103109188), mRNA
Cluster-33503.27354	KRT2	421.919	4341.242	-3.365	8.18E-09	4.42E-06	--
Cluster-33503.48997	KRT2	69.356	434.883	-2.6529	8.76E-09	4.69E-06	PREDICTED: Erinaceus europaeus keratin, type II cuticular Hb5 (LOC103109186), mRNA
Cluster-33503.122014	KRT2	4.678	83.125	-4.2429	8.84E-09	4.73E-06	PREDICTED: Erinaceus europaeus keratin, type II microfibrillar, component 7C (LOC103109188), mRNA

Cluster-33503.122011	KRT2	34.879	385.458	-3.4818	9.00E-09	4.80E-06	PREDICTED: Erinaceus europaeus keratin, type II microfibrillar, component 7C (LOC103109188), mRNA
Cluster-33503.125400	KRT2	166.868	972.175	-2.5456	9.23E-09	4.91E-06	PREDICTED: Erinaceus europaeus keratin, type II cuticular Hb5 (LOC103109186), mRNA
Cluster-33503.49459	KRT2	72.939	810.439	-3.4843	9.61E-09	5.08E-06	PREDICTED: Erinaceus europaeus keratin, type II cuticular Hb6 (LOC103109202), mRNA
Cluster-33503.50305	KRT2	58.959	760.820	-3.7002	1.07E-08	5.58E-06	PREDICTED: Erinaceus europaeus keratin, type II microfibrillar, component 7C (LOC103109188), mRNA
Cluster-33503.49964	KRT2	199.603	2616.735	-3.7162	1.17E-08	6.05E-06	PREDICTED: Erinaceus europaeus keratin, type II cuticular Hb1 (LOC103109277), mRNA
Cluster-33503.7489	KRT2	19.710	196.187	-3.329	1.22E-08	6.30E-06	PREDICTED: Erinaceus europaeus keratin, type II cuticular Hb6 (LOC103109202), mRNA
Cluster-33503.48883	KRT2	148.645	806.065	-2.4426	1.45E-08	7.37E-06	PREDICTED: Erinaceus europaeus keratin, type II cuticular Hb5 (LOC103109186), mRNA
Cluster-33503.122604	KRT2	1.820	67.137	-5.0662	1.47E-08	7.42E-06	PREDICTED: Erinaceus europaeus keratin, type II microfibrillar, component 7C (LOC103109188), mRNA
Cluster-33503.125337	KRT2	24.633	328.042	-3.7606	1.67E-08	8.36E-06	PREDICTED: Erinaceus europaeus keratin, type II microfibrillar, component 7C (LOC103109188), mRNA
Cluster-33503.122430	KRT2	13.275	144.518	-3.484	1.70E-08	8.44E-06	PREDICTED: Erinaceus europaeus keratin, type II microfibrillar, component 7C (LOC103109188), mRNA
Cluster-33503.122399	KRT2	0.000	41.266	-7.8814	1.71E-08	8.48E-06	PREDICTED: Erinaceus europaeus keratin, type II cytoskeletal 6A-like (LOC103109273), mRNA

Cluster-33503.7694	KRT2	76.680	940.387	-3.6253	2.12E-08	1.03E-05	PREDICTED: Erinaceus europaeus keratin, type II cuticular Hb6 (LOC103109202), mRNA
Cluster-33503.123008	KRT2	13.852	141.043	-3.3724	2.80E-08	1.33E-05	PREDICTED: Erinaceus europaeus keratin, type II microfibrillar, component 7C (LOC103109188), mRNA
Cluster-33503.72550	KRT2	1.120	61.695	-5.9756	3.11E-08	1.47E-05	PREDICTED: Erinaceus europaeus keratin, type II cytoskeletal 6A (LOC103109181), mRNA
Cluster-33503.125024	KRT2	176.783	2357.612	-3.7413	4.16E-08	1.93E-05	PREDICTED: Macaca nemestrina keratin 81, type II (KRT81), mRNA
Cluster-33503.122470	KRT2	8.755	112.755	-3.7396	4.92E-08	2.24E-05	PREDICTED: Erinaceus europaeus keratin, type II microfibrillar, component 7C (LOC103109188), mRNA
Cluster-33503.123022	KRT2	42.891	271.806	-2.6765	5.19E-08	2.35E-05	PREDICTED: Erinaceus europaeus keratin, type II cuticular Hb5 (LOC103109186), mRNA
Cluster-33503.49298	KRT2	15.529	238.626	-3.9811	6.67E-08	2.95E-05	PREDICTED: Erinaceus europaeus keratin, type II cuticular Hb6 (LOC103109202), mRNA
Cluster-33503.122601	KRT2	18.130	180.432	-3.3408	7.32E-08	3.20E-05	PREDICTED: Erinaceus europaeus keratin, type II cuticular Hb1 (LOC103109277), mRNA
Cluster-33503.110779	KRT2	0.360	57.427	-7.3937	8.46E-08	3.64E-05	PREDICTED: Erinaceus europaeus keratin, type II cytoskeletal 75-like (LOC103109183), mRNA
Cluster-33503.126942	KRT2	5.776	85.339	-3.9645	1.06E-07	4.46E-05	PREDICTED: Erinaceus europaeus keratin, type II cuticular Hb1 (LOC103109277), mRNA
Cluster-33503.47940	KRT2	25.375	161.949	-2.6862	1.16E-07	4.85E-05	PREDICTED: Erinaceus europaeus keratin, type II cuticular Hb5 (LOC103109186), mRNA
Cluster-33503.148721	KRT2	78.352	443.964	-2.5107	1.78E-07	7.12E-05	PREDICTED: Erinaceus europaeus keratin, type II cuticular Hb5 (LOC103109186), mRNA
Cluster-33503.122260	KRT2	25.834	163.686	-2.6818	2.09E-07	8.28E-05	--

Cluster-33503.17615	KRT2	13.916	136.900	-3.3379	2.13E-07	8.43E-05	PREDICTED: Erinaceus europaeus keratin, type II microfibrillar, component 7C (LOC103109188), mRNA
Cluster-33503.159458	KRT2	15.092	161.435	-3.4624	2.21E-07	8.67E-05	PREDICTED: Erinaceus europaeus keratin, type II microfibrillar, component 7C (LOC103109188), mRNA
Cluster-33503.50342	KRT2	74.635	376.995	-2.3417	2.54E-07	9.79E-05	PREDICTED: Erinaceus europaeus keratin, type II cuticular Hb5 (LOC103109186), mRNA
Cluster-33503.126637	KRT2	2.739	59.321	-4.3639	2.70E-07	0.00010352	PREDICTED: Erinaceus europaeus keratin, type II cytoskeletal 75-like (LOC103109183), mRNA
Cluster-33503.124729	KRT2	21.944	233.955	-3.4417	2.78E-07	0.00010621	PREDICTED: Erinaceus europaeus keratin, type II microfibrillar, component 7C (LOC103109188), mRNA
Cluster-33503.122785	KRT2	13.095	107.172	-3.0441	3.20E-07	0.00012097	PREDICTED: Erinaceus europaeus keratin, type II microfibrillar, component 7C (LOC103109188), mRNA
Cluster-33503.50226	KRT2	38.828	215.067	-2.4744	3.49E-07	0.00013078	PREDICTED: Erinaceus europaeus keratin, type II microfibrillar, component 7C (LOC103109188), mRNA
Cluster-33503.127419	KRT2	0.400	39.528	-6.8565	7.26E-07	0.00025609	PREDICTED: Sus scrofa keratin, type II cuticular Hb1 (LOC100523123), mRNA
Cluster-33503.125709	KRT2	14.434	112.847	-2.9858	8.83E-07	0.00030732	Bos taurus BTA05 BES11_Contig416_1160 genomic sequence contig containing highly polymorphic single nucleotide sites
Cluster-33503.124636	KRT2	71.713	363.291	-2.3463	9.05E-07	0.00031451	PREDICTED: Erinaceus europaeus keratin, type II cuticular Hb5 (LOC103109186), mRNA

Cluster-33503.122009	KRT2	0.000	25.327	-7.1767	1.65E-06	0.00054794	PREDICTED: Erinaceus europaeus keratin, type II microfibrillar, component 7C (LOC103109188), mRNA
Cluster-33503.27378	KRT2	5.297	72.202	-3.8591	2.39E-06	0.00076208	PREDICTED: Erinaceus europaeus keratin, type II cuticular Hb6 (LOC103109202), mRNA
Cluster-33503.126326	KRT2	0.760	38.238	-5.8853	2.60E-06	0.00081866	PREDICTED: Erinaceus europaeus keratin, type II cytoskeletal 75-like (LOC103109183), mRNA
Cluster-33503.145574	KRT2	0.760	37.024	-5.842	2.84E-06	0.00088906	PREDICTED: Erinaceus europaeus keratin, type II cuticular Hb6 (LOC103109202), mRNA
Cluster-33503.119200	KRT2	0.000	22.940	-7.0323	2.92E-06	0.0009115	PREDICTED: Erinaceus europaeus keratin, type II cytoskeletal 6A-like (LOC103109273), mRNA
Cluster-33503.48993	KRT2	6.295	63.854	-3.3798	7.98E-06	0.0022417	PREDICTED: Erinaceus europaeus keratin, type II cuticular Hb1 (LOC103109277), mRNA
Cluster-33503.58180	KRT2	0.000	19.143	-6.7808	1.01E-05	0.0027728	PREDICTED: Erinaceus europaeus keratin, type II cytoskeletal 6A (LOC103109181), mRNA
Cluster-33503.123622	KRT2	10.576	72.242	-2.7919	1.08E-05	0.0029437	PREDICTED: Erinaceus europaeus keratin, type II cuticular Hb5 (LOC103109186), mRNA
Cluster-33503.49896	KRT2	3.738	53.473	-3.9353	1.34E-05	0.0035732	PREDICTED: Erinaceus europaeus keratin, type II microfibrillar, component 7C (LOC103109188), mRNA
Cluster-33503.19642	KRT2	0.000	19.046	-6.7715	1.59E-05	0.0041681	PREDICTED: Erinaceus europaeus keratin, type II cytoskeletal 6A-like (LOC103109273), mRNA
Cluster-33503.49541	KRT2	18.608	164.426	-3.1757	2.15E-05	0.0054172	PREDICTED: Erinaceus europaeus keratin, type II cuticular Hb6 (LOC103109202), mRNA
Cluster-33503.123107	KRT2	5.259	42.037	-3.0355	5.77E-05	0.013002	PREDICTED: Erinaceus europaeus keratin, type II cuticular Hb5 (LOC103109186), mRNA
Cluster-33503.146584	KRT2	16.971	108.201	-2.6763	5.84E-05	0.013138	PREDICTED: Erinaceus europaeus keratin, type II cuticular Hb1 (LOC103109277), mRNA

Cluster-33503.27298	KRT2	36.165	182.234	-2.3311	5.87E-05	0.013175	PREDICTED: Erinaceus europaeus keratin, type II microfibrillar, component 7C (LOC103109188), mRNA
Cluster-33503.72553	KRT2	0.000	14.917	-6.4119	6.77E-05	0.014964	PREDICTED: Erinaceus europaeus keratin, type II cytoskeletal 6A (LOC103109181), mRNA
Cluster-33503.47690	KRT2	16.277	81.847	-2.357	8.62E-05	0.018438	PREDICTED: Erinaceus europaeus keratin, type II cuticular Hb5 (LOC103109186), mRNA
Cluster-33503.43991	KRT2	0.800	27.822	-4.9912	8.89E-05	0.018909	PREDICTED: Erinaceus europaeus keratin, type II cytoskeletal 75-like (LOC103109183), mRNA
Cluster-33503.49089	KRT2	20.791	117.507	-2.5235	9.95E-05	0.020839	PREDICTED: Erinaceus europaeus keratin, type II microfibrillar, component 7C (LOC103109188), mRNA
Cluster-33503.47478	KRT2	0.400	19.072	-5.8013	0.00012595	0.025486	PREDICTED: Sorex araneus keratin, type II microfibrillar, component 7C (LOC101542370), mRNA
Cluster-33503.118690	KRT2	0.000	13.586	-6.2795	0.00015136	0.02987	PREDICTED: Erinaceus europaeus keratin, type II cytoskeletal 6A-like (LOC103109273), mRNA
Cluster-33503.124137	KRT2	4.859	37.227	-3.0011	0.00015642	0.030536	PREDICTED: Erinaceus europaeus keratin, type II cuticular Hb5 (LOC103109186), mRNA
Cluster-33503.48612	KRT2	2.397	35.496	-4.029	0.00022609	0.041821	PREDICTED: Tupaia chinensis keratin, type II cuticular Hb6 (LOC102500804), mRNA
Cluster-33503.57699	KRT1	24.748	3553.943	-7.1796	1.99E-30	6.74E-26	PREDICTED: Erinaceus europaeus keratin, type I cytoskeletal 16 (LOC103124739), mRNA
Cluster-33503.57741	KRT1	103.380	9186.078	-6.4768	6.45E-25	7.96E-21	PREDICTED: Erinaceus europaeus keratin, type I cytoskeletal 16 (LOC103124739), mRNA
Cluster-33503.57691	KRT1	5.838	494.640	-6.4628	2.62E-20	1.42E-16	PREDICTED: Erinaceus europaeus keratin, type I cytoskeletal 14 (LOC103124740), mRNA

Cluster-33503.57688	KRT1	3.080	362.557	-6.9576	8.28E-19	3.63E-15	PREDICTED: Erinaceus europaeus keratin, type I cytoskeletal 16 (LOC103124739), mRNA
Cluster-33503.57742	KRT1	3.778	258.352	-6.1705	1.26E-15	3.38E-12	PREDICTED: Erinaceus europaeus keratin, type I cytoskeletal 16 (LOC103124739), mRNA
Cluster-33503.45991	KRT1	40.676	561.993	-3.7894	2.56E-15	6.61E-12	PREDICTED: Loxodonta africana keratin 15 (KRT15), transcript variant X2, mRNA
Cluster-33503.57715	KRT1	2824.843	39996.142	-3.8238	1.31E-14	2.94E-11	PREDICTED: Erinaceus europaeus keratin, type I cytoskeletal 17 (LOC103124738), mRNA
Cluster-33503.57696	KRT1	1785.756	24545.524	-3.7811	2.12E-14	4.58E-11	PREDICTED: Erinaceus europaeus keratin, type I cytoskeletal 17 (LOC103124738), mRNA
Cluster-33503.57695	KRT1	1746.413	22391.761	-3.6808	4.18E-14	8.47E-11	PREDICTED: Erinaceus europaeus keratin, type I cytoskeletal 17 (LOC103124738), mRNA
Cluster-33503.57709	KRT1	336.083	4538.159	-3.7564	5.48E-14	1.08E-10	PREDICTED: Erinaceus europaeus keratin, type I cytoskeletal 17 (LOC103124738), mRNA
Cluster-33503.57690	KRT1	1.499	125.191	-6.1804	8.12E-14	1.49E-10	PREDICTED: Erinaceus europaeus keratin, type I cytoskeletal 16 (LOC103124739), mRNA
Cluster-33503.57756	KRT1	534.100	6449.782	-3.5949	9.85E-14	1.77E-10	PREDICTED: Erinaceus europaeus keratin, type I cytoskeletal 17 (LOC103124738), mRNA
Cluster-33503.44934	KRT1	66.173	877.326	-3.7333	2.20E-13	3.63E-10	PREDICTED: Erinaceus europaeus keratin, type I cytoskeletal 17 (LOC103124738), mRNA
Cluster-33503.57726	KRT1	42.095	655.421	-3.9709	5.28E-13	8.05E-10	PREDICTED: Erinaceus europaeus keratin, type I cytoskeletal 17 (LOC103124738), mRNA
Cluster-33503.45992	KRT1	92.478	961.587	-3.3843	9.06E-13	1.29E-09	PREDICTED: Sorex araneus keratin 13, type I (KRT13), mRNA
Cluster-33503.57700	KRT1	53.277	1032.227	-4.2854	1.18E-12	1.65E-09	PREDICTED: Erinaceus europaeus keratin, type I cytoskeletal 17 (LOC103124738), mRNA
Cluster-33503.57697	KRT1	262.438	4221.312	-4.0098	1.22E-12	1.70E-09	PREDICTED: Erinaceus europaeus keratin, type I cytoskeletal 17 (LOC103124738), mRNA

Cluster-33503.57707	KRT1	370.811	5999.539	-4.0174	1.24E-12	1.71E-09	PREDICTED: Erinaceus europaeus keratin, type I cytoskeletal 17 (LOC103124738), mRNA
Cluster-33503.57682	KRT1	94.231	1564.408	-4.0585	1.61E-12	2.14E-09	PREDICTED: Erinaceus europaeus keratin, type I cytoskeletal 14 (LOC103124740), mRNA
Cluster-33503.43774	KRT1	159.924	2682.777	-4.0714	2.32E-12	2.98E-09	PREDICTED: Erinaceus europaeus keratin, type I cytoskeletal 17 (LOC103124738), mRNA
Cluster-33503.25981	KRT1	1504.844	83.337	4.1759	2.55E-12	3.23E-09	PREDICTED: Erinaceus europaeus keratin 40 (KRT40), mRNA
Cluster-33503.57702	KRT1	107.334	1596.198	-3.8978	3.37E-12	4.20E-09	PREDICTED: Erinaceus europaeus keratin, type I cytoskeletal 17 (LOC103124738), mRNA
Cluster-33503.57673	KRT1	183.193	2583.401	-3.82	1.14E-11	1.26E-08	PREDICTED: Erinaceus europaeus keratin, type I cytoskeletal 14 (LOC103124740), mRNA
Cluster-33503.57722	KRT1	16.753	247.744	-3.9005	3.70E-11	3.53E-08	PREDICTED: Erinaceus europaeus keratin, type I cytoskeletal 17 (LOC103124738), mRNA
Cluster-33503.57668	KRT1	43.416	555.988	-3.6855	5.19E-11	4.77E-08	PREDICTED: Erinaceus europaeus keratin, type I cytoskeletal 17 (LOC103124738), mRNA
Cluster-33503.57720	KRT1	21.562	417.045	-4.2917	1.59E-10	1.31E-07	PREDICTED: Erinaceus europaeus keratin, type I cytoskeletal 17 (LOC103124738), mRNA
Cluster-33503.57686	KRT1	18.656	322.771	-4.1425	2.20E-10	1.73E-07	PREDICTED: Erinaceus europaeus keratin, type I cytoskeletal 17 (LOC103124738), mRNA
Cluster-33503.57724	KRT1	44.979	471.731	-3.3947	2.33E-10	1.82E-07	PREDICTED: Erinaceus europaeus keratin, type I cytoskeletal 17 (LOC103124738), mRNA
Cluster-33503.57684	KRT1	1384.071	12260.459	-3.1474	2.65E-10	2.02E-07	PREDICTED: Erinaceus europaeus keratin, type I cytoskeletal 17 (LOC103124738), mRNA
Cluster-33503.48193	KRT1	71.369	843.219	-3.5719	5.48E-10	3.82E-07	PREDICTED: Erinaceus europaeus keratin, type I cuticular Ha3-I-like (LOC103124758), mRNA
Cluster-33503.57703	KRT1	7.939	132.193	-4.0876	6.40E-10	4.39E-07	PREDICTED: Erinaceus europaeus keratin, type I cytoskeletal 17 (LOC103124738), mRNA

Cluster-33503.57740	KRT1	67.862	712.881	-3.3993	7.07E-10	4.79E-07	PREDICTED: Erinaceus europaeus keratin, type I cytoskeletal 14 (LOC103124740), mRNA
Cluster-33503.57723	KRT1	6.560	123.932	-4.268	8.53E-10	5.66E-07	PREDICTED: Erinaceus europaeus keratin, type I cytoskeletal 17 (LOC103124738), mRNA
Cluster-33503.49786	KRT1	208.496	2334.400	-3.4881	1.85E-09	1.15E-06	PREDICTED: Erinaceus europaeus keratin, type I cuticular Ha3-I-like (LOC103124758), mRNA
Cluster-33503.126571	KRT1	267.421	3291.039	-3.624	2.02E-09	1.24E-06	PREDICTED: Erinaceus europaeus keratin, type I cuticular Ha3-I-like (LOC103124758), mRNA
Cluster-33503.122753	KRT1	13.391	227.218	-4.1317	3.11E-09	1.82E-06	PREDICTED: Erinaceus europaeus keratin, type I cuticular Ha4-like (LOC103124756), mRNA
Cluster-33503.57646	KRT1	0.620	98.666	-7.333	3.36E-09	1.96E-06	PREDICTED: Erinaceus europaeus keratin, type I cytoskeletal 16 (LOC103124739), mRNA
Cluster-33503.57743	KRT1	230.188	1814.840	-2.9802	5.11E-09	2.85E-06	PREDICTED: Erinaceus europaeus keratin, type I cytoskeletal 14 (LOC103124740), mRNA
Cluster-33503.122767	KRT1	5308.404	65355.119	-3.6221	6.16E-09	3.37E-06	PREDICTED: Equus przewalskii keratin 34 (KRT34), mRNA
Cluster-33503.57714	KRT1	4.601	99.387	-4.4286	6.17E-09	3.37E-06	PREDICTED: Erinaceus europaeus keratin, type I cytoskeletal 17 (LOC103124738), mRNA
Cluster-33503.57718	KRT1	0.620	96.504	-7.2998	1.03E-08	5.41E-06	PREDICTED: Erinaceus europaeus keratin, type I cytoskeletal 16 (LOC103124739), mRNA
Cluster-33503.49081	KRT1	449.584	5174.695	-3.5263	1.14E-08	5.91E-06	PREDICTED: Erinaceus europaeus keratin, type I cuticular Ha3-I-like (LOC103124758), mRNA
Cluster-33503.49787	KRT1	124.048	1192.666	-3.271	1.45E-08	7.37E-06	PREDICTED: Erinaceus europaeus keratin, type I cuticular Ha3-I-like (LOC103124758), mRNA
Cluster-33503.122758	KRT1	42.218	438.103	-3.3901	1.56E-08	7.88E-06	PREDICTED: Erinaceus europaeus keratin, type I cuticular Ha4-like (LOC103124756), mRNA
Cluster-33503.123403	KRT1	25.105	266.354	-3.4283	1.58E-08	7.94E-06	PREDICTED: Erinaceus europaeus keratin, type I cuticular Ha4-like (LOC103124756), mRNA

Cluster-33503.127407	KRT1	11.294	140.645	-3.675	1.61E-08	8.05E-06	PREDICTED: Erinaceus europaeus keratin, type I cuticular Ha3-I (LOC103124759), mRNA
Cluster-33503.122754	KRT1	11.732	157.099	-3.7884	1.70E-08	8.44E-06	PREDICTED: Erinaceus europaeus keratin, type I cuticular Ha3-I-like (LOC103124758), mRNA
Cluster-33503.49334	KRT1	25.301	354.599	-3.8291	1.84E-08	9.07E-06	PREDICTED: Erinaceus europaeus keratin, type I cuticular Ha3-I-like (LOC103124758), mRNA
Cluster-33503.57733	KRT1	2151.863	15590.465	-2.8572	2.05E-08	1.01E-05	PREDICTED: Erinaceus europaeus keratin, type I cytoskeletal 14 (LOC103124740), mRNA
Cluster-33503.127033	KRT1	1564.370	14381.028	-3.201	3.65E-08	1.72E-05	PREDICTED: Erinaceus europaeus keratin, type I cuticular Ha3-I (LOC103124759), mRNA
Cluster-33503.57744	KRT1	385.308	2952.924	-2.9395	3.79E-08	1.77E-05	PREDICTED: Erinaceus europaeus keratin, type I cytoskeletal 14 (LOC103124740), mRNA
Cluster-33503.49785	KRT1	516.161	5585.070	-3.437	4.38E-08	2.02E-05	PREDICTED: Erinaceus europaeus keratin, type I cuticular Ha3-I-like (LOC103124758), mRNA
Cluster-33503.48116	KRT1	24.365	376.204	-3.9686	4.65E-08	2.13E-05	PREDICTED: Erinaceus europaeus keratin, type I cuticular Ha3-I (LOC103124759), mRNA
Cluster-33503.124049	KRT1	18.894	195.214	-3.3962	5.30E-08	2.39E-05	PREDICTED: Erinaceus europaeus keratin, type I cuticular Ha4-like (LOC103124756), mRNA
Cluster-33503.46826	KRT1	51.431	558.715	-3.4537	6.28E-08	2.81E-05	PREDICTED: Erinaceus europaeus keratin, type I cuticular Ha3-I-like (LOC103124758), mRNA
Cluster-33503.125151	KRT1	428.190	3837.489	-3.1654	6.42E-08	2.86E-05	PREDICTED: Erinaceus europaeus keratin, type I cuticular Ha3-I-like (LOC103124758), mRNA
Cluster-33503.177767	KRT1	1252.610	11151.896	-3.1549	6.48E-08	2.88E-05	PREDICTED: Erinaceus europaeus keratin, type I cuticular Ha3-I (LOC103124759), mRNA
Cluster-33503.47151	KRT1	9.217	123.937	-3.7849	6.49E-08	2.88E-05	PREDICTED: Erinaceus europaeus keratin, type I cytoskeletal 14 (LOC103124740), mRNA
Cluster-33503.57753	KRT1	93.934	685.185	-2.8702	7.11E-08	3.12E-05	PREDICTED: Erinaceus europaeus keratin, type I cytoskeletal 14 (LOC103124740), mRNA

Cluster-33503.122683	KRT1	66674.947	620516.464	-3.2183	7.64E-08	3.32E-05	PREDICTED: Erinaceus europaeus keratin, type I cuticular Ha3-I (LOC103124759), mRNA
Cluster-33503.180566	KRT1	8653.503	44099.164	-2.3494	8.87E-08	3.80E-05	PREDICTED: Erinaceus europaeus keratin, type I cytoskeletal 25 (LOC103120609), mRNA
Cluster-33503.123621	KRT1	1.819	50.585	-4.7907	9.22E-08	3.93E-05	PREDICTED: Erinaceus europaeus keratin, type I cuticular Ha3-I (LOC103124759), mRNA
Cluster-33503.49165	KRT1	65.180	719.258	-3.4744	9.53E-08	4.05E-05	PREDICTED: Erinaceus europaeus keratin, type I cuticular Ha3-I-like (LOC103124758), mRNA
Cluster-33503.125881	KRT1	1024.992	6554.764	-2.6775	9.55E-08	4.05E-05	PREDICTED: Erinaceus europaeus keratin, type I cytoskeletal 14 (LOC103124740), mRNA
Cluster-33503.123811	KRT1	2.399	54.979	-4.6066	1.02E-07	4.30E-05	PREDICTED: Erinaceus europaeus keratin, type I cuticular Ha3-I-like (LOC103124758), mRNA
Cluster-33503.20152	KRT1	5.557	78.408	-3.8835	1.20E-07	4.99E-05	PREDICTED: Erinaceus europaeus keratin, type I cuticular Ha3-I-like (LOC103124758), mRNA
Cluster-33503.49181	KRT1	66.166	849.543	-3.6924	1.31E-07	5.37E-05	PREDICTED: Erinaceus europaeus keratin, type I cuticular Ha4-like (LOC103124756), mRNA
Cluster-33503.123165	KRT1	2.618	54.133	-4.4222	1.35E-07	5.51E-05	PREDICTED: Erinaceus europaeus keratin, type I cuticular Ha3-I-like (LOC103124758), mRNA
Cluster-33503.123520	KRT1	60.939	716.454	-3.5665	1.54E-07	6.23E-05	PREDICTED: Erinaceus europaeus keratin, type I cuticular Ha4-like (LOC103124756), mRNA
Cluster-33503.123573	KRT1	26.598	185.001	-2.7832	1.69E-07	6.80E-05	PREDICTED: Erinaceus europaeus keratin, type I cytoskeletal 17 (LOC103124738), mRNA
Cluster-33503.47247	KRT1	165.675	1567.899	-3.2463	2.13E-07	8.43E-05	PREDICTED: Erinaceus europaeus keratin, type I cuticular Ha4-like (LOC103124756), mRNA
Cluster-33503.122752	KRT1	135.157	1504.283	-3.4807	2.16E-07	8.52E-05	PREDICTED: Erinaceus europaeus keratin, type I cuticular Ha3-I-like (LOC103124758), mRNA
Cluster-33503.122757	KRT1	679.303	6914.616	-3.3485	2.16E-07	8.52E-05	PREDICTED: Erinaceus europaeus keratin, type I cuticular Ha3-I (LOC103124759), mRNA

Cluster-33503.49445	KRT1	1099.738	11114.788	-3.3379	2.35E-07	9.17E-05	PREDICTED: Erinaceus europaeus keratin, type I cuticular Ha4-like (LOC103124756), mRNA
Cluster-33503.48926	KRT1	3.237	65.804	-4.4449	3.38E-07	0.00012706	PREDICTED: Erinaceus europaeus keratin, type I cuticular Ha4-like (LOC103124756), mRNA
Cluster-33503.57678	KRT1	37.995	235.317	-2.6373	4.07E-07	0.00015002	PREDICTED: Erinaceus europaeus keratin, type I cytoskeletal 14 (LOC103124740), mRNA
Cluster-33503.49182	KRT1	121.267	1247.552	-3.3684	4.07E-07	0.00015012	PREDICTED: Erinaceus europaeus keratin, type I cuticular Ha4-like (LOC103124756), mRNA
Cluster-33503.122686	KRT1	15.171	163.433	-3.4658	4.16E-07	0.00015299	PREDICTED: Erinaceus europaeus keratin, type I cuticular Ha4-like (LOC103124756), mRNA
Cluster-33503.57725	KRT1	3.361	67.106	-4.3636	4.59E-07	0.00016702	PREDICTED: Erinaceus europaeus keratin, type I cytoskeletal 17 (LOC103124738), mRNA
Cluster-33503.57694	KRT1	0.400	45.535	-7.0589	5.92E-07	0.00021214	PREDICTED: Heterocephalus glaber keratin, type I cytoskeletal 14 (LOC101707650), mRNA
Cluster-33503.48293	KRT1	231.077	2234.729	-3.2767	6.51E-07	0.00023207	PREDICTED: Erinaceus europaeus keratin, type I cuticular Ha4-like (LOC103124756), mRNA
Cluster-33503.57650	KRT1	107.467	686.438	-2.6783	9.12E-07	0.00031626	PREDICTED: Erinaceus europaeus keratin, type I cytoskeletal 14 (LOC103124740), mRNA
Cluster-33503.122679	KRT1	72.955	757.729	-3.3857	9.35E-07	0.00032342	PREDICTED: Erinaceus europaeus keratin, type I cuticular Ha4-like (LOC103124756), mRNA
Cluster-33503.48296	KRT1	20.223	272.028	-3.7789	1.04E-06	0.00035744	PREDICTED: Erinaceus europaeus keratin, type I cuticular Ha3-I-like (LOC103124758), mRNA
Cluster-33503.163410	KRT1	77.754	718.640	-3.2169	1.26E-06	0.00042865	PREDICTED: Erinaceus europaeus keratin, type I cuticular Ha4-like (LOC103124756), mRNA
Cluster-33503.46107	KRT1	15.075	112.167	-2.9081	1.26E-06	0.00042865	PREDICTED: Erinaceus europaeus keratin, type I cytoskeletal 14 (LOC103124740), mRNA
Cluster-33503.57642	KRT1	410.486	3266.772	-2.9937	1.63E-06	0.00054353	PREDICTED: Erinaceus europaeus keratin, type I cytoskeletal 14 (LOC103124740), mRNA

Cluster-33503.122407	KRT1	816.887	4443.414	-2.4442	1.88E-06	0.00061494	PREDICTED: Erinaceus europaeus keratin, type I cytoskeletal 14 (LOC103124740), mRNA
Cluster-33503.2121	KRT1	2.760	45.103	-4.0276	1.90E-06	0.00062044	PREDICTED: Erinaceus europaeus keratin 9 (KRT9), mRNA
Cluster-33503.57730	KRT1	43.552	249.441	-2.5245	1.97E-06	0.00063977	PREDICTED: Erinaceus europaeus keratin, type I cytoskeletal 14 (LOC103124740), mRNA
Cluster-33503.128359	KRT1	117.270	594.291	-2.3451	2.04E-06	0.00066026	PREDICTED: Erinaceus europaeus keratin, type I cytoskeletal 14 (LOC103124740), mRNA
Cluster-33503.57736	KRT1	1907.747	8613.567	-2.1749	2.11E-06	0.00067898	PREDICTED: Erinaceus europaeus keratin, type I cytoskeletal 14 (LOC103124740), mRNA
Cluster-33503.122677	KRT1	806.524	5624.382	-2.8027	2.25E-06	0.00072094	PREDICTED: Erinaceus europaeus keratin, type I cuticular Ha4-like (LOC103124756), mRNA
Cluster-33503.57676	KRT1	2469.670	11079.108	-2.1656	2.35E-06	0.00074948	PREDICTED: Erinaceus europaeus keratin, type I cytoskeletal 14 (LOC103124740), mRNA
Cluster-33503.57649	KRT1	614.926	2980.374	-2.2779	2.54E-06	0.00080395	PREDICTED: Erinaceus europaeus keratin, type I cytoskeletal 14 (LOC103124740), mRNA
Cluster-33503.50470	KRT1	49.360	300.957	-2.6204	3.18E-06	0.00098338	Equus caballus keratin 31 (KRT31), mRNA
Cluster-33503.57727	KRT1	674.959	2918.854	-2.113	3.31E-06	0.0010205	PREDICTED: Erinaceus europaeus keratin, type I cytoskeletal 14 (LOC103124740), mRNA
Cluster-33503.57739	KRT1	1087.789	5618.752	-2.3694	3.31E-06	0.0010205	PREDICTED: Erinaceus europaeus keratin, type I cytoskeletal 14 (LOC103124740), mRNA
Cluster-33503.122770	KRT1	2.339	58.288	-4.5417	3.51E-06	0.0010771	PREDICTED: Erinaceus europaeus keratin, type I cuticular Ha3-I (LOC103124759), mRNA
Cluster-33503.57689	KRT1	1.441	196.257	-7.1485	3.87E-06	0.0011823	PREDICTED: Erinaceus europaeus keratin, type I cytoskeletal 16 (LOC103124739), mRNA
Cluster-33503.57729	KRT1	2618.148	14101.116	-2.4294	3.97E-06	0.0012108	PREDICTED: Erinaceus europaeus keratin, type I cytoskeletal 14 (LOC103124740), mRNA

Cluster-33503.57643	KRT1	75.204	604.572	-3.0161	4.02E-06	0.0012218	PREDICTED: Erinaceus europaeus keratin, type I cytoskeletal 14 (LOC103124740), mRNA
Cluster-33503.130901	KRT1	1426.484	7163.115	-2.3286	4.08E-06	0.001236	PREDICTED: Erinaceus europaeus keratin, type I cytoskeletal 14 (LOC103124740), mRNA
Cluster-33503.122305	KRT1	775.779	4193.206	-2.4352	4.15E-06	0.0012539	PREDICTED: Erinaceus europaeus keratin, type I cytoskeletal 14 (LOC103124740), mRNA
Cluster-33503.148897	KRT1	191.707	787.679	-2.0396	4.89E-06	0.0014466	PREDICTED: Erinaceus europaeus keratin 36 (KRT36), mRNA
Cluster-33503.126413	KRT1	2.760	37.893	-3.7808	6.01E-06	0.0017433	PREDICTED: Erinaceus europaeus keratin, type I cuticular Ha3-I-like (LOC103124758), mRNA
Cluster-33503.57748	KRT1	4.319	52.630	-3.6454	7.00E-06	0.0019984	PREDICTED: Erinaceus europaeus keratin, type I cytoskeletal 17 (LOC103124738), mRNA
Cluster-33503.50311	KRT1	666.973	3009.196	-2.1746	7.11E-06	0.0020271	PREDICTED: Erinaceus europaeus keratin, type I cytoskeletal 14 (LOC103124740), mRNA
Cluster-33503.57648	KRT1	64.867	344.159	-2.4152	1.15E-05	0.0031167	PREDICTED: Erinaceus europaeus keratin, type I cytoskeletal 14 (LOC103124740), mRNA
Cluster-33503.48925	KRT1	4.497	70.165	-4.0405	1.55E-05	0.0040759	PREDICTED: Erinaceus europaeus keratin, type I cuticular Ha3-I (LOC103124759), mRNA
Cluster-33503.57685	KRT1	4.200	50.663	-3.6536	1.73E-05	0.0044785	PREDICTED: Erinaceus europaeus keratin, type I cytoskeletal 17 (LOC103124738), mRNA
Cluster-33503.126513	KRT1	830.481	2854.883	-1.7818	2.03E-05	0.005149	PREDICTED: Erinaceus europaeus keratin, type I cytoskeletal 25 (LOC103120609), mRNA
Cluster-33503.122681	KRT1	13.217	80.218	-2.6206	2.16E-05	0.0054172	PREDICTED: Erinaceus europaeus keratin 35 (KRT35), mRNA
Cluster-33503.57706	KRT1	28395.488	93130.965	-1.7136	3.16E-05	0.0076229	PREDICTED: Erinaceus europaeus keratin, type I cytoskeletal 25 (LOC103120609), mRNA
Cluster-33503.132866	KRT1	80.208	308.114	-1.9436	4.06E-05	0.0095764	PREDICTED: Erinaceus europaeus keratin, type I cytoskeletal 14 (LOC103124740), mRNA

Cluster-33503.126489	KRT1	197.155	642.262	-1.706	4.37E-05	0.010224	PREDICTED: Erinaceus europaeus keratin, type I cytoskeletal 25 (LOC103120609), mRNA
Cluster-33503.123968	KRT1	31.846	139.375	-2.1402	6.73E-05	0.014911	PREDICTED: Erinaceus europaeus keratin, type I cytoskeletal 25 (LOC103120609), mRNA
Cluster-33503.129026	KRT1	220.406	735.893	-1.7414	0.00010005	0.02093	PREDICTED: Erinaceus europaeus keratin, type I cytoskeletal 25 (LOC103120609), mRNA
Cluster-33503.45529	KRT1	1493.343	4476.252	-1.584	0.00020044	0.037928	PREDICTED: Erinaceus europaeus keratin, type I cytoskeletal 25 (LOC103120609), mRNA
Cluster-33503.126488	KRT1	2162.543	6290.710	-1.5406	0.00020387	0.038445	PREDICTED: Erinaceus europaeus keratin, type I cytoskeletal 25 (LOC103120609), mRNA
Cluster-33503.57716	KRT1	1.999	27.494	-3.8361	0.00027636	0.049533	PREDICTED: Erinaceus europaeus keratin, type I cytoskeletal 17 (LOC103124738), mRNA
Cluster-33503.31523	KLK14	15.360	300.688	-4.311	1.43E-11	1.55E-08	PREDICTED: Erinaceus europaeus kallikrein related peptidase 14 (KLK14), mRNA
Cluster-33503.31203	KIF26	173.878	774.294	-2.1565	2.21E-05	0.0055067	PREDICTED: Erinaceus europaeus kinesin family member 26B (KIF26B), mRNA
Cluster-33503.25561	KCNN4	630.533	138.715	2.1844	9.60E-05	0.020178	PREDICTED: Erinaceus europaeus potassium calcium-activated channel subfamily N member 4 (KCNN4), mRNA
Cluster-33503.42068	KCNMB1	57.083	349.783	-2.6279	5.56E-07	0.00020004	PREDICTED: Erinaceus europaeus potassium calcium-activated channel subfamily M regulatory beta subunit 1 (KCNMB1), mRNA
Cluster-33503.184985	KCNK3	8.085	134.118	-4.0733	4.53E-07	0.00016503	PREDICTED: Cebus capucinus imitator potassium two pore domain channel subfamily K member 3 (KCNK3), mRNA
Cluster-33503.184984	KCNK3	3.840	46.322	-3.6694	4.01E-06	0.0012195	PREDICTED: Erinaceus europaeus potassium two pore domain channel subfamily K member 3 (KCNK3), partial mRNA

Cluster-33503.153326	KCNK10	85.197	418.147	-2.3025	1.32E-05	0.0035441	PREDICTED: Erinaceus europaeus potassium two pore domain channel subfamily K member 10 (KCNK10), transcript variant X2, mRNA
Cluster-88396.0	KCNG1	8.578	153.242	-4.1581	6.82E-10	4.64E-07	PREDICTED: Erinaceus europaeus potassium voltage-gated channel modifier subfamily G member 1 (KCNG1), transcript variant X1, mRNA
Cluster-33503.140537	KCNAB1	6.620	100.518	-3.8709	4.87E-09	2.72E-06	PREDICTED: Erinaceus europaeus potassium voltage-gated channel subfamily A member regulatory beta subunit 1 (KCNAB1), transcript variant X3, mRNA
Cluster-84040.0	katE, CAT, catB, srpA	60.701	0.000	8.3298	7.38E-10	4.97E-07	Capsicum annuum catalase (LOC107850312), mRNA >dbj|AB007190.1| Capsicum annuum mRNA for catalase, complete cds
Cluster-33503.18627	K14165	4.179	208.142	-5.6661	9.59E-12	1.09E-08	Human DNA sequence from clone RP11-71L7 on chromosome 13, complete sequence
Cluster-5607.2	K06883	16.355	0.000	6.4349	4.14E-05	0.0097476	PREDICTED: Capsicum annuum ADP-ribosylation factor 1 (LOC107840467), mRNA
Cluster-33503.71045	JUP	2418.830	7212.507	-1.5763	0.00024668	0.045047	PREDICTED: Erinaceus europaeus junction plakoglobin (JUP), transcript variant X4, mRNA
Cluster-33503.93114	JUNB	433.497	1798.840	-2.0541	1.10E-05	0.0030051	PREDICTED: Erinaceus europaeus jun B proto-oncogene (JUNB), mRNA
Cluster-33503.81719	JUNB	517.047	1888.724	-1.8697	1.32E-05	0.00354	PREDICTED: Erinaceus europaeus jun B proto-oncogene (JUNB), mRNA
Cluster-33503.99874	JUNB	969.661	3647.270	-1.9118	1.59E-05	0.0041681	PREDICTED: Erinaceus europaeus jun B proto-oncogene (JUNB), mRNA
Cluster-33503.125431	JPH	415.029	1811.464	-2.1267	7.59E-06	0.0021492	PREDICTED: Odobenus rosmarus divergens junctophilin 2 (JPH2), mRNA
Cluster-33503.37885	JPH	225.396	847.481	-1.9125	5.27E-05	0.012015	PREDICTED: Erinaceus europaeus junctophilin 2 (JPH2), partial mRNA

<table>
<tr><td>Cluster-33503.109256</td><td>ICAM3</td><td>699.790</td><td>183.629</td><td>1.9299</td><td>0.00016842</td><td>0.032597</td><td>PREDICTED: Erinaceus europaeus intercellular adhesion molecule 3 (ICAM3), mRNA</td></tr>
<tr><td>Cluster-33503.7663</td><td>hya</td><td>6.237</td><td>133.854</td><td>-4.4834</td><td>1.21E-07</td><td>5.05E-05</td><td>PREDICTED: Erinaceus europaeus hyaluronidase PH-20-like (LOC103121176), mRNA</td></tr>
<tr><td>Cluster-53432.0</td><td>htpG, HSP90A</td><td>0.000</td><td>32.107</td><td>-7.5197</td><td>2.28E-07</td><td>8.91E-05</td><td>PREDICTED: Gossypium raimondii heat shock cognate protein 80-like (LOC105790254), mRNA</td></tr>
<tr><td>Cluster-73598.0</td><td>htpG, HSP90A</td><td>26.226</td><td>0.799</td><td>5.2018</td><td>0.00012126</td><td>0.024591</td><td>PREDICTED: Capsicum annuum heat shock cognate protein 80 (LOC107878833), mRNA</td></tr>
<tr><td>Cluster-33503.85619</td><td>HSPB1</td><td>144.563</td><td>562.782</td><td>-1.9658</td><td>0.00011209</td><td>0.02299</td><td>PREDICTED: Erinaceus europaeus heat shock protein family B (small) member 1 (HSPB1), mRNA</td></tr>
<tr><td>Cluster-30085.1</td><td>HSPA1_8</td><td>86.521</td><td>0.000</td><td>8.8385</td><td>5.38E-11</td><td>4.88E-08</td><td>PREDICTED: Capsicum annuum heat shock cognate 70 kDa protein 2 (LOC107842953), mRNA</td></tr>
<tr><td>Cluster-4647.0</td><td>HSPA1_8</td><td>0.000</td><td>33.063</td><td>-7.563</td><td>4.19E-07</td><td>0.00015347</td><td>PREDICTED: Gossypium arboreum probable mediator of RNA polymerase II transcription subunit 37c (LOC108486107), mRNA</td></tr>
<tr><td>Cluster-30085.4</td><td>HSPA1_8</td><td>0.000</td><td>23.758</td><td>-7.0852</td><td>4.90E-06</td><td>0.0014483</td><td>Gossypium hirsutum heat shock cognate 70 kDa protein 2-like (LOC107898826), mRNA >gb|FJ415194.1| Gossypium hirsutum clone SpotW20 heat shock protein 70 mRNA, complete cds</td></tr>
<tr><td>Cluster-33503.6765</td><td>HSD17B3</td><td>17.272</td><td>100.576</td><td>-2.5711</td><td>8.80E-05</td><td>0.018742</td><td>PREDICTED: Erinaceus europaeus hydroxysteroid (17-beta) dehydrogenase 3 (HSD17B3), mRNA</td></tr>
<tr><td>Cluster-33503.6317</td><td>HOX_11</td><td>9.497</td><td>63.858</td><td>-2.7377</td><td>1.53E-05</td><td>0.0040355</td><td>PREDICTED: Ailuropoda melanoleuca homeobox C11 (HOXC11), mRNA</td></tr>
<tr><td>Cluster-5154.0</td><td>HMGB3</td><td>0.000</td><td>24.177</td><td>-7.1113</td><td>3.97E-06</td><td>0.0012108</td><td>PREDICTED: Gossypium hirsutum high mobility group B protein 3-like (LOC107922191), mRNA</td></tr>
<tr><td>Cluster-33503.113977</td><td>HK</td><td>24.237</td><td>140.684</td><td>-2.5097</td><td>3.69E-05</td><td>0.008812</td><td>PREDICTED: Erinaceus europaeus hexokinase 2 (HK2), mRNA</td></tr>
</table>

Cluster-33503.113974	HK	371.094	1174.919	-1.6614	0.00021369	0.039936	PREDICTED: Erinaceus europaeus hexokinase 2 (HK2), mRNA
Cluster-33503.117979	HCK	65.000	6.724	3.2352	0.00022564	0.041812	PREDICTED: Erinaceus europaeus HCK proto-oncogene, Src family tyrosine kinase (HCK), transcript variant X1, mRNA
Cluster-104772.0	HAO	45.695	0.000	7.9152	2.48E-08	1.19E-05	PREDICTED: Capsicum annuum peroxisomal (S)-2-hydroxy-acid oxidase GLO1 (LOC107852026), transcript variant X1, mRNA
Cluster-33503.70664	HAND2	60.443	3.615	4.1028	1.99E-06	0.00064634	PREDICTED: Odobenus rosmarus divergens heart and neural crest derivatives expressed 2 (HAND2), mRNA
Cluster-33503.70663	HAND2	44.392	1.890	4.495	4.27E-06	0.0012825	PREDICTED: Rousettus aegyptiacus heart and neural crest derivatives expressed 2 (HAND2), mRNA
Cluster-103956.0	H3	0.000	21.268	-6.927	1.74E-05	0.0044988	PREDICTED: Gossypium hirsutum histone H3.3 (LOC107950429), mRNA
Cluster-33503.34829	GRIA1	233.331	39.092	2.5741	4.77E-08	2.18E-05	PREDICTED: Erinaceus europaeus glutamate ionotropic receptor AMPA type subunit 1 (GRIA1), transcript variant X2, mRNA
Cluster-33503.32807	GPRC5D	36.322	436.951	-3.6056	1.17E-11	1.28E-08	PREDICTED: Erinaceus europaeus G protein-coupled receptor class C group 5 member D (GPRC5D), mRNA
Cluster-33503.32819	GPRC5D	1275.974	8366.040	-2.7135	4.78E-07	0.00017364	PREDICTED: Erinaceus europaeus G protein-coupled receptor class C group 5 member D (GPRC5D), mRNA
Cluster-33503.32820	GPRC5D	93.988	615.936	-2.7195	1.68E-06	0.00055482	PREDICTED: Erinaceus europaeus G protein-coupled receptor class C group 5 member D (GPRC5D), mRNA
Cluster-33503.32812	GPRC5D	35.121	251.823	-2.8573	1.90E-06	0.00062044	PREDICTED: Erinaceus europaeus G protein-coupled receptor class C group 5 member D (GPRC5D), mRNA
Cluster-33503.32808	GPRC5D	8.953	94.770	-3.4315	4.48E-06	0.0013364	PREDICTED: Erinaceus europaeus G protein-coupled receptor class C group 5 member D (GPRC5D), mRNA

Cluster-33503.32817	GPRC5D	689.590	4052.221	-2.5559	5.40E-06	0.0015784	PREDICTED: Erinaceus europaeus G protein-coupled receptor class C group 5 member D (GPRC5D), mRNA
Cluster-33503.32823	GPRC5D	10.174	61.407	-2.6246	0.00024363	0.04452	PREDICTED: Erinaceus europaeus G protein-coupled receptor class C group 5 member D (GPRC5D), mRNA
Cluster-103993.1	glyA, SHMT	12.816	0.000	6.0792	0.00023014	0.042455	PREDICTED: Nicotiana tabacum serine hydroxymethyltransferase, mitochondrial-like (LOC107785928), mRNA
Cluster-67057.0	GLT1	21.271	0.000	6.8133	7.16E-06	0.0020367	Capsicum annuum ferredoxin-dependent glutamate synthase, chloroplastic (LOC107852264), mRNA >gb|EU616563.1| Capsicum annuum putative ferredoxin-dependent glutamate synthase 1 mRNA, complete cds
Cluster-109336.1	glnA, GLUL	20.512	0.000	6.7566	1.01E-05	0.0027869	PREDICTED: Capsicum annuum glutamine synthetase, chloroplastic (LOC107841845), mRNA
Cluster-4133.0	glnA, GLUL	0.000	12.467	-6.1531	0.00026094	0.047332	PREDICTED: Gossypium arboreum glutamine synthetase nodule isozyme-like (LOC108460245), mRNA
Cluster-33503.154549	GLIS1_3	46.055	192.421	-2.0588	3.19E-05	0.0076716	PREDICTED: Erinaceus europaeus GLIS family zinc finger 1 (GLIS1), mRNA
Cluster-33503.124535	GJB6, CX30	773.662	2759.391	-1.834	4.22E-05	0.0099129	PREDICTED: Erinaceus europaeus gap junction beta-6 protein (LOC103119205), transcript variant X2, mRNA
Cluster-4061.0	GAPDH, gapA	0.000	33.773	-7.5917	2.13E-07	8.44E-05	PREDICTED: Gossypium hirsutum glyceraldehyde-3-phosphate dehydrogenase 2, cytosolic (LOC107899696), mRNA
Cluster-75673.0	GAPDH, gapA	15.873	0.000	6.3858	6.99E-05	0.015406	PREDICTED: Capsicum annuum glyceraldehyde-3-phosphate dehydrogenase, cytosolic (LOC107875869), mRNA

Cluster-31047.0	GAPDH, gapA	21.315	0.399	5.853	8.14E-05	0.017578	PREDICTED: Capsicum annuum glyceraldehyde-3-phosphate dehydrogenase A, chloroplastic-like (LOC107877699), mRNA
Cluster-23208.0	GABRR	0.760	27.830	-5.3956	6.27E-05	0.01395	PREDICTED: Erinaceus europaeus gamma-aminobutyric acid type A receptor rho3 subunit (GABRR3), mRNA
Cluster-33503.123342	FTL	0.000	15.333	-6.4655	9.54E-05	0.020087	Mus musculus ferritin light polypeptide 1 (Ftl1), mRNA
Cluster-33503.99991	FOXQ, HFH1	689.686	10504.793	-3.9291	2.14E-21	1.29E-17	PREDICTED: Erinaceus europaeus forkhead box Q1 (FOXQ1), mRNA
Cluster-33503.94260	FOXQ, HFH1	53.708	685.631	-3.662	1.56E-13	2.66E-10	PREDICTED: Erinaceus europaeus forkhead box Q1 (FOXQ1), mRNA
Cluster-33503.157596	FOXN	627.041	2935.275	-2.2263	6.59E-07	0.0002345	PREDICTED: Erinaceus europaeus forkhead box N1 (FOXN1), mRNA
Cluster-33503.64505	FLNA	2734.273	8720.566	-1.6733	2.67E-05	0.006549	PREDICTED: Erinaceus europaeus filamin C (FLNC), transcript variant X1, mRNA
Cluster-33503.48259	FLG	5270.681	1301.180	2.0182	0.00021161	0.039602	PREDICTED: Erinaceus europaeus hornerin (HRNR), mRNA
Cluster-33503.2404	FGF	1.120	38.689	-5.2526	8.42E-05	0.018049	PREDICTED: Erinaceus europaeus fibroblast growth factor 6 (FGF6), mRNA
Cluster-33503.98988	FCN	472.224	109.426	2.1123	7.48E-05	0.016294	PREDICTED: Erinaceus europaeus ficolin-1-like (LOC103112120), transcript variant X2, mRNA
Cluster-33503.49547	FBXO45	3.659	123.890	-5.1632	4.90E-08	2.24E-05	--
Cluster-33503.67520	EPS8	0.940	37.830	-5.3455	2.27E-06	0.00072687	PREDICTED: Erinaceus europaeus EPS8 like 3 (EPS8L3), mRNA
Cluster-33503.57563	EPHA6, EHK2	3.780	42.673	-3.4629	1.76E-05	0.0045387	PREDICTED: Ovis aries musimon EPH receptor A6 (EPHA6), transcript variant X1, mRNA

Cluster-33503.148482	ENTPD1_3_8	85.805	442.354	-2.362	4.22E-05	0.0099151	PREDICTED: Erinaceus europaeus ectonucleoside triphosphate diphosphohydrolase 3-like (LOC103118102), mRNA
Cluster-33503.56757	ENPEP	496.660	5131.535	-3.3692	2.35E-11	2.37E-08	PREDICTED: Erinaceus europaeus glutamyl aminopeptidase (ENPEP), mRNA
Cluster-4801.0	ENO, eno	0.000	18.396	-6.7144	2.91E-05	0.007106	PREDICTED: Gossypium arboreum enolase (LOC108467171), mRNA
Cluster-33503.142177	ENDOU, PP11	285.233	1565.438	-2.4566	2.17E-07	8.53E-05	PREDICTED: Erinaceus europaeus endonuclease, poly(U) specific (ENDOU), transcript variant X3, mRNA
Cluster-33503.134419	EN	39.882	944.554	-4.5851	8.16E-18	3.16E-14	PREDICTED: Rousettus aegyptiacus engrailed homeobox 1 (EN1), mRNA
Cluster-5396.6	EEF1A	1.780	29.299	-4.0148	0.00011006	0.022658	PREDICTED: Gossypium hirsutum elongation factor 1-alpha-like (LOC107905285), transcript variant X2, mRNA
Cluster-33503.133747	EDN3	142.547	35.049	2.0244	0.00025535	0.046473	PREDICTED: Erinaceus europaeus endothelin 3 (EDN3), mRNA
Cluster-33503.25761	EAF	58.744	382.611	-2.7144	2.26E-08	1.10E-05	PREDICTED: Erinaceus europaeus ELL associated factor 1 (EAF1), transcript variant X2, mRNA
Cluster-33503.7599	E4.2.1.1	39.602	501.506	-3.6478	3.44E-10	2.53E-07	PREDICTED: Erinaceus europaeus carbonic anhydrase 12 (CA12), transcript variant X2, mRNA
Cluster-33503.113116	E3.5.3.15	552.052	2004.559	-1.8598	5.43E-06	0.0015866	PREDICTED: Erinaceus europaeus peptidyl arginine deiminase 4 (PADI4), mRNA
Cluster-4248.1	E3.3.1.1, ahcY	0.400	29.908	-6.4525	7.42E-06	0.0021078	PREDICTED: Gossypium hirsutum adenosylhomocysteinase (LOC107890502), mRNA
Cluster-33503.67684	DSP	529.285	1714.888	-1.6961	9.00E-05	0.019091	PREDICTED: Erinaceus europaeus desmoplakin (DSP), transcript variant X3, mRNA

Cluster-33503.62375	DSP	2173.695	6455.861	-1.5704	0.0001763	0.033905	PREDICTED: Erinaceus europaeus desmoplakin (DSP), transcript variant X3, mRNA
Cluster-33503.139777	DSG3	1152.469	7333.451	-2.6697	5.11E-08	2.32E-05	PREDICTED: Erinaceus europaeus desmoglein 3 (DSG3), mRNA
Cluster-33503.51392	DSC2	793.606	11356.936	-3.8385	3.50E-13	5.55E-10	PREDICTED: Erinaceus europaeus desmocollin 2 (DSC2), mRNA
Cluster-33503.19981	DRD2	144.364	535.529	-1.8886	5.57E-05	0.01261	PREDICTED: Erinaceus europaeus dopamine receptor D2 (DRD2), transcript variant X2, mRNA
Cluster-96621.0	DNAJA2	50.682	0.000	8.0678	4.25E-09	2.42E-06	PREDICTED: Capsicum annuum dnaJ protein homolog (LOC107850595), mRNA
Cluster-33503.14519	DNAH	26.595	215.015	-2.9942	6.75E-06	0.0019397	PREDICTED: Erinaceus europaeus dynein axonemal heavy chain 7 (DNAH7), mRNA
Cluster-33503.134177	DMN	84.783	290.010	-1.7757	5.22E-05	0.011922	PREDICTED: Microcebus murinus synemin, intermediate filament protein (SYNM), transcript variant X4, mRNA
Cluster-33503.152972	dgkA, DGK	15.857	185.680	-3.5353	4.95E-12	6.05E-09	PREDICTED: Erinaceus europaeus diacylglycerol kinase gamma (DGKG), transcript variant X1, mRNA
Cluster-33503.66657	DES	908.658	6199.751	-2.7709	7.35E-11	6.52E-08	PREDICTED: Erinaceus europaeus desmin (DES), mRNA
Cluster-33503.121861	DES	199.555	1077.406	-2.4329	1.48E-09	9.33E-07	PREDICTED: Erinaceus europaeus desmin (DES), mRNA
Cluster-33503.66671	DES	48.270	345.810	-2.8446	2.66E-09	1.60E-06	PREDICTED: Erinaceus europaeus desmin (DES), mRNA
Cluster-33503.66676	DES	76.133	477.249	-2.6532	2.15E-08	1.05E-05	PREDICTED: Erinaceus europaeus desmin (DES), mRNA
Cluster-33503.66673	DES	667.894	3535.833	-2.4043	2.58E-08	1.23E-05	PREDICTED: Erinaceus europaeus desmin (DES), mRNA

Cluster-33503.49805	DES	12.797	81.481	-2.6703	3.32E-06	0.0010222	PREDICTED: Erinaceus europaeus desmin (DES), mRNA
Cluster-33503.66664	DES	0.360	26.479	-6.2766	1.71E-05	0.0044442	PREDICTED: Erinaceus europaeus desmin (DES), mRNA
Cluster-33503.66668	DES	11.559	74.841	-2.7261	2.24E-05	0.0055753	PREDICTED: Erinaceus europaeus desmin (DES), mRNA
Cluster-90162.1	DDX43	0.000	49.920	-8.156	4.38E-09	2.48E-06	PREDICTED: Erinaceus europaeus DEAD-box helicase 43 (DDX43), mRNA
Cluster-64767.0	DDX21	16.478	0.000	6.4456	3.77E-05	0.0089817	PREDICTED: Capsicum annuum DEAD-box ATP-dependent RNA helicase 7-like (LOC107853445), mRNA
Cluster-33503.96008	CXCL5_6, SCYB5_6	105.906	23.114	2.1996	9.06E-05	0.019185	PREDICTED: Erinaceus europaeus alveolar macrophage chemotactic factor (LOC103113953), partial mRNA
Cluster-3808.0	CTSL	0.000	38.713	-7.7882	6.10E-08	2.74E-05	PREDICTED: Gossypium hirsutum low-temperature-induced cysteine proteinase-like (LOC107900642), transcript variant X1, mRNA
Cluster-33503.27714	CTH	250.944	1616.742	-2.6882	1.25E-05	0.0033675	PREDICTED: Erinaceus europaeus cystathionine gamma-lyase (CTH), mRNA
Cluster-33503.39768	CSTA_B	219.404	2369.945	-3.4342	3.01E-06	0.00093631	PREDICTED: Erinaceus europaeus cystatin A (CSTA), mRNA
Cluster-33503.39767	CSTA_B	40.012	417.739	-3.3888	1.65E-05	0.0043284	PREDICTED: Erinaceus europaeus cystatin A (CSTA), mRNA
Cluster-33503.39770	CSTA_B	2.478	51.159	-4.376	2.16E-05	0.0054279	PREDICTED: Erinaceus europaeus cystatin A (CSTA), mRNA
Cluster-33503.39769	CSTA_B	22.932	207.656	-3.1823	5.82E-05	0.013108	PREDICTED: Erinaceus europaeus cystatin A (CSTA), mRNA

Cluster-33503.105498	CSF3R	56.296	10.928	2.3695	0.00022909	0.04229	PREDICTED: Erinaceus europaeus colony stimulating factor 3 receptor (CSF3R), transcript variant X2, mRNA
Cluster-33503.111757	CSF1R, FMS	570.671	176.759	1.6888	0.00012059	0.02451	PREDICTED: Erinaceus europaeus colony stimulating factor 1 receptor (CSF1R), mRNA
Cluster-34837.1	CRHR1	0.220	26.946	-6.3149	1.26E-05	0.0033761	PREDICTED: Erinaceus europaeus corticotropin releasing hormone receptor 1 (CRHR1), transcript variant X2, mRNA
Cluster-33503.3899	COX2	0.000	23.186	-7.0563	6.42E-06	0.001854	Mus musculus cytochrome c oxidase subunit II (Cox2) mRNA, complete cds; mitochondrial gene for mitochondrial product
Cluster-33503.182088	COX1	0.000	45.825	-8.0405	2.74E-08	1.31E-05	Mus musculus mitochondrial DNA, complete genome, clone: P29mtB6
Cluster-33503.81350	CLDN	98.183	629.996	-2.683	4.00E-07	0.00014807	PREDICTED: Erinaceus europaeus claudin 4 (CLDN4), mRNA
Cluster-33503.91528	CLDN	1091.014	5584.541	-2.3556	2.95E-06	0.00092119	PREDICTED: Erinaceus europaeus claudin 4 (CLDN4), mRNA
Cluster-33503.81352	CLDN	16.678	98.322	-2.575	0.00011336	0.023215	PREDICTED: Erinaceus europaeus claudin 4 (CLDN4), mRNA
Cluster-5714.0	CIRBP	0.000	13.685	-6.2889	0.00012368	0.025044	PREDICTED: Gossypium hirsutum glycine-rich RNA-binding protein GRP2A-like (LOC107928000), mRNA
Cluster-33503.179356	CHST1	7.201	50.191	-2.8382	0.0001687	0.032604	PREDICTED: Erinaceus europaeus carbohydrate sulfotransferase 1 (CHST1), mRNA
Cluster-111243.0	CHRNB4	18.302	175.871	-3.276	3.69E-09	2.13E-06	PREDICTED: Erinaceus europaeus cholinergic receptor nicotinic beta 4 subunit (CHRNB4), mRNA
Cluster-33503.110168	CFP	685.934	77.748	3.1414	2.00E-05	0.0050732	PREDICTED: Erinaceus europaeus complement factor properdin (CFP), mRNA

Cluster-33503.85134	CEACAM	48.133	222.600	-2.2012	6.35E-05	0.014105	PREDICTED: Erinaceus europaeus carcinoembryonic antigen-related cell adhesion molecule 1-like (LOC103122354), transcript variant X3, mRNA
Cluster-33503.171379	CD8A	18.868	0.000	6.6371	0.00015503	0.030382	PREDICTED: Erinaceus europaeus CD8a molecule (CD8A), mRNA
Cluster-33503.29821	CD84	282.446	80.336	1.8167	0.00020073	0.037958	PREDICTED: Erinaceus europaeus CD84 molecule (CD84), mRNA
Cluster-33503.105730	CD6	183.282	14.207	3.6891	2.24E-05	0.0055766	PREDICTED: Erinaceus europaeus CD6 molecule (CD6), mRNA
Cluster-33503.8700	CD5	88.587	8.873	3.3013	0.0001553	0.030404	PREDICTED: Erinaceus europaeus CD5 molecule (CD5), mRNA
Cluster-33503.11044	CD38	60.295	7.431	3.0322	0.00021461	0.040053	PREDICTED: Erinaceus europaeus ADP-ribosyl cyclase/cyclic ADP-ribose hydrolase 1-like (LOC107522258), mRNA
Cluster-33503.27904	CD209	1037.825	259.717	1.9987	1.93E-05	0.0049274	PREDICTED: Erinaceus europaeus CD209 antigen-like protein 2 (LOC103122253), mRNA
Cluster-33503.123057	CCR7	60.494	2.684	4.4866	3.15E-05	0.0076164	PREDICTED: Erinaceus europaeus C-C motif chemokine receptor 7 (CCR7), mRNA
Cluster-33503.37515	CCL5	71.856	7.351	3.2826	0.0002027	0.038249	PREDICTED: Erinaceus europaeus C-C motif chemokine ligand 5 (CCL5), mRNA
Cluster-33503.151316	CCL28	30.887	0.799	5.4423	8.42E-05	0.018049	PREDICTED: Erinaceus europaeus C-C motif chemokine ligand 28 (CCL28), mRNA
Cluster-33503.49633	CARD11	123.105	13.491	3.1876	0.00021619	0.040262	PREDICTED: Erinaceus europaeus caspase recruitment domain family member 11 (CARD11), mRNA
Cluster-33503.2135	CALR	0.000	14.024	-6.3241	9.65E-05	0.020258	PREDICTED: Gossypium hirsutum calreticulin-like (LOC107888331), mRNA

Cluster-33503.151610	CACNA1I	59.972	221.486	-1.8872	9.36E-05	0.01979	PREDICTED: Erinaceus europaeus calcium voltage-gated channel subunit alpha1 I (CACNA1I), partial mRNA
Cluster-33503.132221	CACNA1H	289.221	2515.022	-3.1193	6.21E-09	3.39E-06	PREDICTED: Ceratotherium simum simum calcium channel, voltage-dependent, T type, alpha 1H subunit (LOC101397703), mRNA
Cluster-33503.156811	CA2	88.463	886.811	-3.3281	2.11E-08	1.03E-05	PREDICTED: Erinaceus europaeus carbonic anhydrase 2 (CA2), mRNA
Cluster-33503.57851	C4	935.135	175.993	2.4092	1.83E-07	7.31E-05	PREDICTED: Erinaceus europaeus complement C4-A-like (LOC103117357), transcript variant X1, mRNA
Cluster-33503.77992	C3	1771.853	350.391	2.3391	1.80E-07	7.20E-05	PREDICTED: Equus caballus complement C3-like (LOC100060539), mRNA
Cluster-33503.47304	C1QG	3511.821	752.464	2.2227	0.00010261	0.0214	PREDICTED: Erinaceus europaeus complement component 1, q subcomponent, C chain (C1QC), mRNA
Cluster-33503.73583	C1QA	7078.985	1470.947	2.2669	3.65E-05	0.0087065	PREDICTED: Erinaceus europaeus complement component 1, q subcomponent, A chain (C1QA), mRNA
Cluster-33503.140486	BTK	182.186	42.349	2.1111	0.00020895	0.039238	PREDICTED: Erinaceus europaeus Bruton tyrosine kinase (BTK), transcript variant X1, mRNA
Cluster-33503.148918	BMP8	47.395	202.359	-2.0914	9.52E-06	0.0026326	PREDICTED: Erinaceus europaeus bone morphogenetic protein 8A (LOC103117103), mRNA
Cluster-113923.0	BHMT	5.480	60.987	-3.4697	6.68E-06	0.001922	PREDICTED: Erinaceus europaeus betaine--homocysteine S-methyltransferase 1 (LOC103109924), mRNA
Cluster-33503.162563	B4GALNT2	17.927	0.000	6.5707	5.69E-05	0.012844	PREDICTED: Erinaceus europaeus beta-1,4-N-acetyl-galactosaminyltransferase 2 (B4GALNT2), mRNA

Cluster-33503.105377	ATPeV1B, ATP6B	222.875	30.137	2.8843	2.72E-06	0.0008534	PREDICTED: Erinaceus europaeus ATPase H+ transporting V1 subunit B1 (ATP6V1B1), mRNA
Cluster-22105.0	ATPeF1B, ATP5B, ATP2	0.000	15.217	-6.4412	6.25E-05	0.013913	PREDICTED: Gossypium hirsutum ATP synthase subunit beta, mitochondrial-like (LOC107899003), mRNA
Cluster-33503.110399	ATF3, LRF1	190.728	1014.950	-2.4104	0.00010296	0.021456	PREDICTED: Erinaceus europaeus activating transcription factor 3 (ATF3), mRNA
Cluster-33503.51219	argG, ASS1	974.164	4216.615	-2.1142	2.09E-05	0.0052668	PREDICTED: Erinaceus europaeus argininosuccinate synthase 1 (ASS1), mRNA
Cluster-33503.56207	AQP5	2025.176	112.143	4.1758	2.50E-09	1.51E-06	PREDICTED: Erinaceus europaeus aquaporin 5 (AQP5), mRNA
Cluster-29855.0	AQP0, MIP	13.879	0.000	6.1926	0.00015704	0.030634	Capsicum annuum probable aquaporin TIP1-1 (LOC107875613), mRNA
Cluster-33503.142390	AOX	159.632	18.532	3.0907	1.26E-05	0.0033761	PREDICTED: Erinaceus europaeus aldehyde oxidase 4-like (LOC103121600), mRNA
Cluster-4854.0	ANXA3	0.000	13.027	-6.2149	0.00020614	0.038845	PREDICTED: Gossypium raimondii annexin D2-like (LOC105804217), mRNA
Cluster-43933.0	ANXA3	0.000	12.721	-6.1804	0.00026484	0.047848	PREDICTED: Gossypium raimondii annexin D1 (LOC105769557), mRNA
Cluster-4873.0	ANXA13	0.000	15.404	-6.458	6.74E-05	0.014912	Gossypium hirsutum clone Spot24 annexin mRNA, complete cds
Cluster-33503.70618	ALOX15	1014.805	59.643	4.0947	2.46E-09	1.49E-06	PREDICTED: Erinaceus europaeus arachidonate 15-lipoxygenase (ALOX15), transcript variant X2, mRNA
Cluster-70254.1	ALDO	14.374	0.000	6.2416	0.00013333	0.026642	PREDICTED: Capsicum annuum fructose-bisphosphate aldolase 1, chloroplastic-like (LOC107858551), mRNA

Cluster-33503.186772	AIP, XAP2	0.220	21.342	-5.9674	9.08E-05	0.019209	PREDICTED: Erinaceus europaeus aryl hydrocarbon receptor interacting protein like 1 (AIPL1), transcript variant X1, mRNA
Cluster-33503.28069	ADRA2B	41.188	484.054	-3.5675	6.97E-12	8.19E-09	PREDICTED: Erinaceus europaeus adrenoceptor alpha 2B (ADRA2B), mRNA
Cluster-33503.19140	ADRA1D	143.689	860.997	-2.5855	2.40E-05	0.0059401	PREDICTED: Erinaceus europaeus adrenoceptor alpha 1D (ADRA1D), mRNA
Cluster-33503.142927	ADCY8	27.671	0.672	5.3594	0.00011562	0.023642	PREDICTED: Erinaceus europaeus adenylate cyclase 8 (brain) (ADCY8), mRNA
Cluster-33503.95323	ACTG2	3.778	54.960	-3.9331	1.22E-06	0.00041609	PREDICTED: Erinaceus europaeus actin, gamma 2, smooth muscle, enteric (ACTG2), transcript variant X2, mRNA
Cluster-33503.83648	ACTG2	2036.325	15759.823	-2.9525	2.47E-05	0.006089	PREDICTED: Erinaceus europaeus actin, gamma 2, smooth muscle, enteric (ACTG2), transcript variant X1, mRNA
Cluster-33503.179118	ACTG2	2231.461	17427.208	-2.9656	2.73E-05	0.0066944	PREDICTED: Erinaceus europaeus actin, gamma 2, smooth muscle, enteric (ACTG2), transcript variant X3, mRNA
Cluster-33503.84249	ACTG2	1014.199	7507.258	-2.8886	3.16E-05	0.0076229	PREDICTED: Erinaceus europaeus actin, gamma 2, smooth muscle, enteric (ACTG2), transcript variant X2, mRNA
Cluster-33503.78543	ACTC1	0.000	20.163	-6.8551	2.52E-05	0.0062206	PREDICTED: Erinaceus europaeus actin, gamma 2, smooth muscle, enteric (ACTG2), transcript variant X3, mRNA
Cluster-33503.22638	ACTB_G1	475.049	41.113	3.5167	2.18E-09	1.34E-06	PREDICTED: Erinaceus europaeus actin, beta-like 2 (ACTBL2), transcript variant X1, mRNA
Cluster-33503.37	ACTB_G1	0.000	20.286	-6.8535	1.42E-05	0.0037605	PREDICTED: Gossypium hirsutum actin-7-like (LOC107899518), mRNA

Cluster-33503.39	ACTB_G1	0.000	13.087	-6.2215	0.00022774	0.042097	PREDICTED: Gossypium raimondii actin-58 (LOC105779793), mRNA
Cluster-33503.30640	ACTA2	38.917	397.646	-3.3691	4.92E-06	0.0014534	PREDICTED: Erinaceus europaeus actin, gamma 2, smooth muscle, enteric (ACTG2), transcript variant X2, mRNA
Cluster-33503.84557	ACTA2	27.868	184.124	-2.7441	0.00027077	0.048821	PREDICTED: Erinaceus europaeus actin, gamma 2, smooth muscle, enteric (ACTG2), transcript variant X2, mRNA
Cluster-115913.0	ABCF2	20.393	0.000	6.7536	8.35E-06	0.0023269	PREDICTED: Capsicum annuum ABC transporter F family member 1 (LOC107847440), mRNA
Cluster-33503.122885	--	195.856	14481.972	-6.209	2.77E-38	7.53E-33	Ovis canadensis canadensis isolate 43U chromosome 4 sequence
Cluster-33503.122883	--	101.285	7082.944	-6.1331	6.90E-37	9.37E-32	--
Cluster-33503.72557	--	144.633	10901.455	-6.2405	3.42E-34	3.10E-29	--
Cluster-33503.121109	--	98.966	7951.453	-6.3263	3.78E-33	2.56E-28	PREDICTED: Erinaceus europaeus keratin, type II cytoskeletal 6A (LOC103109181), mRNA
Cluster-33503.72558	--	51.429	4488.437	-6.4418	5.68E-32	3.08E-27	--
Cluster-33503.122884	--	226.015	13263.055	-5.8757	3.03E-31	1.29E-26	--
Cluster-33503.122882	--	77.182	4752.188	-5.9464	6.32E-30	1.91E-25	--
Cluster-33503.130322	--	1465.798	44858.219	-4.9359	1.10E-27	2.14E-23	PREDICTED: Erinaceus europaeus keratin, type II cytoskeletal 75-like (LOC103109183), mRNA
Cluster-33503.185133	--	0.800	1274.284	-10.47	1.17E-26	1.77E-22	PREDICTED: Erinaceus europaeus S100 calcium binding protein A12 (S100A12), mRNA
Cluster-33503.138572	--	116.146	2731.749	-4.5585	1.96E-26	2.80E-22	PREDICTED: Erinaceus europaeus leiomodin 1 (LMOD1), mRNA
Cluster-33503.101002	--	66.969	3997.177	-5.9096	1.08E-25	1.40E-21	PREDICTED: Erinaceus europaeus keratin, type II cytoskeletal 6A (LOC103109181), mRNA

Cluster-33503.66658	--	39.635	1018.414	-4.6685	9.80E-25	1.11E-20	Atelerix albiventris clone LB4-495B20, complete sequence
Cluster-33503.57698	--	16.091	1291.022	-6.3315	6.22E-24	6.49E-20	PREDICTED: Canis lupus familiaris keratin, type I cytoskeletal 16 (KRT16), mRNA
Cluster-33503.136024	--	48.036	1041.530	-4.4403	1.07E-22	9.04E-19	PREDICTED: Erinaceus europaeus leiomodin 1 (LMOD1), mRNA
Cluster-33503.69005	--	15.911	1076.808	-6.113	1.19E-22	9.82E-19	PREDICTED: Erinaceus europaeus keratin, type II cytoskeletal 6A (LOC103109181), mRNA
Cluster-33503.37076	--	2.478	340.305	-7.1195	1.32E-22	1.06E-18	PREDICTED: Dasypus novemcinctus Zic family member 1 (ZIC1), mRNA
Cluster-33503.37928	--	8.634	656.145	-6.2559	1.76E-22	1.33E-18	--
Cluster-33503.68995	--	46.958	2795.878	-5.9093	2.24E-22	1.64E-18	PREDICTED: Erinaceus europaeus keratin, type II cytoskeletal 6A-like (LOC103109273), mRNA
Cluster-33503.57692	--	8.435	887.849	-6.7573	2.48E-22	1.77E-18	--
Cluster-33503.184332	--	4.799	310.796	-6.0041	4.41E-22	2.99E-18	--
Cluster-33503.107372	--	23.265	1353.758	-5.8848	6.66E-22	4.41E-18	PREDICTED: Erinaceus europaeus keratin, type II cytoskeletal 6A (LOC103109181), mRNA
Cluster-33503.47157	--	283.869	9878.673	-5.1236	1.11E-21	6.83E-18	PREDICTED: Erinaceus europaeus keratin, type II cytoskeletal 75-like (LOC103109183), mRNA
Cluster-33503.123408	--	64.318	2879.507	-5.4889	3.48E-21	2.01E-17	PREDICTED: Erinaceus europaeus keratin, type II cytoskeletal 75-like (LOC103109183), mRNA
Cluster-33503.81354	--	337.898	3.196	6.6771	6.15E-20	3.27E-16	PREDICTED: Erinaceus europaeus family with sequence similarity 214 member A (FAM214A), mRNA
Cluster-33503.68988	--	59.651	2101.921	-5.1429	1.26E-19	6.24E-16	PREDICTED: Erinaceus europaeus keratin, type II cytoskeletal 6A (LOC103109181), mRNA
Cluster-33503.180521	--	5.198	313.376	-5.9531	2.06E-19	1.00E-15	--

Cluster-33503.14743	--	7.520	238.213	-4.9752	2.24E-19	1.07E-15	Atelerix albiventris clone LB4-112D1, complete sequence
Cluster-33503.68979	--	7.600	463.033	-5.8909	4.17E-19	1.89E-15	PREDICTED: Erinaceus europaeus keratin, type II cytoskeletal 6A (LOC103109181), mRNA
Cluster-33503.130115	--	1.818	418.987	-8.0104	4.65E-19	2.07E-15	PREDICTED: Erinaceus europaeus keratin-associated protein 13-1-like (LOC103118354), mRNA
Cluster-33503.44592	--	4.337	474.360	-6.786	1.14E-18	4.82E-15	PREDICTED: Erinaceus europaeus keratin-associated protein 19-3-like (LOC103118358), mRNA
Cluster-33503.8465	--	333.259	8766.853	-4.7185	3.35E-18	1.34E-14	PREDICTED: Erinaceus europaeus keratin-associated protein 13-2 (LOC103118343), mRNA
Cluster-33503.22812	--	183.603	5492.330	-4.9071	4.58E-18	1.80E-14	PREDICTED: Erinaceus europaeus keratin, type II cytoskeletal 75-like (LOC103109183), mRNA
Cluster-33503.48043	--	21.792	580.618	-4.7543	1.03E-17	3.95E-14	PREDICTED: Erinaceus europaeus keratin, type II cytoskeletal 75-like (LOC103109183), mRNA
Cluster-33503.179983	--	21.573	622.013	-4.8815	1.33E-17	5.02E-14	PREDICTED: Erinaceus europaeus keratin, type II cytoskeletal 75-like (LOC103109183), mRNA
Cluster-33503.68996	--	4.541	295.996	-5.978	2.53E-17	9.43E-14	PREDICTED: Erinaceus europaeus keratin, type II cytoskeletal 6A-like (LOC103109273), mRNA
Cluster-33503.126753	--	124.374	2175.052	-4.1346	2.73E-17	9.89E-14	PREDICTED: Erinaceus europaeus keratin-associated protein 11-1 (LOC103123006), mRNA
Cluster-33503.2120	--	2.438	246.471	-6.7753	2.71E-17	9.89E-14	PREDICTED: Erinaceus europaeus keratin 9 (KRT9), mRNA
Cluster-33503.123435	--	18.132	530.513	-4.8912	3.60E-17	1.27E-13	PREDICTED: Erinaceus europaeus keratin, type II cytoskeletal 75-like (LOC103109183), mRNA
Cluster-33503.16177	--	4.520	277.438	-5.8539	3.81E-17	1.33E-13	PREDICTED: Erinaceus europaeus keratin, type II cytoskeletal 75-like (LOC103109183), mRNA
Cluster-33503.132660	--	15.751	483.311	-4.9843	5.33E-17	1.83E-13	PREDICTED: Erinaceus europaeus calponin 1 (CNN1), transcript variant X1, mRNA

Cluster-33503.180338	--	1.199	323.809	-8.3716	1.06E-16	3.51E-13	PREDICTED: Erinaceus europaeus keratin, type II cytoskeletal 6A-like (LOC103109273), mRNA
Cluster-33503.138327	--	9.554	312.668	-5.0954	1.56E-16	4.97E-13	PREDICTED: Erinaceus europaeus calponin 1 (CNN1), transcript variant X1, mRNA
Cluster-33503.28433	--	1031.392	79.813	3.6914	2.57E-16	7.94E-13	--
Cluster-33503.44595	--	2.478	299.095	-6.9303	2.75E-16	8.39E-13	PREDICTED: Erinaceus europaeus keratin-associated protein 19-2-like (LOC103118359), mRNA
Cluster-33503.145439	--	22.552	551.657	-4.6305	3.24E-16	9.68E-13	PREDICTED: Erinaceus europaeus keratin, type II cytoskeletal 75-like (LOC103109183), mRNA
Cluster-33503.68994	--	23.492	817.696	-5.1462	3.52E-16	1.04E-12	PREDICTED: Erinaceus europaeus keratin, type II cytoskeletal 6A-like (LOC103109273), mRNA
Cluster-33503.16085	--	8.958	308.740	-5.069	3.58E-16	1.05E-12	PREDICTED: Erinaceus europaeus solute carrier family 13 member 5 (SLC13A5), transcript variant X1, mRNA
Cluster-33503.184982	--	6.598	389.578	-5.9356	5.17E-16	1.49E-12	--
Cluster-33503.46338	--	192.914	5583.933	-4.8589	8.08E-16	2.31E-12	PREDICTED: Erinaceus europaeus keratin, type II cytoskeletal 75-like (LOC103109183), mRNA
Cluster-33503.126674	--	1.239	255.244	-7.7076	1.02E-15	2.86E-12	PREDICTED: Erinaceus europaeus prostate stem cell antigen (PSCA), mRNA
Cluster-33503.8214	--	39.953	964.311	-4.6107	1.07E-15	2.96E-12	--
Cluster-33503.14744	--	11.493	328.948	-4.8673	1.15E-15	3.13E-12	Atelerix albiventris clone LB4-307O5, complete sequence
Cluster-33503.49624	--	101.121	1722.332	-4.0883	2.89E-15	7.41E-12	--
Cluster-33503.68985	--	4.678	247.550	-5.817	3.30E-15	8.36E-12	PREDICTED: Erinaceus europaeus keratin, type II cytoskeletal 6A-like (LOC103109273), mRNA
Cluster-33503.49300	--	45.483	690.688	-3.9396	3.41E-15	8.58E-12	--
Cluster-33503.125839	--	8.197	283.242	-5.1738	5.53E-15	1.37E-11	PREDICTED: Erinaceus europaeus keratin-associated protein 15-1 (LOC103118357), mRNA

Cluster-33503.178547	--	35.163	573.944	-4.0399	6.42E-15	1.56E-11	PREDICTED: Erinaceus europaeus keratin-associated protein 11-1 (LOC103123006), mRNA
Cluster-33503.132628	--	53.534	1359.029	-4.6804	6.92E-15	1.66E-11	PREDICTED: Gorilla gorilla gorilla calponin 1 (CNN1), transcript variant X2, mRNA
Cluster-33503.132653	--	8.257	290.538	-5.2175	8.43E-15	2.01E-11	PREDICTED: Erinaceus europaeus calponin 1 (CNN1), transcript variant X1, mRNA
Cluster-33503.44729	--	6.717	254.091	-5.2856	8.61E-15	2.03E-11	--
Cluster-33503.49872	--	67.970	1044.934	-3.9523	8.89E-15	2.08E-11	--
Cluster-33503.124634	--	383.194	21.271	4.1739	9.84E-15	2.28E-11	--
Cluster-33503.183159	--	3.897	228.033	-5.9129	1.15E-14	2.64E-11	Erinaceus europaeus, clone XX-29460857F24, complete sequence
Cluster-33503.37525	--	956.970	14074.657	-3.8782	1.26E-14	2.89E-11	PREDICTED: Erinaceus europaeus sperm mitochondria associated cysteine rich protein (SMCP), mRNA
Cluster-33503.122774	--	161.011	2762.302	-4.1041	1.81E-14	4.03E-11	--
Cluster-33503.127687	--	209.789	2.730	6.3776	1.85E-14	4.09E-11	Atelerix albiventris clone LB4-443C20, complete sequence
Cluster-33503.145218	--	724.079	27.354	4.7422	1.90E-14	4.16E-11	PREDICTED: Erinaceus europaeus lysozyme g2 (LYG2), mRNA
Cluster-33503.126746	--	729.643	10331.006	-3.8247	2.07E-14	4.49E-11	PREDICTED: Erinaceus europaeus keratin-associated protein 11-1 (LOC103123006), mRNA
Cluster-30085.0	--	189.521	0.000	9.9719	2.51E-14	5.37E-11	Capsicum annuum chitin binding protein mRNA, complete cds
Cluster-33503.123258	--	79.950	1115.868	-3.8119	2.84E-14	6.02E-11	--
Cluster-33503.43522	--	213.432	5851.676	-4.78	3.37E-14	7.04E-11	PREDICTED: Erinaceus europaeus keratin-associated protein 15-1 (LOC103118357), mRNA
Cluster-33503.125843	--	764.929	18575.231	-4.6026	3.62E-14	7.51E-11	--

Cluster-33503.8185	--	550.401	8020.930	-3.8665	4.03E-14	8.28E-11	PREDICTED: Erinaceus europaeus keratin-associated protein 11-1 (LOC103123006), mRNA
Cluster-33503.126745	--	141.654	2272.069	-4.0092	4.90E-14	9.85E-11	PREDICTED: Erinaceus europaeus keratin-associated protein 11-1 (LOC103123006), mRNA
Cluster-33503.72568	--	1.780	127.457	-6.1313	5.18E-14	1.04E-10	PREDICTED: Erinaceus europaeus keratin, type II cytoskeletal 6A (LOC103109181), mRNA
Cluster-33503.169198	--	2.478	199.818	-6.3479	5.31E-14	1.05E-10	PREDICTED: Erinaceus europaeus Rh family C glycoprotein (RHCG), transcript variant X2, mRNA
Cluster-33503.126744	--	211.313	3000.705	-3.831	5.72E-14	1.12E-10	PREDICTED: Erinaceus europaeus keratin-associated protein 11-1 (LOC103123006), mRNA
Cluster-33503.47642	--	221.623	2998.582	-3.7589	6.90E-14	1.33E-10	--
Cluster-33503.148266	--	545.600	7773.203	-3.8341	7.08E-14	1.34E-10	PREDICTED: Erinaceus europaeus keratin-associated protein 11-1 (LOC103123006), mRNA
Cluster-33503.39188	--	183.174	2.604	6.215	7.36E-14	1.39E-10	Atelerix albiventris clone LB4-244I20, complete sequence
Cluster-33503.126743	--	700.041	10425.119	-3.8976	7.48E-14	1.39E-10	PREDICTED: Erinaceus europaeus keratin-associated protein 11-1 (LOC103123006), mRNA
Cluster-33503.132626	--	19.387	517.818	-4.7759	7.47E-14	1.39E-10	PREDICTED: Erinaceus europaeus calponin 1 (CNN1), transcript variant X1, mRNA
Cluster-33503.180546	--	155.552	2166.187	-3.8046	7.71E-14	1.43E-10	PREDICTED: Erinaceus europaeus keratin-associated protein 11-1 (LOC103123006), mRNA
Cluster-33503.49795	--	58.147	562.394	-3.2711	8.43E-14	1.54E-10	Homo sapiens partial IRAG gene, alternative exons 1a, 1b, 1c, 1d, 6, 9a and 21
Cluster-33503.7038	--	37.053	434.604	-3.5479	8.93E-14	1.62E-10	PREDICTED: Erinaceus europaeus myocardin (MYOCD), mRNA
Cluster-33503.180370	--	49.871	1107.357	-4.4877	1.27E-13	2.25E-10	PREDICTED: Erinaceus europaeus calponin 1 (CNN1), transcript variant X2, mRNA

Cluster-33503.164399	--	36.786	579.909	-3.9992	1.28E-13	2.26E-10	PREDICTED: Erinaceus europaeus keratin-associated protein 11-1 (LOC103123006), mRNA
Cluster-33503.125462	--	1693.801	21034.886	-3.6348	1.33E-13	2.32E-10	PREDICTED: Erinaceus europaeus keratin-associated protein 3-1 (LOC103120619), mRNA
Cluster-33503.134570	--	9.998	224.579	-4.5491	1.34E-13	2.34E-10	PREDICTED: Erinaceus europaeus calponin 1 (CNN1), transcript variant X2, mRNA
Cluster-33503.69762	--	6.239	305.368	-5.6405	1.39E-13	2.41E-10	Atelerix albiventris clone LB4-482N5, complete sequence
Cluster-33503.132643	--	18.773	467.488	-4.676	1.45E-13	2.49E-10	PREDICTED: Erinaceus europaeus calponin 1 (CNN1), transcript variant X2, mRNA
Cluster-33503.129745	--	1.199	1892.443	-10.781	1.71E-13	2.90E-10	PREDICTED: Erinaceus europaeus keratin-associated protein 13-1-like (LOC103118355), mRNA
Cluster-33503.132672	--	5.895	208.053	-5.2099	1.97E-13	3.30E-10	PREDICTED: Erinaceus europaeus calponin 1 (CNN1), transcript variant X2, mRNA
Cluster-33503.49410	--	419.775	26.391	3.9902	2.04E-13	3.40E-10	--
Cluster-33503.157809	--	10.456	200.025	-4.2988	3.06E-13	4.98E-10	--
Cluster-33503.44576	--	5.136	267.184	-5.7284	3.15E-13	5.10E-10	PREDICTED: Erinaceus europaeus keratin-associated protein 19-3-like (LOC103118358), mRNA
Cluster-33503.126748	--	3.859	113.468	-4.8586	3.30E-13	5.28E-10	PREDICTED: Erinaceus europaeus keratin-associated protein 11-1 (LOC103123006), mRNA
Cluster-33503.42516	--	25.504	568.112	-4.4999	3.54E-13	5.59E-10	PREDICTED: Erinaceus europaeus keratin-associated protein 15-1 (LOC103118357), mRNA
Cluster-33503.49440	--	51.258	691.488	-3.7635	3.74E-13	5.87E-10	PREDICTED: Erinaceus europaeus keratin, type II cuticular Hb6 (LOC103109202), mRNA
Cluster-33503.46935	--	490.800	5424.023	-3.4668	4.38E-13	6.83E-10	--
Cluster-33503.25477	--	1.679	194.966	-6.7893	4.51E-13	7.00E-10	PREDICTED: Erinaceus europaeus serine protease inhibitor Kazal-type 6-like (LOC103107621), mRNA

Cluster-33503.125841	--	346.626	7905.442	-4.5132	6.00E-13	9.00E-10	PREDICTED: Erinaceus europaeus keratin-associated protein 15-1 (LOC103118357), mRNA
Cluster-33503.123478	--	234.363	11.175	4.4014	6.26E-13	9.30E-10	PREDICTED: Capra hircus antifreeze protein Maxi-like (LOC108638522), mRNA
Cluster-33503.41747	--	119.966	2387.876	-4.3201	6.27E-13	9.30E-10	PREDICTED: Erinaceus europaeus keratin-associated protein 15-1 (LOC103118357), mRNA
Cluster-33503.179376	--	389.816	8007.578	-4.3619	6.41E-13	9.45E-10	--
Cluster-33503.126600	--	301.373	5449.203	-4.1782	6.67E-13	9.73E-10	PREDICTED: Erinaceus europaeus keratin-associated protein 15-1 (LOC103118357), mRNA
Cluster-33503.132633	--	7.836	202.999	-4.7606	7.70E-13	1.11E-09	PREDICTED: Erinaceus europaeus calponin 1 (CNN1), transcript variant X2, mRNA
Cluster-33503.68983	--	3.017	180.932	-6.0493	8.73E-13	1.25E-09	PREDICTED: Erinaceus europaeus keratin, type II cytoskeletal 6A-like (LOC103109273), mRNA
Cluster-33503.126359	--	551.249	13649.863	-4.6311	9.08E-13	1.29E-09	PREDICTED: Erinaceus europaeus keratin-associated protein 15-1 (LOC103118357), mRNA
Cluster-33503.127568	--	3.158	134.796	-5.5162	1.02E-12	1.45E-09	Atelerix albiventris clone LB4-197A24, complete sequence
Cluster-33503.34787	--	19.509	511.821	-4.75	1.22E-12	1.70E-09	--
Cluster-33503.132641	--	63.301	1862.527	-4.89	1.29E-12	1.77E-09	PREDICTED: Erinaceus europaeus calponin 1 (CNN1), transcript variant X2, mRNA
Cluster-33503.33973	--	135.854	0.000	9.4891	1.33E-12	1.82E-09	PREDICTED: Capsicum annuum ribulose bisphosphate carboxylase small chain 8B, chloroplastic (LOC107858580), mRNA
Cluster-33503.37530	--	25.371	580.863	-4.5028	1.38E-12	1.87E-09	PREDICTED: Erinaceus europaeus sperm mitochondria associated cysteine rich protein (SMCP), mRNA
Cluster-33503.44589	--	0.799	238.585	-8.5271	1.44E-12	1.94E-09	PREDICTED: Erinaceus europaeus keratin-associated protein 19-3-like (LOC103118358), mRNA

Cluster-33503.127101	--	73.820	1614.301	-4.4583	1.46E-12	1.96E-09	--
Cluster-33503.45910	--	9.578	249.271	-4.7277	1.47E-12	1.96E-09	PREDICTED: Erinaceus europaeus cornifin (LOC107523341), mRNA
Cluster-33503.184680	--	108.395	3038.825	-4.8113	1.77E-12	2.33E-09	PREDICTED: Erinaceus europaeus beta-defensin 103-like (LOC107522224), mRNA
Cluster-33503.49068	--	128.752	1655.141	-3.6895	1.86E-12	2.44E-09	PREDICTED: Erinaceus europaeus keratin, type II cuticular Hb6 (LOC103109202), mRNA
Cluster-33503.123607	--	2.799	109.988	-5.3102	1.98E-12	2.58E-09	PREDICTED: Erinaceus europaeus keratin, type II cytoskeletal 75-like (LOC103109183), mRNA
Cluster-33503.132636	--	14.933	378.921	-4.7107	2.04E-12	2.66E-09	PREDICTED: Erinaceus europaeus calponin 1 (CNN1), transcript variant X2, mRNA
Cluster-30607.0	--	214.303	0.399	9.1861	2.41E-12	3.08E-09	PREDICTED: Capsicum annuum glycine-rich protein DC7.1-like (LOC107864362), transcript variant X2, mRNA
Cluster-33503.126752	--	32.561	495.138	-3.9444	3.20E-12	4.03E-09	PREDICTED: Erinaceus europaeus keratin-associated protein 11-1 (LOC103123006), mRNA
Cluster-33503.49806	--	1570.784	104.252	3.9146	4.09E-12	5.08E-09	--
Cluster-33503.50148	--	917.592	12699.877	-3.7915	4.67E-12	5.77E-09	--
Cluster-33503.106414	--	4.118	123.995	-4.8915	4.77E-12	5.87E-09	Atelerix albiventris clone LB4-419B9, complete sequence
Cluster-33503.79757	--	19.128	458.695	-4.5849	4.98E-12	6.06E-09	Atelerix albiventris clone LB4-112D1, complete sequence
Cluster-33503.97490	--	3.780	126.206	-5.026	5.28E-12	6.40E-09	Homo sapiens actin, gamma 2, smooth muscle, enteric (ACTG2), RefSeqGene on chromosome 2
Cluster-33503.8840	--	50.789	548.910	-3.4447	5.45E-12	6.58E-09	--
Cluster-33503.123102	--	142.618	2312.493	-4.0244	5.96E-12	7.16E-09	PREDICTED: Erinaceus europaeus keratin, type II cuticular Hb6 (LOC103109202), mRNA

Cluster-33503.57701	--	152.141	2459.355	-4.0186	6.01E-12	7.20E-09	PREDICTED: Erinaceus europaeus keratin, type I cytoskeletal 17 (LOC103124738), mRNA
Cluster-33503.126357	--	1449.663	30159.277	-4.3792	6.31E-12	7.48E-09	PREDICTED: Erinaceus europaeus keratin-associated protein 15-1 (LOC103118357), mRNA
Cluster-33503.125840	--	79.504	1528.508	-4.2722	6.86E-12	8.10E-09	PREDICTED: Erinaceus europaeus keratin-associated protein 15-1 (LOC103118357), mRNA
Cluster-33503.149565	--	250.403	4792.036	-4.2609	7.81E-12	9.10E-09	PREDICTED: Erinaceus europaeus keratin-associated protein 15-1 (LOC103118357), mRNA
Cluster-33503.44603	--	6.698	185.444	-4.8732	9.23E-12	1.07E-08	--
Cluster-33503.132637	--	22.566	569.666	-4.6885	9.57E-12	1.09E-08	PREDICTED: Erinaceus europaeus calponin 1 (CNN1), transcript variant X2, mRNA
Cluster-33503.49299	--	577.653	7897.433	-3.7745	9.99E-12	1.13E-08	PREDICTED: Erinaceus europaeus keratin, type II cuticular Hb6 (LOC103109202), mRNA
Cluster-33503.47654	--	722.113	6201.115	-3.103	1.03E-11	1.15E-08	PREDICTED: Erinaceus europaeus keratin-associated protein 2-4-like (LOC103120620), mRNA
Cluster-33503.46178	--	137.058	1212.649	-3.1502	1.05E-11	1.17E-08	PREDICTED: Erinaceus europaeus keratin-associated protein 3-3 (LOC103120617), mRNA
Cluster-33503.132655	--	49.200	1259.730	-4.6918	1.15E-11	1.27E-08	PREDICTED: Erinaceus europaeus calponin 1 (CNN1), transcript variant X2, mRNA
Cluster-33503.125838	--	1461.838	30649.466	-4.3904	1.33E-11	1.45E-08	PREDICTED: Erinaceus europaeus keratin-associated protein 15-1 (LOC103118357), mRNA
Cluster-33503.45898	--	195.411	3436.046	-4.1379	1.45E-11	1.56E-08	PREDICTED: Erinaceus europaeus cornifin (LOC107523341), mRNA
Cluster-33503.123158	--	0.000	97.630	-9.1283	1.53E-11	1.64E-08	Erinaceus europaeus, clone XX-29441557E23, complete sequence
Cluster-33503.9433	--	0.400	987.459	-11.498	1.53E-11	1.64E-08	PREDICTED: Erinaceus europaeus keratin-associated protein 19-2-like (LOC103118359), mRNA

Cluster-33503.42333	--	110.112	3223.954	-4.8765	1.79E-11	1.89E-08	PREDICTED: Erinaceus europaeus keratin-associated protein 8-1 (LOC107523100), mRNA
Cluster-33503.43196	--	6.019	111.663	-4.2664	1.80E-11	1.90E-08	--
Cluster-33503.21664	--	5.478	193.233	-5.1911	1.82E-11	1.91E-08	Atelerix albiventris clone LB4-171F3, complete sequence
Cluster-33503.132652	--	5.078	122.665	-4.6938	1.85E-11	1.94E-08	PREDICTED: Erinaceus europaeus calponin 1 (CNN1), transcript variant X2, mRNA
Cluster-33503.45420	--	99.164	923.854	-3.226	1.89E-11	1.97E-08	--
Cluster-33503.69016	--	198.727	7.072	4.8543	2.00E-11	2.08E-08	Atelerix albiventris clone LB4-464N6, complete sequence
Cluster-33503.42721	--	328.375	12.539	4.7429	2.02E-11	2.09E-08	--
Cluster-33503.125844	--	292.839	5618.395	-4.2639	2.04E-11	2.10E-08	PREDICTED: Erinaceus europaeus keratin-associated protein 15-1 (LOC103118357), mRNA
Cluster-33503.46218	--	2.000	105.958	-5.6689	2.07E-11	2.12E-08	PREDICTED: Erinaceus europaeus keratin, type II cytoskeletal 75-like (LOC103109183), mRNA
Cluster-33503.181714	--	5.219	109.331	-4.4666	2.15E-11	2.20E-08	--
Cluster-33503.129089	--	3571.294	32675.305	-3.1939	2.18E-11	2.22E-08	PREDICTED: Erinaceus europaeus keratin-associated protein 3-3 (LOC103120618), mRNA
Cluster-33503.59505	--	39.727	420.273	-3.4157	2.38E-11	2.39E-08	--
Cluster-33503.123477	--	1682.170	129.410	3.7024	2.48E-11	2.48E-08	PREDICTED: Rousettus aegyptiacus keratin-associated protein 5-2-like (LOC107515091), mRNA
Cluster-33503.179512	--	1.301	102.383	-6.3537	2.50E-11	2.48E-08	PREDICTED: Erinaceus europaeus keratin, type II cytoskeletal 75-like (LOC103109183), mRNA
Cluster-33503.151246	--	10.738	169.358	-4.0173	2.74E-11	2.71E-08	Atelerix albiventris clone LBNL4-89B6, complete sequence
Cluster-33503.27403	--	410.302	8059.343	-4.2975	2.78E-11	2.73E-08	PREDICTED: Erinaceus europaeus keratin-associated protein 15-1 (LOC103118357), mRNA

Cluster-33503.148701	--	220.408	2915.843	-3.7263	2.86E-11	2.80E-08	PREDICTED: Erinaceus europaeus cornifin (LOC107523341), mRNA
Cluster-33503.125842	--	1064.938	18874.829	-4.1482	2.96E-11	2.88E-08	PREDICTED: Erinaceus europaeus keratin-associated protein 15-1 (LOC103118357), mRNA
Cluster-33503.123183	--	111.363	1464.501	-3.7113	3.01E-11	2.91E-08	Pudu mephistophiles isolate MRGPm2 mitochondrion, complete genome
Cluster-33503.126750	--	6.936	155.376	-4.5478	3.25E-11	3.13E-08	PREDICTED: Erinaceus europaeus keratin-associated protein 11-1 (LOC103123006), mRNA
Cluster-33503.45979	--	516.953	10278.342	-4.3147	3.37E-11	3.24E-08	PREDICTED: Erinaceus europaeus keratin-associated protein 15-1 (LOC103118357), mRNA
Cluster-33503.48655	--	74.178	927.040	-3.6528	3.70E-11	3.53E-08	PREDICTED: Erinaceus europaeus keratin, type II microfibrillar, component 7C (LOC103109188), mRNA
Cluster-33503.50076	--	1959.936	26259.208	-3.7443	3.71E-11	3.53E-08	PREDICTED: Erinaceus europaeus keratin, type II cuticular Hb6 (LOC103109202), mRNA
Cluster-33503.122766	--	360.228	6393.779	-4.1515	3.88E-11	3.66E-08	--
Cluster-33503.8257	--	20.167	595.805	-4.9153	4.05E-11	3.80E-08	PREDICTED: Erinaceus europaeus keratin-associated protein 15-1 (LOC103118357), mRNA
Cluster-33503.49625	--	496.002	5756.702	-3.5355	4.13E-11	3.85E-08	PREDICTED: Erinaceus europaeus teratocarcinoma-derived growth factor 1 (TDGF1), mRNA
Cluster-33503.124124	--	68.201	876.177	-3.6909	4.80E-11	4.44E-08	PREDICTED: Erinaceus europaeus keratin-associated protein 3-1 (LOC103120619), mRNA
Cluster-33503.48521	--	469.016	6644.617	-3.826	4.81E-11	4.45E-08	--
Cluster-33503.123283	--	38.118	564.959	-3.9081	5.02E-11	4.62E-08	--
Cluster-33503.126634	--	4.617	109.980	-4.6231	5.31E-11	4.84E-08	PREDICTED: Erinaceus europaeus keratin, type II cytoskeletal 75-like (LOC103109183), mRNA
Cluster-33503.127076	--	52.304	710.790	-3.7761	5.51E-11	4.99E-08	--

Cluster-33503.49887	--	258.898	2806.366	-3.4408	5.82E-11	5.25E-08	PREDICTED: Erinaceus europaeus keratin, type II cuticular Hb1 (LOC103109277), mRNA
Cluster-33503.123055	--	513.339	5154.540	-3.3292	6.79E-11	6.09E-08	PREDICTED: Erinaceus europaeus keratin, type II cuticular Hb6 (LOC103109202), mRNA
Cluster-33503.6480	--	32.286	729.492	-4.5061	8.20E-11	7.21E-08	PREDICTED: Erinaceus europaeus dopachrome tautomerase (DCT), mRNA
Cluster-33503.12480	--	61.986	491.628	-2.9881	8.77E-11	7.66E-08	PREDICTED: Erinaceus europaeus proline rich transmembrane protein 1 (PRRT1), mRNA
Cluster-33503.49065	--	7.634	233.862	-4.9819	9.30E-11	8.07E-08	PREDICTED: Erinaceus europaeus keratin-associated protein 8-1 (LOC107523100), mRNA
Cluster-33503.122429	--	28.743	476.920	-4.0768	9.97E-11	8.62E-08	PREDICTED: Erinaceus europaeus keratin, type II cuticular Hb6 (LOC103109202), mRNA
Cluster-33503.143018	--	11.819	171.774	-3.8878	1.03E-10	8.86E-08	Erinaceus europaeus, clone XX-29460857F24, complete sequence
Cluster-33503.132666	--	6.895	184.698	-4.8325	1.10E-10	9.39E-08	PREDICTED: Erinaceus europaeus calponin 1 (CNN1), transcript variant X2, mRNA
Cluster-33503.45990	--	16.616	230.042	-3.8148	1.12E-10	9.53E-08	PREDICTED: Colobus angolensis palliatus keratin 13, type I (KRT13), transcript variant X2, mRNA
Cluster-33503.47413	--	220.621	2061.192	-3.2254	1.14E-10	9.69E-08	--
Cluster-33503.125109	--	5983.329	68550.458	-3.5183	1.24E-10	1.05E-07	PREDICTED: Erinaceus europaeus keratin-associated protein 7-1 (LOC103122995), mRNA
Cluster-33503.26645	--	40.394	913.611	-4.5148	1.26E-10	1.07E-07	PREDICTED: Erinaceus europaeus keratin-associated protein 15-1 (LOC103118357), mRNA
Cluster-33503.123780	--	216.445	2665.415	-3.6249	1.42E-10	1.19E-07	PREDICTED: Erinaceus europaeus keratin-associated protein 3-1 (LOC103120619), mRNA
Cluster-33503.28424	--	3205.704	288.396	3.4741	1.42E-10	1.19E-07	PREDICTED: Erinaceus europaeus keratin-associated protein 10-8-like (LOC103128195), mRNA
Cluster-33503.49008	--	110.085	2.623	5.3646	1.42E-10	1.19E-07	Mus musculus cDNA clone IMAGE:40049614

Cluster-33503.180055	--	2.760	78.741	-4.839	1.43E-10	1.19E-07	--
Cluster-33503.7610	--	3.000	130.118	-5.5606	1.70E-10	1.39E-07	Atelerix albiventris clone LB4-482N5, complete sequence
Cluster-33503.127106	--	67.778	477.983	-2.815	1.85E-10	1.50E-07	Homo sapiens chromosome 17 clone WI2-3468M6, complete sequence
Cluster-33503.50047	--	736.409	8884.463	-3.5933	1.87E-10	1.51E-07	PREDICTED: Erinaceus europaeus keratin-associated protein 9-3-like (LOC103120634), mRNA
Cluster-33503.7839	--	13.096	146.619	-3.4844	1.87E-10	1.51E-07	PREDICTED: Macaca mulatta diacylglycerol kinase gamma (DGKG), transcript variant X2, mRNA
Cluster-33503.132620	--	5.179	118.202	-4.6128	1.92E-10	1.54E-07	PREDICTED: Erinaceus europaeus calponin 1 (CNN1), transcript variant X2, mRNA
Cluster-33503.140105	--	157.714	1408.960	-3.1578	1.96E-10	1.57E-07	PREDICTED: Erinaceus europaeus family with sequence similarity 46 member B (FAM46B), mRNA
Cluster-33503.146846	--	264.334	24.266	3.4467	1.97E-10	1.57E-07	PREDICTED: Ursus maritimus keratin-associated protein 3-1-like (LOC103662054), mRNA
Cluster-33503.28410	--	908.580	85.892	3.4023	2.03E-10	1.60E-07	PREDICTED: Erinaceus europaeus keratin-associated protein 10-12-like (LOC107523376), partial mRNA
Cluster-33503.66173	--	39.148	328.207	-3.0789	2.03E-10	1.60E-07	PREDICTED: Erinaceus europaeus smoothelin (SMTN), transcript variant X2, mRNA
Cluster-33503.38546	--	18.692	211.684	-3.5146	2.28E-10	1.78E-07	PREDICTED: Erinaceus europaeus keratin, type II cytoskeletal 75-like (LOC103109183), mRNA
Cluster-33503.50077	--	1154.721	7674.744	-2.733	2.34E-10	1.82E-07	Atelerix albiventris clone LBNL4-89B6, complete sequence
Cluster-33503.125464	--	60.780	795.813	-3.7207	2.44E-10	1.89E-07	PREDICTED: Erinaceus europaeus keratin-associated protein 3-1 (LOC103120619), mRNA
Cluster-33503.124400	--	297.600	4289.285	-3.8518	2.50E-10	1.93E-07	PREDICTED: Erinaceus europaeus keratin, type II cuticular Hb6 (LOC103109202), mRNA

Cluster-33503.132645	--	20.489	366.013	-4.1933	2.60E-10	1.99E-07	PREDICTED: Erinaceus europaeus calponin 1 (CNN1), transcript variant X2, mRNA
Cluster-33503.125463	--	1322.174	14262.759	-3.4317	2.64E-10	2.02E-07	PREDICTED: Erinaceus europaeus keratin-associated protein 3-1 (LOC103120619), mRNA
Cluster-33503.47648	--	24.153	252.725	-3.3987	2.69E-10	2.04E-07	--
Cluster-33503.125845	--	13.610	343.011	-4.6993	3.02E-10	2.28E-07	PREDICTED: Erinaceus europaeus keratin-associated protein 15-1 (LOC103118357), mRNA
Cluster-33503.164323	--	138.032	1478.383	-3.4253	3.09E-10	2.32E-07	PREDICTED: Erinaceus europaeus keratin-associated protein 3-1 (LOC103120619), mRNA
Cluster-33503.124956	--	32.124	493.439	-3.9514	3.13E-10	2.34E-07	--
Cluster-33503.37532	--	51.840	995.139	-4.2542	3.17E-10	2.36E-07	PREDICTED: Erinaceus europaeus sperm mitochondria associated cysteine rich protein (SMCP), mRNA
Cluster-33503.49939	--	2.319	139.307	-6.0619	3.17E-10	2.36E-07	PREDICTED: Erinaceus europaeus keratin-associated protein 8-1 (LOC107523100), mRNA
Cluster-33503.49055	--	222.422	18.478	3.5798	3.31E-10	2.45E-07	PREDICTED: Cavia porcellus keratin-associated protein 12-3-like (LOC100725711), mRNA
Cluster-33503.132640	--	4.436	139.253	-5.0729	3.65E-10	2.67E-07	PREDICTED: Erinaceus europaeus calponin 1 (CNN1), transcript variant X2, mRNA
Cluster-33503.45669	--	39.276	502.382	-3.6872	4.07E-10	2.96E-07	--
Cluster-33503.159540	--	5.098	133.861	-4.7113	4.16E-10	3.01E-07	Atelerix albiventris clone LB4-234A16, complete sequence
Cluster-33503.124699	--	20.234	275.266	-3.76	4.18E-10	3.01E-07	--
Cluster-33503.47777	--	219.957	7.364	4.9516	4.24E-10	3.05E-07	Atelerix albiventris clone LB4-244I20, complete sequence
Cluster-33503.44495	--	55.614	626.700	-3.5001	4.67E-10	3.32E-07	--
Cluster-33503.47664	--	7018.770	60782.758	-3.1145	4.72E-10	3.34E-07	PREDICTED: Erinaceus europaeus keratin-associated protein 2-4-like (LOC103120620), mRNA

Cluster-33503.80919	--	244.631	10.701	4.5519	4.78E-10	3.37E-07	Atelerix albiventris clone LB4-464N6, complete sequence
Cluster-27661.0	--	71.801	0.000	8.5676	5.33E-10	3.74E-07	PREDICTED: Capsicum annuum uncharacterized LOC107867012 (LOC107867012), ncRNA
Cluster-33503.45089	--	506.629	42.457	3.5781	5.65E-10	3.91E-07	--
Cluster-33503.56756	--	282.733	2678.286	-3.2435	6.13E-10	4.22E-07	PREDICTED: Erinaceus europaeus glutamyl aminopeptidase (ENPEP), mRNA
Cluster-33503.25476	--	15.269	2038.948	-7.0722	6.64E-10	4.53E-07	PREDICTED: Erinaceus europaeus serine protease inhibitor Kazal-type 6-like (LOC103107621), mRNA
Cluster-33503.50041	--	925.940	8263.384	-3.1585	6.62E-10	4.53E-07	PREDICTED: Erinaceus europaeus keratin-associated protein 9-3-like (LOC103120621), mRNA
Cluster-33503.8403	--	0.000	76.414	-8.7695	6.92E-10	4.70E-07	--
Cluster-33503.7906	--	59.689	698.455	-3.5599	7.27E-10	4.91E-07	PREDICTED: Erinaceus europaeus keratin-associated protein 7-1 (LOC103122995), mRNA
Cluster-30607.1	--	66.208	0.000	8.4509	7.39E-10	4.97E-07	PREDICTED: Capsicum annuum glycine-rich protein DC7.1-like (LOC107864362), transcript variant X1, mRNA
Cluster-33503.136581	--	5974.303	34645.920	-2.5358	7.56E-10	5.04E-07	PREDICTED: Erinaceus europaeus desmin (DES), mRNA
Cluster-33503.50146	--	40.495	572.531	-3.8365	7.55E-10	5.04E-07	PREDICTED: Erinaceus europaeus keratin-associated protein 7-1 (LOC103122995), mRNA
Cluster-33503.47411	--	1020.564	12146.705	-3.5738	7.62E-10	5.07E-07	PREDICTED: Erinaceus europaeus keratin-associated protein 7-1 (LOC103122995), mRNA
Cluster-33503.66183	--	728.823	4483.860	-2.6218	8.95E-10	5.93E-07	PREDICTED: Erinaceus europaeus smoothelin (SMTN), transcript variant X2, mRNA
Cluster-33503.28421	--	222.635	12.397	4.1626	9.46E-10	6.25E-07	--
Cluster-33503.16321	--	33.994	429.677	-3.6752	9.64E-10	6.34E-07	PREDICTED: Erinaceus europaeus keratin-associated protein 3-1 (LOC103120619), mRNA

Cluster-33503.66175	--	11.202	180.263	-4.011	9.89E-10	6.47E-07	PREDICTED: Erinaceus europaeus smoothelin (SMTN), transcript variant X2, mRNA
Cluster-33503.130112	--	24.699	3563.857	-7.1797	1.02E-09	6.65E-07	PREDICTED: Erinaceus europaeus keratin-associated protein 13-2-like (LOC103118353), mRNA
Cluster-33503.130148	--	2.139	106.703	-5.7497	1.03E-09	6.72E-07	PREDICTED: Erinaceus europaeus keratin-associated protein 8-1 (LOC107523100), mRNA
Cluster-33503.50139	--	501.657	6762.373	-3.7542	1.06E-09	6.85E-07	PREDICTED: Erinaceus europaeus keratin-associated protein 7-1 (LOC103122995), mRNA
Cluster-33503.93249	--	167.507	4.703	5.1821	1.07E-09	6.93E-07	Atelerix albiventris clone LB4-108K18, complete sequence
Cluster-33503.124736	--	4628.256	48148.559	-3.3791	1.07E-09	6.93E-07	PREDICTED: Erinaceus europaeus keratin-associated protein 7-1 (LOC103122995), mRNA
Cluster-33503.52738	--	203.813	2201.356	-3.4351	1.28E-09	8.25E-07	PREDICTED: Erinaceus europaeus keratin-associated protein 13-2-like (LOC103118344), mRNA
Cluster-33503.50142	--	1419.636	16887.101	-3.5728	1.34E-09	8.56E-07	PREDICTED: Erinaceus europaeus keratin-associated protein 7-1 (LOC103122995), mRNA
Cluster-33503.50147	--	3330.100	43729.384	-3.7152	1.33E-09	8.56E-07	PREDICTED: Erinaceus europaeus keratin-associated protein 7-1 (LOC103122995), mRNA
Cluster-33503.48697	--	73.155	1059.071	-3.8648	1.36E-09	8.71E-07	PREDICTED: Erinaceus europaeus keratin-associated protein 7-1 (LOC103122995), mRNA
Cluster-33503.43838	--	62.061	441.511	-2.8325	1.39E-09	8.85E-07	PREDICTED: Erinaceus europaeus phosphoglucomutase 5 (PGM5), mRNA
Cluster-33503.44593	--	20.364	2703.133	-7.0614	1.41E-09	8.99E-07	PREDICTED: Erinaceus europaeus keratin-associated protein 19-2-like (LOC103118359), mRNA
Cluster-33503.46860	--	509.419	4378.609	-3.1028	1.47E-09	9.33E-07	PREDICTED: Erinaceus europaeus musculoskeletal, embryonic nuclear protein 1 (MUSTN1), mRNA
Cluster-33503.46861	--	71.757	634.374	-3.1439	1.56E-09	9.80E-07	PREDICTED: Erinaceus europaeus musculoskeletal, embryonic nuclear protein 1 (MUSTN1), mRNA

Cluster-33503.129221	--	1.960	75.144	-5.3134	1.81E-09	1.13E-06	PREDICTED: Erinaceus europaeus keratin-associated protein 15-1 (LOC103118357), mRNA
Cluster-33503.47641	--	583.545	4612.522	-2.9836	1.83E-09	1.14E-06	PREDICTED: Erinaceus europaeus keratin-associated protein 2-4-like (LOC103120620), mRNA
Cluster-62648.0	--	0.721	93.662	-7.2043	1.88E-09	1.16E-06	PREDICTED: Gossypium hirsutum non-specific lipid-transfer protein-like (LOC107914538), mRNA
Cluster-33503.128479	--	87.376	967.097	-3.4754	1.91E-09	1.18E-06	PREDICTED: Erinaceus europaeus keratin-associated protein 3-1 (LOC103120619), mRNA
Cluster-33503.47662	--	86.883	702.493	-3.0197	1.98E-09	1.22E-06	--
Cluster-33503.186224	--	0.400	84.253	-7.9506	2.00E-09	1.23E-06	Atelerix albiventris clone LB4-344F17, complete sequence
Cluster-33503.125099	--	24.189	355.668	-3.8828	2.19E-09	1.34E-06	--
Cluster-33503.180067	--	0.000	55.074	-8.2974	2.21E-09	1.35E-06	Gossypium hirsutum proline-rich protein-1 (prp1) gene, complete cds
Cluster-33503.125820	--	640.766	4176.916	-2.7036	2.48E-09	1.50E-06	Erinaceus europaeus, clone XX-29460857F24, complete sequence
Cluster-33503.72569	--	0.000	51.223	-8.1936	2.74E-09	1.64E-06	--
Cluster-33503.122588	--	83.005	639.358	-2.9518	2.77E-09	1.66E-06	PREDICTED: Erinaceus europaeus keratin, type II cuticular Hb6 (LOC103109202), mRNA
Cluster-4249.0	--	0.000	52.556	-8.2301	2.80E-09	1.67E-06	PREDICTED: Gossypium hirsutum protodermal factor 1-like (LOC107959669), mRNA
Cluster-33503.46682	--	2883.758	224.894	3.6817	2.87E-09	1.71E-06	PREDICTED: Erinaceus europaeus keratin-associated protein 9-2 (LOC103124750), mRNA
Cluster-33503.50140	--	795.506	9433.935	-3.5688	2.99E-09	1.77E-06	PREDICTED: Erinaceus europaeus keratin-associated protein 7-1 (LOC103122995), mRNA
Cluster-33503.264	--	0.440	98.539	-7.4061	3.01E-09	1.78E-06	--
Cluster-33503.127985	--	2232.848	26399.470	-3.5639	3.04E-09	1.79E-06	PREDICTED: Erinaceus europaeus keratin-associated protein 7-1 (LOC103122995), mRNA

Cluster-33503.184166	--	3.200	73.841	-4.4902	3.03E-09	1.79E-06	Mus musculus potassium channel, subfamily K, member 3 (Kcnk3), mRNA
Cluster-33503.28384	--	48.339	919.673	-4.238	3.10E-09	1.82E-06	--
Cluster-33503.145858	--	4.936	172.446	-5.2122	3.19E-09	1.87E-06	--
Cluster-33503.44448	--	340.723	23.576	3.8639	3.38E-09	1.97E-06	PREDICTED: Erinaceus europaeus keratin-associated protein 9-2 (LOC103124750), mRNA
Cluster-33503.122759	--	13.033	200.738	-3.9824	3.67E-09	2.13E-06	PREDICTED: Odobenus rosmarus divergens keratin, type I cuticular Ha3-II (LOC101386909), transcript variant X2, mRNA
Cluster-33503.123292	--	7.116	154.528	-4.4959	3.83E-09	2.21E-06	--
Cluster-33503.179567	--	0.000	47.791	-8.0942	3.94E-09	2.27E-06	--
Cluster-33503.49053	--	705.726	46.693	3.9187	4.06E-09	2.33E-06	PREDICTED: Erinaceus europaeus keratin-associated protein 12-1-like (LOC103111332), mRNA
Cluster-33503.48387	--	24.807	372.298	-3.9344	4.10E-09	2.34E-06	PREDICTED: Erinaceus europaeus keratin, type II cuticular Hb6 (LOC103109202), mRNA
Cluster-33503.8463	--	3.497	89.053	-4.6997	4.30E-09	2.44E-06	PREDICTED: Erinaceus europaeus keratin-associated protein 13-2 (LOC103118343), mRNA
Cluster-33503.72566	--	0.360	77.845	-7.834	4.31E-09	2.44E-06	--
Cluster-33503.74360	--	227.889	10.751	4.419	4.41E-09	2.49E-06	PREDICTED: Erinaceus europaeus keratin-associated protein 9-2 (LOC103124750), mRNA
Cluster-33503.28405	--	459.485	34.449	3.7424	4.62E-09	2.60E-06	PREDICTED: Erinaceus europaeus keratin-associated protein 10-8-like (LOC103126023), mRNA
Cluster-33503.122418	--	416.102	4016.035	-3.2725	4.64E-09	2.61E-06	PREDICTED: Erinaceus europaeus keratin-associated protein 7-1 (LOC103122995), mRNA
Cluster-33503.49753	--	162.127	13.910	3.5328	4.68E-09	2.62E-06	PREDICTED: Erinaceus europaeus keratin-associated protein 12-1-like (LOC103111332), mRNA
Cluster-33503.123665	--	699.665	6550.301	-3.2276	5.00E-09	2.79E-06	--

Cluster-33503.47412	--	3.259	78.775	-4.7059	5.42E-09	3.02E-06	PREDICTED: Erinaceus europaeus keratin-associated protein 7-1 (LOC103122995), mRNA
Cluster-33503.127688	--	277.109	21.697	3.6906	5.60E-09	3.10E-06	PREDICTED: Cavia porcellus ice-structuring glycoprotein-like (LOC101787386), mRNA
Cluster-33503.122689	--	2822.366	24367.371	-3.1102	6.04E-09	3.31E-06	--
Cluster-33503.57731	--	1572.132	11650.402	-2.8898	6.03E-09	3.31E-06	PREDICTED: Erinaceus europaeus keratin, type I cytoskeletal 14 (LOC103124740), mRNA
Cluster-33503.127726	--	19.355	232.191	-3.6123	7.28E-09	3.96E-06	PREDICTED: Erinaceus europaeus keratin-associated protein 3-1 (LOC103120619), mRNA
Cluster-33503.38331	--	7.442	173.237	-4.5143	7.93E-09	4.29E-06	Atelerix albiventris clone LB4-464N6, complete sequence
Cluster-33503.44724	--	0.721	85.491	-7.0657	8.24E-09	4.44E-06	PREDICTED: Erinaceus europaeus keratin, type II cytoskeletal 75-like (LOC103109183), mRNA
Cluster-33503.113979	--	3.679	94.066	-4.6294	8.31E-09	4.47E-06	Felis catus BAC clone FCAB-91H12 from chromosome unknown, complete sequence
Cluster-33503.145283	--	2626.526	372.292	2.8171	8.50E-09	4.56E-06	PREDICTED: Erinaceus europaeus keratin-associated protein 16-1 (LOC103124760), mRNA
Cluster-33503.186705	--	0.000	44.630	-7.9944	9.37E-09	4.98E-06	Ovis canadensis canadensis isolate 43U chromosome 1 sequence
Cluster-33503.48895	--	253.797	2614.656	-3.3676	9.42E-09	4.99E-06	PREDICTED: Erinaceus europaeus keratin, type II cuticular Hb6 (LOC103109202), mRNA
Cluster-76259.0	--	48.182	0.000	7.991	9.52E-09	5.04E-06	PREDICTED: Nicotiana sylvestris ribulose bisphosphate carboxylase/oxygenase activase 2, chloroplastic (LOC104213181), mRNA
Cluster-33503.47504	--	323.565	2972.383	-3.2017	9.77E-09	5.15E-06	PREDICTED: Erinaceus europaeus keratin-associated protein 7-1 (LOC103122995), mRNA
Cluster-33503.126754	--	6.178	78.031	-3.6667	1.04E-08	5.43E-06	PREDICTED: Erinaceus europaeus keratin-associated protein 11-1 (LOC103123006), mRNA

Cluster-33503.27749	--	28.206	276.887	-3.3171	1.05E-08	5.52E-06	--
Cluster-33503.57704	--	2.280	5115.090	-11.169	1.06E-08	5.54E-06	PREDICTED: Erinaceus europaeus keratin, type I cytoskeletal 17 (LOC103124738), mRNA
Cluster-33503.123781	--	344.355	2920.123	-3.0864	1.06E-08	5.55E-06	PREDICTED: Erinaceus europaeus keratin-associated protein 3-3 (LOC103120617), mRNA
Cluster-33503.28401	--	537.940	47.917	3.4874	1.23E-08	6.33E-06	PREDICTED: Erinaceus europaeus keratin-associated protein 10-12-like (LOC107523376), partial mRNA
Cluster-33503.126615	--	101.293	786.828	-2.9652	1.27E-08	6.51E-06	PREDICTED: Erinaceus europaeus keratin-associated protein 3-3 (LOC103120617), mRNA
Cluster-29813.0	--	44.070	0.000	7.8711	1.30E-08	6.66E-06	PREDICTED: Capsicum annuum protein TAP1-like (LOC107845879), mRNA
Cluster-33503.28417	--	631.490	57.640	3.4533	1.32E-08	6.76E-06	--
Cluster-33503.184371	--	0.620	72.722	-6.8925	1.37E-08	6.99E-06	PREDICTED: Erinaceus europaeus Rh family C glycoprotein (RHCG), transcript variant X2, mRNA
Cluster-33503.177917	--	1.199	62.030	-5.9712	1.42E-08	7.24E-06	PREDICTED: Erinaceus europaeus calponin 1 (CNN1), transcript variant X1, mRNA
Cluster-33503.127729	--	303.046	28.969	3.3885	1.43E-08	7.28E-06	PREDICTED: Canis lupus familiaris keratin-associated protein 5-2-like (LOC102153243), transcript variant X2, mRNA
Cluster-33503.28368	--	121.543	10.448	3.5485	1.56E-08	7.88E-06	PREDICTED: Erinaceus europaeus keratin-associated protein 10-8-like (LOC103128195), mRNA
Cluster-11982.0	--	3.098	104.576	-5.0887	1.57E-08	7.89E-06	--
Cluster-33503.124573	--	6.217	145.905	-4.5781	1.68E-08	8.39E-06	PREDICTED: Erinaceus europaeus keratin-associated protein 8-1 (LOC107523100), mRNA
Cluster-33503.42038	--	33.193	598.073	-4.1857	1.68E-08	8.39E-06	--
Cluster-33503.66182	--	72.603	416.101	-2.5229	1.75E-08	8.65E-06	PREDICTED: Erinaceus europaeus smoothelin (SMTN), transcript variant X2, mRNA
Cluster-33503.47655	--	227.264	1492.201	-2.7171	1.92E-08	9.45E-06	--

Cluster-33503.130496	--	97.267	3.830	4.7364	1.99E-08	9.76E-06	Atelerix albiventris clone LB4-498C22, complete sequence
Cluster-33503.89151	--	0.000	46.419	-8.0615	2.12E-08	1.03E-05	PREDICTED: Erinaceus europaeus tyrosinase (TYR), mRNA
Cluster-33503.137144	--	28.794	232.257	-3.0099	2.22E-08	1.08E-05	Atelerix albiventris clone LB4-226M9, complete sequence
Cluster-33503.65053	--	82.750	550.191	-2.7243	2.24E-08	1.08E-05	PREDICTED: Erinaceus europaeus synaptopodin 2 (SYNPO2), mRNA
Cluster-33503.128632	--	40.436	403.011	-3.3314	2.38E-08	1.15E-05	--
Cluster-33503.44586	--	0.000	46.377	-8.0481	2.46E-08	1.19E-05	Rhesus Macaque BAC CH250-204A18 () complete sequence
Cluster-33503.132564	--	31.416	215.799	-2.7944	2.47E-08	1.19E-05	PREDICTED: Equus asinus homeobox D9 (HOXD9), mRNA
Cluster-33503.178208	--	0.400	270.403	-9.6286	2.67E-08	1.28E-05	PREDICTED: Pongo abelii keratin-associated protein 19-1 (LOC103888873), mRNA
Cluster-33503.1787	--	1.819	63.738	-5.1223	2.79E-08	1.33E-05	Homo sapiens 12 BAC RP11-25I15 (Roswell Park Cancer Institute Human BAC Library) complete sequence
Cluster-33503.44584	--	30.873	4945.720	-7.329	2.97E-08	1.41E-05	PREDICTED: Erinaceus europaeus keratin-associated protein 19-3-like (LOC103118358), mRNA
Cluster-33503.44588	--	1.199	70.880	-6.1379	3.04E-08	1.44E-05	PREDICTED: Erinaceus europaeus keratin-associated protein 19-2-like (LOC103118359), mRNA
Cluster-33503.46509	--	20.700	277.330	-3.7038	3.34E-08	1.58E-05	PREDICTED: Erinaceus europaeus myosin, heavy chain 11, smooth muscle (MYH11), transcript variant X2, mRNA
Cluster-33503.132624	--	0.799	63.668	-6.6229	3.60E-08	1.70E-05	PREDICTED: Erinaceus europaeus calponin 1 (CNN1), transcript variant X2, mRNA
Cluster-33503.123944	--	14.777	130.531	-3.1582	3.68E-08	1.73E-05	--

Cluster-33503.97741	--	61.692	344.101	-2.4834	3.73E-08	1.75E-05	Atelerix albiventris clone LB4-307O5, complete sequence
Cluster-33503.57735	--	1029.093	7333.824	-2.8336	3.77E-08	1.76E-05	PREDICTED: Marmota marmota marmota keratin, type I cytoskeletal 14 (LOC107150594), mRNA
Cluster-33503.76358	--	23.647	319.933	-3.765	4.02E-08	1.87E-05	Atelerix albiventris clone LB4-244I20, complete sequence
Cluster-33503.43391	--	11.215	133.246	-3.5682	4.13E-08	1.92E-05	--
Cluster-33503.23004	--	482.773	3602.146	-2.8992	4.24E-08	1.97E-05	PREDICTED: Erinaceus europaeus repetin (RPTN), mRNA
Cluster-33503.128164	--	48.305	404.619	-3.0758	4.34E-08	2.01E-05	PREDICTED: Rattus norvegicus keratin associated protein 3-2 (Krtap3-2), mRNA
Cluster-33503.123479	--	321.165	34.179	3.2336	4.53E-08	2.08E-05	Atelerix albiventris clone LB4-372K23, complete sequence
Cluster-33503.125423	--	535.642	50.857	3.3935	4.54E-08	2.09E-05	--
Cluster-33503.45090	--	349.408	46.099	2.9253	4.92E-08	2.24E-05	PREDICTED: Rousettus aegyptiacus keratin-associated protein 5-2-like (LOC107515091), mRNA
Cluster-33503.8305	--	2.319	85.734	-5.3553	4.95E-08	2.25E-05	PREDICTED: Erinaceus europaeus keratin-associated protein 8-1 (LOC107523100), mRNA
Cluster-33503.132623	--	3.621	65.463	-4.2171	4.99E-08	2.26E-05	PREDICTED: Erinaceus europaeus calponin 1 (CNN1), transcript variant X1, mRNA
Cluster-33503.37528	--	21.175	279.600	-3.7052	5.52E-08	2.49E-05	PREDICTED: Erinaceus europaeus sperm mitochondria associated cysteine rich protein (SMCP), mRNA
Cluster-33503.127689	--	213.225	22.220	3.2522	5.59E-08	2.52E-05	PREDICTED: Cavia porcellus solute carrier family 19 (thiamine transporter), member 3 (Slc19a3), transcript variant X5, mRNA
Cluster-33503.129200	--	1226.357	5710.244	-2.2192	6.13E-08	2.75E-05	PREDICTED: Erinaceus europaeus ovo like zinc finger 1 (OVOL1), mRNA

Cluster-33503.44567	--	0.620	69.124	-6.8172	6.14E-08	2.75E-05	PREDICTED: Erinaceus europaeus keratin-associated protein 19-3-like (LOC103118358), mRNA
Cluster-33503.9158	--	7.437	158.201	-4.4543	6.32E-08	2.82E-05	--
Cluster-33503.68984	--	2.758	399.776	-7.2581	6.49E-08	2.88E-05	PREDICTED: Chlorocebus sabaeus keratin 6A (KRT6A), mRNA
Cluster-33503.36295	--	255.332	1664.372	-2.7044	6.61E-08	2.93E-05	PREDICTED: Erinaceus europaeus striated muscle preferentially expressed protein kinase (LOC103121502), transcript variant X1, mRNA
Cluster-33503.66184	--	179.445	924.536	-2.3623	6.62E-08	2.93E-05	PREDICTED: Erinaceus europaeus smoothelin (SMTN), transcript variant X2, mRNA
Cluster-33503.20855	--	63.903	466.592	-2.8691	6.73E-08	2.97E-05	PREDICTED: Erinaceus europaeus family with sequence similarity 46 member B (FAM46B), mRNA
Cluster-33503.123664	--	2051.640	14334.630	-2.805	6.75E-08	2.97E-05	PREDICTED: Erinaceus europaeus keratin-associated protein 9-3-like (LOC103120634), mRNA
Cluster-33503.113973	--	6.439	104.409	-3.9987	6.96E-08	3.06E-05	Erinaceus europaeus, clone XX-29994857A5, complete sequence
Cluster-33503.69002	--	3.157	475.904	-7.3042	7.13E-08	3.12E-05	PREDICTED: Erinaceus europaeus keratin, type II cytoskeletal 6A-like (LOC103109273), mRNA
Cluster-84051.8	--	36.653	0.000	7.5972	7.23E-08	3.16E-05	Capsicum annuum 1-aminocyclopropane-1-carboxylate oxidase-like (LOC107859634), mRNA
Cluster-33503.46065	--	30.616	286.412	-3.2423	7.53E-08	3.28E-05	PREDICTED: Erinaceus europaeus keratin-associated protein 9-3-like (LOC103120634), mRNA
Cluster-33503.44575	--	0.799	77.259	-6.8721	7.55E-08	3.29E-05	PREDICTED: Erinaceus europaeus keratin-associated protein 19-2-like (LOC103118359), mRNA
Cluster-33503.48728	--	1437.074	12057.145	-3.0691	8.13E-08	3.51E-05	PREDICTED: Erinaceus europaeus keratin-associated protein 13-2-like (LOC103118344), mRNA
Cluster-33503.41792	--	0.400	252.542	-9.5296	8.29E-08	3.58E-05	PREDICTED: Erinaceus europaeus keratin-associated protein 19-2-like (LOC103118359), mRNA

Cluster-33503.142389	--	1.340	48.899	-5.2736	8.50E-08	3.66E-05	PREDICTED: Myotis brandtii diacylglycerol kinase, gamma 90kDa (DGKG), mRNA
Cluster-33503.45419	--	14.397	117.817	-3.061	8.51E-08	3.66E-05	PREDICTED: Erinaceus europaeus keratin-associated protein 3-3 (LOC103120617), mRNA
Cluster-33503.44590	--	0.400	62.029	-7.5043	8.81E-08	3.78E-05	--
Cluster-33503.7039	--	21.647	232.884	-3.4508	9.02E-08	3.86E-05	Homo sapiens myocardin (MYOCD), transcript variant 2, mRNA
Cluster-33503.179377	--	10.092	1254.186	-6.962	9.06E-08	3.87E-05	--
Cluster-33503.8202	--	1.999	55.281	-4.8584	9.27E-08	3.95E-05	--
Cluster-28368.0	--	35.369	0.000	7.5525	9.39E-08	3.99E-05	Capsicum annuum, 6 clones, complete sequence
Cluster-33503.28426	--	308.441	27.085	3.5124	1.01E-07	4.27E-05	PREDICTED: Erinaceus europaeus keratin-associated protein 10-8-like (LOC103128195), mRNA
Cluster-33503.16012	--	152.250	13.767	3.4574	1.01E-07	4.28E-05	PREDICTED: Sus scrofa mab-21-like 1 (C. elegans) (MAB21L1), mRNA
Cluster-33503.68987	--	2.280	67.954	-5.0431	1.02E-07	4.31E-05	PREDICTED: Erinaceus europaeus keratin, type II cytoskeletal 6A-like (LOC103109273), mRNA
Cluster-33503.46487	--	49.132	471.247	-3.2737	1.08E-07	4.51E-05	PREDICTED: Erinaceus europaeus keratin-associated protein 9-3-like (LOC103120634), mRNA
Cluster-33503.177471	--	0.000	39.368	-7.8121	1.10E-07	4.58E-05	PREDICTED: Erinaceus europaeus keratin-associated protein 8-1 (LOC107523100), mRNA
Cluster-33503.149083	--	148.376	9.725	3.9073	1.17E-07	4.89E-05	Chlorocebus aethiops BAC clone CH252-264L10 from chromosome 11, complete sequence
Cluster-33503.123932	--	2087.243	11512.182	-2.4638	1.23E-07	5.11E-05	PREDICTED: Erinaceus europaeus keratin, type II cuticular Hb5 (LOC103109186), mRNA
Cluster-33503.132657	--	3.079	68.521	-4.6144	1.23E-07	5.11E-05	PREDICTED: Erinaceus europaeus calponin 1 (CNN1), transcript variant X2, mRNA
Cluster-33503.50144	--	4.578	72.245	-4.0159	1.25E-07	5.18E-05	PREDICTED: Erinaceus europaeus keratin-associated protein 7-1 (LOC103122995), mRNA

Cluster-33503.188550	--	0.000	33.317	-7.5723	1.27E-07	5.26E-05	Atelerix albiventris clone LB4-171F3, complete sequence
Cluster-33503.98545	--	134.238	655.597	-2.2899	1.28E-07	5.27E-05	--
Cluster-33503.123829	--	290.915	1281.097	-2.14	1.30E-07	5.36E-05	PREDICTED: Erinaceus europaeus keratin-associated protein 4-11-like (LOC103120636), transcript variant X1, mRNA
Cluster-33503.122589	--	15.952	207.812	-3.7406	1.31E-07	5.38E-05	PREDICTED: Erinaceus europaeus keratin, type II cuticular Hb6 (LOC103109202), mRNA
Cluster-33503.47661	--	29.932	229.068	-2.9512	1.34E-07	5.48E-05	PREDICTED: Erinaceus europaeus keratin-associated protein 2-4-like (LOC103120620), mRNA
Cluster-33503.114380	--	137.570	14.184	3.2891	1.41E-07	5.77E-05	Atelerix albiventris clone LB4-244I20, complete sequence
Cluster-33503.164222	--	5.059	75.912	-3.8597	1.49E-07	6.07E-05	Atelerix albiventris clone LB4-372K23, complete sequence
Cluster-33503.127243	--	35.840	402.543	-3.5084	1.50E-07	6.10E-05	PREDICTED: Erinaceus europaeus keratin-associated protein 7-1 (LOC103122995), mRNA
Cluster-33503.126372	--	68.601	413.717	-2.6022	1.51E-07	6.15E-05	PREDICTED: Erinaceus europaeus keratin-associated protein 3-3 (LOC103120618), mRNA
Cluster-33503.122788	--	1613.900	9858.147	-2.6106	1.52E-07	6.16E-05	PREDICTED: Chinchilla lanigera uncharacterized LOC102011827 (LOC102011827), ncRNA
Cluster-33503.44823	--	62.257	4.827	3.7047	1.52E-07	6.16E-05	Atelerix albiventris clone LB4-495B20, complete sequence
Cluster-33503.9846	--	12.135	116.726	-3.2896	1.56E-07	6.30E-05	Homo sapiens potassium voltage-gated channel, shaker-related subfamily, beta member 1, mRNA (cDNA clone MGC:44147 IMAGE:5286833), complete cds
Cluster-33503.129201	--	6.859	73.777	-3.4578	1.65E-07	6.65E-05	PREDICTED: Erinaceus europaeus ovo like zinc finger 1 (OVOL1), mRNA

Cluster-33503.47989	--	438.892	3281.856	-2.904	1.73E-07	6.96E-05	PREDICTED: Erinaceus europaeus keratin-associated protein 9-3-like (LOC103120634), mRNA
Cluster-33503.132649	--	0.620	50.884	-6.3788	1.76E-07	7.08E-05	PREDICTED: Erinaceus europaeus calponin 1 (CNN1), transcript variant X2, mRNA
Cluster-33503.190038	--	6.796	79.585	-3.6023	1.78E-07	7.12E-05	Erinaceus europaeus, clone XX-29994857A5, complete sequence
Cluster-33503.46863	--	33.066	282.066	-3.0953	1.86E-07	7.42E-05	PREDICTED: Erinaceus europaeus musculoskeletal, embryonic nuclear protein 1 (MUSTN1), mRNA
Cluster-33503.47653	--	11.919	143.900	-3.6196	1.87E-07	7.44E-05	--
Cluster-33503.42377	--	8.238	80.528	-3.321	1.89E-07	7.52E-05	--
Cluster-33503.148394	--	0.000	30.816	-7.4602	2.14E-07	8.46E-05	Atelerix albiventris clone LBNL4-89B6, complete sequence
Cluster-33503.127725	--	19.750	210.474	-3.4364	2.21E-07	8.67E-05	PREDICTED: Erinaceus europaeus keratin-associated protein 3-1 (LOC103120619), mRNA
Cluster-33503.179639	--	0.000	36.387	-7.697	2.35E-07	9.17E-05	PREDICTED: Erinaceus europaeus keratin-associated protein 19-3-like (LOC103118358), mRNA
Cluster-33503.146425	--	115.462	6.252	4.1923	2.36E-07	9.20E-05	--
Cluster-33503.103227	--	13.148	1506.190	-6.8565	2.41E-07	9.37E-05	PREDICTED: Erinaceus europaeus keratin, type II cytoskeletal 6A-like (LOC103109273), mRNA
Cluster-33503.111314	--	15.291	143.219	-3.2622	2.47E-07	9.58E-05	PREDICTED: Erinaceus europaeus trafficking protein particle complex 3 like (TRAPPC3L), mRNA
Cluster-33503.180445	--	11.759	97.297	-3.0637	2.47E-07	9.58E-05	PREDICTED: Erinaceus europaeus ankyrin repeat domain 63 (ANKRD63), mRNA
Cluster-33503.178323	--	8.936	92.121	-3.3976	2.50E-07	9.69E-05	PREDICTED: Erinaceus europaeus keratin-associated protein 9-3-like (LOC103120621), mRNA
Cluster-33503.37524	--	1.920	55.922	-4.8907	2.53E-07	9.77E-05	PREDICTED: Erinaceus europaeus sperm mitochondria associated cysteine rich protein (SMCP), mRNA

Cluster-33503.41615	--	594.840	81.249	2.8744	2.56E-07	9.83E-05	PREDICTED: Erinaceus europaeus coiled-coil glutamate rich protein 2 (CCER2), transcript variant X1, mRNA
Cluster-33503.52939	--	32.515	235.484	-2.8343	2.55E-07	9.83E-05	Atelerix albiventris clone LB4-464N6, complete sequence
Cluster-33503.132650	--	3.478	65.679	-4.3737	2.60E-07	9.97E-05	PREDICTED: Erinaceus europaeus calponin 1 (CNN1), transcript variant X2, mRNA
Cluster-33503.45911	--	79.403	792.055	-3.3249	2.78E-07	0.00010621	PREDICTED: Erinaceus europaeus cornifin (LOC107523341), mRNA
Cluster-33503.50145	--	67.018	642.968	-3.2724	2.80E-07	0.00010674	PREDICTED: Erinaceus europaeus keratin-associated protein 7-1 (LOC103122995), mRNA
Cluster-4275.0	--	0.000	30.693	-7.4534	2.87E-07	0.00010921	Gossypium barbadense EXA1 mRNA, complete cds
Cluster-33503.45652	--	144.269	1147.069	-2.9957	3.04E-07	0.00011566	PREDICTED: Erinaceus europaeus keratin-associated protein 9-3-like (LOC103120634), mRNA
Cluster-33503.147308	--	8.336	154.108	-4.2633	3.17E-07	0.00012007	PREDICTED: Erinaceus europaeus keratin-associated protein 8-1 (LOC107523100), mRNA
Cluster-33503.8716	--	0.000	29.614	-7.4025	3.20E-07	0.00012097	Atelerix albiventris clone LB4-141D24, complete sequence
Cluster-33503.27580	--	14.380	139.152	-3.2718	3.25E-07	0.00012261	PREDICTED: Equus caballus homeobox C12 (HOXC12), mRNA
Cluster-33503.165151	--	5.037	73.322	-3.8232	3.25E-07	0.00012268	PREDICTED: Odobenus rosmarus divergens engrailed homeobox 1 (EN1), mRNA
Cluster-33503.124815	--	31.926	278.391	-3.1435	3.36E-07	0.0001266	PREDICTED: Erinaceus europaeus keratin-associated protein 9-3-like (LOC103120621), mRNA
Cluster-33503.68991	--	0.660	52.941	-5.9768	3.54E-07	0.00013243	PREDICTED: Erinaceus europaeus keratin, type II cytoskeletal 6A (LOC103109181), mRNA
Cluster-33503.47054	--	9.294	101.115	-3.4984	3.69E-07	0.00013753	--

Cluster-33503.574	--	2.139	251.450	-6.9345	3.69E-07	0.00013753	PREDICTED: Erinaceus europaeus cornifin-B-like (LOC103125524), mRNA
Cluster-5146.0	--	0.000	39.509	-7.8225	3.69E-07	0.00013753	PREDICTED: Gossypium raimondii 1-aminocyclopropane-1-carboxylate oxidase (LOC105769639), mRNA
Cluster-33503.57732	--	184.564	1040.770	-2.497	3.73E-07	0.00013892	PREDICTED: Erinaceus europaeus keratin, type I cytoskeletal 14 (LOC103124740), mRNA
Cluster-5598.1	--	0.000	30.382	-7.4389	3.76E-07	0.00013984	Theobroma cacao genome assembly, chromosome: V
Cluster-33503.16758	--	12.841	159.443	-3.6374	3.88E-07	0.00014388	PREDICTED: Erinaceus europaeus neuropilin and tolloid like 2 (NETO2), mRNA
Cluster-33503.91004	--	164.532	1074.285	-2.7089	3.90E-07	0.00014456	--
Cluster-33503.28422	--	52.610	3.276	4.0608	4.05E-07	0.00014946	--
Cluster-5396.0	--	0.000	31.822	-7.5055	4.09E-07	0.00015033	Gossypium hirsutum gibberellin 20-oxidase 1 mRNA, complete cds
Cluster-33503.188425	--	0.000	30.792	-7.4562	4.17E-07	0.00015313	--
Cluster-33503.123956	--	66.126	3.243	4.3851	4.22E-07	0.00015456	PREDICTED: Peromyscus maniculatus bairdii keratin-associated protein 5-1-like (LOC102921526), mRNA
Cluster-74153.0	--	31.025	0.000	7.3585	4.24E-07	0.0001551	PREDICTED: Capsicum annuum photosystem II reaction center W protein, chloroplastic-like (LOC107843200), mRNA
Cluster-33503.14835	--	16.797	111.093	-2.7216	4.47E-07	0.00016332	PREDICTED: Erinaceus europaeus ankyrin repeat domain 31 (ANKRD31), mRNA
Cluster-33503.22827	--	396.839	2696.328	-2.7648	4.56E-07	0.00016595	PREDICTED: Galeopterus variegatus trichohyalin-like (LOC103594105), mRNA
Cluster-33503.417	--	14.576	122.100	-3.0685	4.82E-07	0.00017482	--
Cluster-33503.79751	--	18.677	169.330	-3.1616	5.02E-07	0.00018162	Atelerix albiventris clone LB4-226M9, complete sequence

Cluster-33503.73400	--	8.719	99.511	-3.5034	5.15E-07	0.00018637	PREDICTED: Erinaceus europaeus tropomyosin 2 (beta) (TPM2), transcript variant X3, mRNA
Cluster-33503.179812	--	12.297	97.037	-2.9673	5.25E-07	0.00018939	PREDICTED: Erinaceus europaeus tetratricopeptide repeat domain 29 (TTC29), mRNA
Cluster-33503.122270	--	0.721	56.718	-6.4577	5.30E-07	0.00019076	PREDICTED: Erinaceus europaeus keratin-associated protein 8-1 (LOC107523100), mRNA
Cluster-5020.0	--	0.000	28.316	-7.3366	5.67E-07	0.00020378	PREDICTED: Gossypium arboreum probable chalcone--flavonone isomerase 3 (LOC108457753), mRNA
Cluster-33503.64666	--	73.356	6925.988	-6.5596	5.74E-07	0.00020602	PREDICTED: Erinaceus europaeus cysteine-rich secretory protein 3-like (LOC103113737), mRNA
Cluster-33503.182832	--	14.878	165.233	-3.5063	6.15E-07	0.00021979	--
Cluster-33503.93403	--	3.220	54.150	-4.1644	6.46E-07	0.00023052	PREDICTED: Erinaceus europaeus tropomyosin 2 (beta) (TPM2), transcript variant X1, mRNA
Cluster-33503.136022	--	1.120	46.979	-5.5707	6.60E-07	0.00023456	PREDICTED: Erinaceus europaeus leiomodin 1 (LMOD1), mRNA
Cluster-33503.27939	--	109.402	10.425	3.3654	6.82E-07	0.00024177	--
Cluster-33503.47305	--	21.210	158.606	-2.9232	6.95E-07	0.00024588	PREDICTED: Erinaceus europaeus keratin-associated protein 9-3-like (LOC103120634), mRNA
Cluster-33503.46948	--	2.218	67.630	-5.0517	6.97E-07	0.00024631	PREDICTED: Erinaceus europaeus keratin-associated protein 8-1 (LOC107523100), mRNA
Cluster-33503.43814	--	88.687	728.359	-3.0451	7.05E-07	0.00024902	PREDICTED: Erinaceus europaeus keratin-associated protein 9-3-like (LOC103120634), mRNA
Cluster-33503.42609	--	4.779	69.587	-3.9485	7.45E-07	0.0002624	PREDICTED: Erinaceus europaeus keratin-associated protein 15-1 (LOC103118357), mRNA
Cluster-33503.76736	--	291.548	1336.015	-2.1977	7.62E-07	0.00026808	PREDICTED: Erinaceus europaeus myosin light chain 9 (MYL9), mRNA

Cluster-33503.130113	--	10.772	1650.981	-7.2678	7.79E-07	0.00027374	PREDICTED: Erinaceus europaeus keratin-associated protein 13-1-like (LOC103118345), mRNA
Cluster-5188.0	--	0.000	28.681	-7.3535	7.93E-07	0.00027809	Gossypium hirsutum clone CKE6-4A E6 gene, complete cds
Cluster-33503.123618	--	17.430	149.121	-3.1275	8.05E-07	0.00028195	PREDICTED: Erinaceus europaeus keratin-associated protein 7-1 (LOC103122995), mRNA
Cluster-33503.122268	--	0.400	51.691	-7.2395	8.74E-07	0.00030479	--
Cluster-33503.127496	--	15.952	182.604	-3.5325	9.07E-07	0.00031478	PREDICTED: Erinaceus europaeus cornifin (LOC107523341), mRNA
Cluster-33503.179008	--	0.000	35.097	-7.6445	9.33E-07	0.00032315	PREDICTED: Papio anubis keratin-associated protein 19-3 (LOC103882641), mRNA
Cluster-33503.134081	--	571.281	55.637	3.3599	9.62E-07	0.00033229	PREDICTED: Erinaceus europaeus T-cell immunoglobulin and mucin domain containing 4 (TIMD4), transcript variant X1, mRNA
Cluster-33503.130060	--	0.360	51.752	-7.2413	9.86E-07	0.00034022	PREDICTED: Erinaceus europaeus keratin-associated protein 8-1 (LOC107523100), mRNA
Cluster-33503.129790	--	93.645	627.874	-2.7519	1.01E-06	0.00034764	PREDICTED: Erinaceus europaeus keratin-associated protein 9-3-like (LOC103120621), mRNA
Cluster-33503.15509	--	20.551	157.963	-2.9324	1.02E-06	0.00035136	Atelerix albiventris clone LBNL4-89B6, complete sequence
Cluster-33503.62181	--	5.078	79.566	-4.041	1.05E-06	0.00036043	Atelerix albiventris clone LB4-108K18, complete sequence
Cluster-33503.45901	--	2.219	55.731	-4.674	1.08E-06	0.00037006	--
Cluster-33503.48729	--	1190.462	8573.064	-2.8488	1.21E-06	0.00041203	PREDICTED: Erinaceus europaeus keratin-associated protein 13-2-like (LOC103118344), mRNA
Cluster-4058.0	--	0.000	26.756	-7.2531	1.26E-06	0.00042865	PREDICTED: Gossypium hirsutum non-specific lipid transfer protein GPI-anchored 1-like (LOC107933394), mRNA

Cluster-42781.0	--	92.417	758.692	-3.0435	1.29E-06	0.00043656	PREDICTED: Erinaceus europaeus keratin-associated protein 13-2-like (LOC103118344), mRNA
Cluster-33503.178985	--	0.000	34.813	-7.633	1.29E-06	0.00043708	PREDICTED: Erinaceus europaeus keratin-associated protein 19-3-like (LOC103118358), mRNA
Cluster-4340.0	--	0.000	26.946	-7.2643	1.31E-06	0.00044202	PREDICTED: Gossypium arboreum anthocyanidin reductase (LOC108452981), mRNA
Cluster-33503.45896	--	10.438	115.940	-3.5062	1.37E-06	0.00046307	--
Cluster-33503.129665	--	3150.785	23063.060	-2.872	1.40E-06	0.00047062	PREDICTED: Erinaceus europaeus keratin-associated protein 9-3-like (LOC103120634), mRNA
Cluster-33503.70846	--	110.447	523.719	-2.2404	1.40E-06	0.00047158	PREDICTED: Erinaceus europaeus synaptopodin 2 (SYNPO2), mRNA
Cluster-33503.63695	--	3.699	47.750	-3.7481	1.54E-06	0.00051307	PREDICTED: Erinaceus europaeus uroplakin 2 (UPK2), transcript variant X2, mRNA
Cluster-33503.46878	--	115.791	1119.505	-3.2791	1.56E-06	0.00051966	PREDICTED: Erinaceus europaeus keratin, type I cuticular Ha4-like (LOC103124756), mRNA
Cluster-27086.0	--	27.824	0.000	7.1991	1.60E-06	0.00053271	PREDICTED: Capsicum annuum metallothionein-like protein type 2 B (LOC107842990), mRNA
Cluster-33503.81280	--	17.950	193.363	-3.4619	1.64E-06	0.00054606	PREDICTED: Erinaceus europaeus tropomyosin 2 (beta) (TPM2), transcript variant X1, mRNA
Cluster-33503.28423	--	42.464	2.078	4.3697	1.65E-06	0.00054794	--
Cluster-3934.0	--	0.000	26.114	-7.2194	1.66E-06	0.00055115	--
Cluster-33503.73399	--	8.476	91.504	-3.4427	1.73E-06	0.00057062	PREDICTED: Fukomys damarensis tropomyosin 2 (beta) (Tpm2), transcript variant X3, mRNA
Cluster-33503.125141	--	169.564	1247.835	-2.8835	1.73E-06	0.00057124	PREDICTED: Erinaceus europaeus keratin-associated protein 9-3-like (LOC103120634), mRNA
Cluster-33503.37531	--	14.294	124.213	-3.0962	1.76E-06	0.00058131	PREDICTED: Erinaceus europaeus sperm mitochondria associated cysteine rich protein (SMCP), mRNA

Cluster-33503.47094	--	999.812	6950.835	-2.798	1.77E-06	0.0005828	PREDICTED: Erinaceus europaeus keratin-associated protein 13-2-like (LOC103118344), mRNA
Cluster-33503.129688	--	9.514	87.792	-3.2584	1.80E-06	0.00059185	TPA_inf: Erinaceus europaeus gene for trappin-2, complete cds
Cluster-33503.124957	--	22.716	156.787	-2.8121	1.83E-06	0.00059962	--
Cluster-33503.46887	--	31.485	229.771	-2.8829	1.90E-06	0.00062044	--
Cluster-4136.0	--	0.000	23.767	-7.0858	1.92E-06	0.00062567	PREDICTED: Gossypium hirsutum uncharacterized LOC107899249 (LOC107899249), mRNA
Cluster-33503.50043	--	2845.105	17905.684	-2.6541	1.92E-06	0.00062688	PREDICTED: Erinaceus europaeus keratin-associated protein 9-3-like (LOC103120634), mRNA
Cluster-33503.34575	--	12.518	100.374	-2.9783	1.93E-06	0.00062814	PREDICTED: Sus scrofa uncharacterized LOC102162614 (LOC102162614), mRNA
Cluster-33503.128916	--	0.220	36.501	-6.7443	1.97E-06	0.00064045	Cyprinus carpio genome assembly common carp genome ,scaffold 000028897
Cluster-33503.43503	--	4.279	60.986	-3.865	2.01E-06	0.00065206	PREDICTED: Erinaceus europaeus keratin-associated protein 8-1 (LOC107523100), mRNA
Cluster-33503.679	--	18.489	167.247	-3.1861	2.13E-06	0.00068766	--
Cluster-33503.42562	--	0.980	40.960	-5.4448	2.22E-06	0.00071278	PREDICTED: Erinaceus europaeus keratin-associated protein 15-1 (LOC103118357), mRNA
Cluster-33503.131761	--	5.740	74.385	-3.6881	2.28E-06	0.00072902	PREDICTED: Erinaceus europaeus dentin matrix acidic phosphoprotein 1 (DMP1), transcript variant X2, mRNA
Cluster-33503.92574	--	146.793	983.315	-2.7436	2.29E-06	0.00073319	PREDICTED: Erinaceus europaeus claudin 4 (CLDN4), mRNA
Cluster-43360.0	--	2.478	50.790	-4.3677	2.40E-06	0.00076208	--
Cluster-26186.0	--	24.290	0.000	7.0054	2.48E-06	0.00078913	PREDICTED: Capsicum annuum oxygen-evolving enhancer protein 3-2, chloroplastic (LOC107859535), mRNA

Cluster-33503.122755	--	510.895	4081.819	-2.9995	2.51E-06	0.00079759	PREDICTED: Erinaceus europaeus keratin, type I cuticular Ha4-like (LOC103124756), mRNA
Cluster-33503.132647	--	3.338	50.162	-4.026	2.53E-06	0.00080221	PREDICTED: Erinaceus europaeus calponin 1 (CNN1), transcript variant X1, mRNA
Cluster-33503.8464	--	2.718	53.039	-4.4298	2.57E-06	0.00081359	PREDICTED: Erinaceus europaeus keratin-associated protein 13-2 (LOC103118343), mRNA
Cluster-33503.123819	--	58.230	303.433	-2.3916	2.62E-06	0.0008259	PREDICTED: Erinaceus europaeus keratin-associated protein 4-11-like (LOC103120636), transcript variant X1, mRNA
Cluster-33503.122695	--	135.245	1354.176	-3.3282	2.79E-06	0.00087541	--
Cluster-33503.124426	--	67.304	3.642	4.244	2.88E-06	0.0009006	PREDICTED: Erinaceus europaeus keratin-associated protein 9-2 (LOC103124750), mRNA
Cluster-33503.126251	--	1.959	50.995	-4.8678	2.90E-06	0.00090789	--
Cluster-33503.8599	--	0.000	97.496	-9.1243	2.91E-06	0.00090986	PREDICTED: Erinaceus europaeus keratin-associated protein 19-2-like (LOC103122997), mRNA
Cluster-33503.43410	--	5.238	62.020	-3.5669	2.97E-06	0.00092628	--
Cluster-33503.127109	--	2.838	43.024	-3.9446	3.02E-06	0.00093912	PREDICTED: Erinaceus europaeus keratin-associated protein 3-3 (LOC103120617), mRNA
Cluster-33503.160902	--	0.799	42.568	-6.008	3.13E-06	0.00096763	PREDICTED: Erinaceus europaeus keratin-associated protein 19-2-like (LOC103118359), mRNA
Cluster-33503.27985	--	183.732	873.144	-2.2522	3.12E-06	0.00096763	PREDICTED: Erinaceus europaeus family with sequence similarity 26 member D (FAM26D), mRNA
Cluster-67026.0	--	22.615	0.000	6.9065	3.30E-06	0.0010199	PREDICTED: Solanum tuberosum ferredoxin--NADP reductase, leaf-type isozyme, chloroplastic (LOC102591399), mRNA
Cluster-33503.48401	--	86.160	592.107	-2.7887	3.42E-06	0.0010517	PREDICTED: Erinaceus europaeus keratin-associated protein 9-3-like (LOC103120634), mRNA

Cluster-99592.0	--	23.790	0.000	6.971	3.51E-06	0.0010782	PREDICTED: Capsicum annuum chlorophyll a-b binding protein 8, chloroplastic (LOC107845349), mRNA
Cluster-33503.180451	--	1.999	37.345	-4.2947	3.80E-06	0.0011622	Atelerix albiventris clone LB4-108K18, complete sequence
Cluster-33503.9348	--	10.210	5682.939	-9.1308	4.00E-06	0.0012164	PREDICTED: Erinaceus europaeus keratin-associated protein 19-2-like (LOC103118359), mRNA
Cluster-33503.122696	--	56.519	486.850	-3.1159	4.07E-06	0.0012339	--
Cluster-4984.0	--	0.000	23.766	-7.0813	4.09E-06	0.001237	Homo sapiens chromosome 13q34 schizophrenia region contig 1 complete sequence
Cluster-33503.63663	--	324.810	1248.257	-1.9411	4.17E-06	0.0012595	PREDICTED: Erinaceus europaeus gap junction protein alpha 1 (GJA1), mRNA
Cluster-33503.139468	--	191.524	895.076	-2.2236	4.24E-06	0.0012774	PREDICTED: Erinaceus europaeus hephaestin like 1 (HEPHL1), mRNA
Cluster-33503.45895	--	2.899	56.921	-4.3839	4.25E-06	0.00128	PREDICTED: Erinaceus europaeus cornifin (LOC107523341), mRNA
Cluster-33503.6812	--	5.401	71.148	-3.7359	4.27E-06	0.0012828	Atelerix albiventris clone LB4-244I20, complete sequence
Cluster-42136.0	--	24.545	0.000	7.0172	4.28E-06	0.0012836	PREDICTED: Capsicum annuum cytochrome b6-f complex iron-sulfur subunit, chloroplastic (LOC107863280), mRNA
Cluster-33503.178558	--	0.400	38.650	-6.8193	4.34E-06	0.0013014	PREDICTED: Erinaceus europaeus keratin-associated protein 19-2-like (LOC103118359), mRNA
Cluster-33503.28381	--	3.219	80.669	-4.5497	4.43E-06	0.0013252	Homo sapiens BAC clone CH17-203B2 from chromosome 1, complete sequence
Cluster-33503.189441	--	1.159	37.962	-5.2511	4.45E-06	0.0013287	PREDICTED: Erinaceus europaeus melan-A (MLANA), mRNA

Cluster-33503.7891	--	11.193	103.715	-3.2416	4.50E-06	0.0013398	PREDICTED: Erinaceus europaeus keratin-associated protein 9-3-like (LOC103120634), mRNA
Cluster-33503.44093	--	52.646	303.942	-2.5221	4.79E-06	0.0014247	PREDICTED: Erinaceus europaeus phospholipase C beta 4 (PLCB4), transcript variant X3, mRNA
Cluster-33503.73581	--	44.809	194.165	-2.1177	4.83E-06	0.0014336	--
Cluster-33503.122238	--	86.469	424.408	-2.2996	4.86E-06	0.0014421	--
Cluster-33503.130147	--	0.220	38.368	-6.8154	4.87E-06	0.0014433	PREDICTED: Erinaceus europaeus keratin-associated protein 8-1 (LOC107523100), mRNA
Cluster-33503.122267	--	20.716	137.546	-2.7439	5.04E-06	0.0014859	PREDICTED: Erinaceus europaeus keratin, type I cytoskeletal 14 (LOC103124740), mRNA
Cluster-33503.92786	--	10.200	96.556	-3.1984	5.05E-06	0.0014859	Atelerix albiventris clone LB4-477I10, complete sequence
Cluster-33503.45807	--	56.787	4.414	3.6397	5.06E-06	0.0014899	PREDICTED: Erinaceus europaeus keratin-associated protein 12-3-like (LOC103128196), mRNA
Cluster-33503.1461	--	0.980	35.645	-5.2461	5.11E-06	0.0015023	--
Cluster-33503.18168	--	19.400	15080.803	-9.6073	5.13E-06	0.0015074	PREDICTED: Erinaceus europaeus keratin-associated protein 19-2-like (LOC103118359), mRNA
Cluster-33503.128015	--	7.896	77.014	-3.3052	5.15E-06	0.0015095	PREDICTED: Erinaceus europaeus keratin, type II cuticular Hb1 (LOC103109277), mRNA
Cluster-33503.28686	--	2.600	45.867	-4.2537	5.27E-06	0.0015438	PREDICTED: Erinaceus europaeus REC114 meiotic recombination protein (REC114), partial mRNA
Cluster-33503.68990	--	0.000	21.144	-6.9164	5.31E-06	0.0015527	PREDICTED: Erinaceus europaeus keratin, type II cytoskeletal 6A-like (LOC103109273), mRNA
Cluster-33503.125140	--	24.072	148.537	-2.6393	5.69E-06	0.0016552	PREDICTED: Erinaceus europaeus keratin-associated protein 9-3-like (LOC103120634), mRNA
Cluster-49809.0	--	22.389	0.000	6.8846	5.90E-06	0.0017157	Capsicum annuum acetolactate synthase 2, chloroplastic (LOC107853330), mRNA

Cluster-33503.184303	--	34.375	162.153	-2.2299	5.98E-06	0.0017367	PREDICTED: Erinaceus europaeus synaptotagmin 17 (SYT17), transcript variant X2, mRNA
Cluster-33503.44582	--	96.016	9394.022	-6.6135	6.12E-06	0.0017741	PREDICTED: Erinaceus europaeus keratin-associated protein 19-3-like (LOC103118358), mRNA
Cluster-23173.1	--	0.220	33.337	-6.6117	6.25E-06	0.0018102	Ovis canadensis canadensis isolate 43U chromosome 2 sequence
Cluster-33503.5497	--	20.793	118.521	-2.5185	6.34E-06	0.0018333	Atelerix albiventris clone LBNL4-89B6, complete sequence
Cluster-33503.126485	--	1171.875	4124.500	-1.8153	6.49E-06	0.0018715	PREDICTED: Erinaceus europaeus keratin, type I cytoskeletal 25 (LOC103120609), mRNA
Cluster-33503.66180	--	19.438	116.060	-2.5933	6.62E-06	0.0019072	PREDICTED: Erinaceus europaeus smoothelin (SMTN), transcript variant X2, mRNA
Cluster-33503.93220	--	57.144	305.147	-2.4295	6.67E-06	0.0019203	PREDICTED: Erinaceus europaeus myosin light chain 9 (MYL9), mRNA
Cluster-100792.0	--	23.725	0.000	6.9651	6.80E-06	0.0019514	PREDICTED: Capsicum annuum chlorophyll a-b binding protein 7, chloroplastic (LOC107838850), mRNA
Cluster-96981.0	--	26.329	0.000	7.1282	6.86E-06	0.001968	PREDICTED: Erinaceus europaeus HEPACAM family member 2 (HEPACAM2), transcript variant X2, mRNA
Cluster-33503.182413	--	0.000	21.738	-6.9547	6.95E-06	0.0019858	PREDICTED: Gossypium hirsutum metallothionein-like protein 2 (LOC107958011), mRNA
Cluster-43855.0	--	56.655	5.287	3.4377	6.94E-06	0.0019858	PREDICTED: Erinaceus europaeus otogelin (OTOG), mRNA
Cluster-33503.177659	--	61.437	53085.231	-9.7564	7.10E-06	0.0020242	PREDICTED: Erinaceus europaeus keratin-associated protein 19-2-like (LOC103118359), mRNA
Cluster-33503.65293	--	486.668	2562.702	-2.3964	7.34E-06	0.0020869	PREDICTED: Erinaceus europaeus sestrin 2 (SESN2), mRNA

Cluster-33503.51253	--	461.195	1874.635	-2.0241	7.53E-06	0.0021358	PREDICTED: Erinaceus europaeus junction mediating and regulatory protein, p53 cofactor (JMY), mRNA
Cluster-33503.186074	--	1.859	45.294	-4.6175	7.59E-06	0.0021492	PREDICTED: Erinaceus europaeus leucine rich repeat containing 72 (LRRC72), mRNA
Cluster-33503.22437	--	4.759	49.088	-3.3832	7.60E-06	0.0021492	--
Cluster-33503.91006	--	71.624	362.844	-2.3372	7.69E-06	0.0021694	--
Cluster-33503.132632	--	1.379	31.701	-4.6309	7.75E-06	0.002183	PREDICTED: Erinaceus europaeus calponin 1 (CNN1), transcript variant X2, mRNA
Cluster-33503.91003	--	25.199	160.253	-2.6671	7.80E-06	0.0021927	--
Cluster-33503.86069	--	93.132	615.071	-2.7258	8.12E-06	0.0022745	Erinaceus europaeus, clone XX-29460857F24, complete sequence
Cluster-54624.0	--	20.892	0.000	6.7852	8.14E-06	0.0022789	Capsicum annuum ketol-acid reductoisomerase, chloroplastic-like (LOC107856177), mRNA
Cluster-33503.123825	--	23.298	136.462	-2.5716	8.22E-06	0.0022956	PREDICTED: Erinaceus europaeus keratin-associated protein 4-11-like (LOC103120636), transcript variant X1, mRNA
Cluster-33503.127285	--	53.764	2.571	4.4304	8.22E-06	0.0022956	--
Cluster-5327.0	--	0.000	20.909	-6.8968	8.30E-06	0.0023135	Gossypium hirsutum partial mRNA for high-glycine tyrosine keratin-like protein (xl3 gene)
Cluster-33503.126798	--	81.924	444.193	-2.4351	8.45E-06	0.0023507	PREDICTED: Equus przewalskii dystrobrevin, alpha (DTNA), transcript variant X10, mRNA
Cluster-33503.150592	--	0.880	34.128	-4.9676	8.67E-06	0.0024093	PREDICTED: Erinaceus europaeus keratin, type I cytoskeletal 16 (LOC103124739), mRNA
Cluster-33503.67453	--	377.462	1339.223	-1.8265	8.86E-06	0.0024592	PREDICTED: Erinaceus europaeus family with sequence similarity 83 member G (FAM83G), mRNA
Cluster-33503.28382	--	1.719	51.290	-4.7531	8.88E-06	0.0024637	--

Cluster-27774.0	--	33.773	0.399	6.534	9.05E-06	0.0025063	PREDICTED: Capsicum annuum protein SENESCENCE-ASSOCIATED GENE 21, mitochondrial-like (LOC107864041), mRNA
Cluster-33503.179516	--	1.598	185.238	-6.9622	9.18E-06	0.0025404	PREDICTED: Erinaceus europaeus keratin, type II cytoskeletal 6A-like (LOC103109273), mRNA
Cluster-30792.0	--	20.850	0.000	6.786	9.66E-06	0.0026698	PREDICTED: Capsicum annuum histone H1 (LOC107859896), mRNA
Cluster-33503.122269	--	0.220	30.348	-6.4767	9.76E-06	0.0026925	--
Cluster-33503.130405	--	127.846	10189.421	-6.3176	1.03E-05	0.0028286	PREDICTED: Erinaceus europaeus keratin-associated protein 19-2-like (LOC103118359), mRNA
Cluster-33503.70358	--	6.898	59.146	-3.1287	1.04E-05	0.0028584	Atelerix albiventris clone LB4-341B4, complete sequence
Cluster-33503.98393	--	0.220	42.513	-6.9669	1.05E-05	0.002894	--
Cluster-33503.44573	--	387.522	53291.147	-7.1036	1.06E-05	0.002899	PREDICTED: Erinaceus europaeus keratin-associated protein 19-3-like (LOC103118358), mRNA
Cluster-33503.126024	--	552.692	2048.348	-1.8894	1.07E-05	0.0029339	PREDICTED: Erinaceus europaeus junction mediating and regulatory protein, p53 cofactor (JMY), mRNA
Cluster-33503.43351	--	8216.509	62412.834	-2.9253	1.08E-05	0.0029643	--
Cluster-33503.26868	--	84.754	12.491	2.7437	1.10E-05	0.003009	Atelerix albiventris clone LB4-234A16, complete sequence
Cluster-33503.191735	--	0.000	19.221	-6.7893	1.12E-05	0.0030414	Atelerix albiventris clone LB4-464N6, complete sequence
Cluster-33503.140433	--	137.271	495.945	-1.8525	1.13E-05	0.0030781	PREDICTED: Erinaceus europaeus nucleic acid binding protein 1 (NABP1), mRNA
Cluster-33503.56940	--	165.291	18.485	3.1705	1.15E-05	0.0031371	PREDICTED: Erinaceus europaeus mesothelin (MSLN), mRNA
Cluster-33503.182834	--	3.698	47.914	-3.7938	1.16E-05	0.003158	Atelerix albiventris clone LB4-4E15, complete sequence

Cluster-33503.50040	--	8.019	61.691	-2.9591	1.19E-05	0.0032305	Ovis canadensis canadensis isolate 43U chromosome 11 sequence
Cluster-33503.50046	--	36.949	279.579	-2.9356	1.23E-05	0.0033311	PREDICTED: Erinaceus europaeus keratin-associated protein 9-3-like (LOC103120621), mRNA
Cluster-33503.48809	--	71.973	2510.494	-5.1275	1.23E-05	0.0033321	PREDICTED: Erinaceus europaeus keratin-associated protein 8-1 (LOC107523100), mRNA
Cluster-33503.98062	--	76.018	335.121	-2.1463	1.24E-05	0.0033479	PREDICTED: Canis lupus familiaris filamin A, alpha (FLNA), mRNA
Cluster-33503.6882	--	1.380	45.070	-4.9492	1.24E-05	0.0033503	PREDICTED: Erinaceus europaeus transmembrane protein 252 (TMEM252), mRNA
Cluster-94462.0	--	0.400	28.235	-6.3713	1.28E-05	0.0034496	--
Cluster-33503.47173	--	3.120	78.571	-4.659	1.29E-05	0.0034535	Equus caballus clone FoalT25 immmunoglobulin lambda light chain variable region (IGL) mRNA, partial cds
Cluster-33503.158959	--	1.600	36.459	-4.3983	1.30E-05	0.0034942	--
Cluster-33503.129774	--	26.924	203.129	-2.94	1.34E-05	0.003581	PREDICTED: Erinaceus europaeus keratin-associated protein 7-1 (LOC103122995), mRNA
Cluster-33503.187092	--	2.837	254.978	-6.5289	1.35E-05	0.0035989	Rhesus Macaque BAC CH250-204A18 () complete sequence
Cluster-33503.45745	--	5.559	66.381	-3.5805	1.35E-05	0.0035989	Atelerix albiventris clone LB4-307O5, complete sequence
Cluster-33503.36826	--	198.771	1295.848	-2.7023	1.36E-05	0.0036203	PREDICTED: Erinaceus europaeus heparin binding EGF like growth factor (HBEGF), mRNA
Cluster-33503.124538	--	169.095	865.241	-2.3578	1.37E-05	0.0036456	--
Cluster-33503.127387	--	7.218	67.037	-3.2556	1.38E-05	0.0036599	Erinaceus europaeus, clone XX-29441557E23, complete sequence
Cluster-33503.129166	--	4.740	51.932	-3.5316	1.38E-05	0.0036599	--

Cluster-33503.27498	--	0.220	28.412	-6.3812	1.41E-05	0.0037314	Atelerix albiventris clone LBNL4-89B6, complete sequence
Cluster-33503.179317	--	10.992	1026.253	-6.5606	1.45E-05	0.0038243	PREDICTED: Erinaceus europaeus keratin, type II cytoskeletal 6A-like (LOC103109273), mRNA
Cluster-33503.44506	--	2.539	36.101	-3.946	1.48E-05	0.0039092	--
Cluster-55734.0	--	0.000	18.677	-6.746	1.49E-05	0.0039449	Atelerix albiventris clone LB4-464N6, complete sequence
Cluster-33503.47663	--	3.220	44.722	-3.8773	1.53E-05	0.0040326	--
Cluster-33503.180133	--	2.978	51.083	-4.203	1.58E-05	0.0041559	Homo sapiens FOSMID clone ABC8-40986100K1 from chromosome unknown, complete sequence
Cluster-33503.124541	--	101.366	507.876	-2.3321	1.61E-05	0.0042172	PREDICTED: Erinaceus europaeus keratin, high-sulfur matrix protein, B2C-like (LOC107522941), mRNA
Cluster-33503.129411	--	0.000	78.674	-8.8149	1.66E-05	0.0043332	PREDICTED: Erinaceus europaeus keratin-associated protein 19-2-like (LOC103118359), mRNA
Cluster-33503.44566	--	0.000	18.130	-6.6948	1.66E-05	0.0043409	Pan troglodytes chromosome 22 clone:RP43-082J09, map 22, complete sequences
Cluster-33503.126249	--	3.917	71.619	-4.2863	1.67E-05	0.0043625	--
Cluster-33503.77988	--	0.660	37.775	-5.5194	1.67E-05	0.0043625	Atelerix albiventris clone LB4-307O5, complete sequence
Cluster-5149.2	--	0.000	18.320	-6.7074	1.67E-05	0.0043625	PREDICTED: Gossypium arboreum CASP-like protein F16 (LOC108483803), mRNA
Cluster-29962.0	--	18.813	0.000	6.64	1.68E-05	0.004382	PREDICTED: Capsicum annuum auxin-repressed 12.5 kDa protein (LOC107859358), transcript variant X1, mRNA
Cluster-33503.26589	--	145.397	637.719	-2.1366	1.69E-05	0.0043824	PREDICTED: Erinaceus europaeus keratin, type I cytoskeletal 14 (LOC103124740), mRNA
Cluster-33503.15887	--	0.840	39.750	-5.4862	1.72E-05	0.0044442	Mus musculus BAC clone RP23-127O4 from 12, complete sequence

Cluster-33503.179009	--	0.000	205.327	-10.199	1.71E-05	0.0044442	PREDICTED: Erinaceus europaeus keratin-associated protein 19-2-like (LOC103118359), mRNA
Cluster-33503.47209	--	76.166	352.932	-2.2202	1.73E-05	0.0044785	PREDICTED: Erinaceus europaeus keratin-associated protein 4-11-like (LOC103120636), transcript variant X1, mRNA
Cluster-33503.44578	--	34.372	4664.330	-7.086	1.73E-05	0.0044811	PREDICTED: Erinaceus europaeus keratin-associated protein 19-2-like (LOC103118359), mRNA
Cluster-33503.122846	--	0.220	35.276	-6.6944	1.76E-05	0.0045415	PREDICTED: Erinaceus europaeus keratin-associated protein 8-1 (LOC107523100), mRNA
Cluster-33503.178218	--	13.707	1859.773	-7.0929	1.79E-05	0.0045958	PREDICTED: Erinaceus europaeus keratin-associated protein 19-3-like (LOC103118358), mRNA
Cluster-33503.161586	--	4.038	47.724	-3.5806	1.79E-05	0.0046019	Ovis canadensis canadensis isolate 43U chromosome 3 sequence
Cluster-33503.45170	--	20.229	134.462	-2.759	1.79E-05	0.0046019	Atelerix albiventris clone LB4-495B20, complete sequence
Cluster-33503.124842	--	433.757	1680.617	-1.9547	1.82E-05	0.0046749	PREDICTED: Erinaceus europaeus ring finger protein 39 (RNF39), mRNA
Cluster-33503.177719	--	4.216	416.399	-6.6623	1.88E-05	0.0048083	PREDICTED: Erinaceus europaeus keratin-associated protein 19-3-like (LOC103118358), mRNA
Cluster-33503.28420	--	154.871	15.521	3.3199	1.91E-05	0.0048711	--
Cluster-111387.0	--	78.636	10.253	2.934	1.96E-05	0.0049974	PREDICTED: Erinaceus europaeus WAP four-disulfide core domain 2 (WFDC2), mRNA
Cluster-33503.131450	--	1374.983	5555.203	-2.0143	1.96E-05	0.004998	PREDICTED: Erinaceus europaeus hephaestin like 1 (HEPHL1), mRNA
Cluster-33503.32810	--	35.081	205.078	-2.5641	1.98E-05	0.0050386	PREDICTED: Erinaceus europaeus G protein-coupled receptor class C group 5 member D (GPRC5D), mRNA
Cluster-33503.87788	--	560.508	2133.631	-1.9293	1.98E-05	0.0050426	PREDICTED: Erinaceus europaeus jun B proto-oncogene (JUNB), mRNA

Cluster-51585.0	--	21.065	195.611	-3.2277	2.02E-05	0.0051318	--
Cluster-73306.0	--	18.632	0.000	6.6184	2.03E-05	0.0051373	PREDICTED: Capsicum annuum oxygen-evolving enhancer protein 1, chloroplastic (LOC107860359), mRNA
Cluster-33503.125924	--	30.601	898.211	-4.8825	2.05E-05	0.0051932	--
Cluster-33503.92573	--	37.169	206.802	-2.4796	2.06E-05	0.0051987	PREDICTED: Erinaceus europaeus claudin 4 (CLDN4), mRNA
Cluster-33503.132563	--	138.759	598.472	-2.1133	2.06E-05	0.0052123	PREDICTED: Erinaceus europaeus homeobox D9 (HOXD9), mRNA
Cluster-33503.123816	--	811.026	3093.554	-1.9324	2.08E-05	0.0052499	PREDICTED: Erinaceus europaeus keratin-associated protein 4-11-like (LOC103120636), transcript variant X1, mRNA
Cluster-27939.0	--	18.950	0.000	6.6456	2.14E-05	0.0054007	PREDICTED: Capsicum annuum chlorophyll a-b binding protein CP29.1, chloroplastic-like (LOC107842738), mRNA
Cluster-33503.104508	--	55.709	4.993	3.4505	2.18E-05	0.0054734	PREDICTED: Equus asinus selectin L (SELL), transcript variant X2, mRNA
Cluster-33503.177934	--	2.397	963.692	-8.6935	2.20E-05	0.005496	PREDICTED: Erinaceus europaeus keratin-associated protein 19-2-like (LOC103122997), mRNA
Cluster-53801.0	--	19.528	0.000	6.6869	2.20E-05	0.0055015	PREDICTED: Capsicum annuum thiamine thiazole synthase 1, chloroplastic (LOC107856341), mRNA
Cluster-33503.61032	--	139.028	530.162	-1.9286	2.28E-05	0.0056746	Atelerix albiventris clone LB4-197A24, complete sequence
Cluster-33503.37759	--	24.034	132.517	-2.4611	2.34E-05	0.0058234	PREDICTED: Erinaceus europaeus patched domain containing 1 (PTCHD1), mRNA
Cluster-33503.28910	--	72.280	311.981	-2.1056	2.35E-05	0.0058248	Atelerix albiventris clone LB4-344F17, complete sequence

Cluster-33503.123836	--	8.897	60.276	-2.789	2.37E-05	0.0058755	PREDICTED: Erinaceus europaeus keratin-associated protein 4-11-like (LOC103120636), transcript variant X1, mRNA
Cluster-33503.45893	--	3.598	49.898	-3.8253	2.40E-05	0.0059529	PREDICTED: Erinaceus europaeus cornifin (LOC107523341), mRNA
Cluster-33503.57670	--	9.797	75.876	-2.982	2.44E-05	0.0060317	--
Cluster-33503.117767	--	421.352	1433.812	-1.7677	2.44E-05	0.0060401	PREDICTED: Erinaceus europaeus absent in melanoma 1 (AIM1), mRNA
Cluster-33503.124888	--	15.433	102.697	-2.7607	2.55E-05	0.0062752	PREDICTED: Erinaceus europaeus keratin-associated protein 9-3-like (LOC103120634), mRNA
Cluster-33503.44478	--	5.563	64.662	-3.5572	2.66E-05	0.0065458	PREDICTED: Erinaceus europaeus desmin (DES), mRNA
Cluster-33503.127111	--	12.836	88.367	-2.8151	2.66E-05	0.0065471	Atelerix albiventris clone LB4-234A16, complete sequence
Cluster-33503.123979	--	17.432	95.668	-2.4598	2.69E-05	0.0066094	PREDICTED: Canis lupus familiaris keratin 35, type I (KRT35), mRNA
Cluster-33503.57738	--	230.911	1196.793	-2.3761	2.71E-05	0.0066346	PREDICTED: Erinaceus europaeus keratin, type I cytoskeletal 14 (LOC103124740), mRNA
Cluster-33503.125489	--	0.840	26.813	-4.9138	2.88E-05	0.007034	--
Cluster-26350.0	--	18.868	0.000	6.636	2.95E-05	0.0071879	PREDICTED: Capsicum annuum photosystem I reaction center subunit XI, chloroplastic-like (LOC107875373), mRNA
Cluster-33503.123024	--	9.135	64.500	-2.8354	2.96E-05	0.0072051	PREDICTED: Erinaceus europaeus keratin, type II cuticular Hb5 (LOC103109186), mRNA
Cluster-33503.64629	--	116.522	591.857	-2.3448	2.96E-05	0.007219	PREDICTED: Vicugna pacos cysteine-rich secretory protein LCCL domain containing 2 (CRISPLD2), mRNA

Cluster-99669.0	--	17.553	0.000	6.534	2.97E-05	0.0072265	PREDICTED: Capsicum annuum oxygen-evolving enhancer protein 1, chloroplastic (LOC107858690), mRNA
Cluster-33503.45543	--	5.438	58.192	-3.4835	3.00E-05	0.0072969	Atelerix albiventris clone LB4-108K18, complete sequence
Cluster-29280.0	--	18.509	0.000	6.6095	3.04E-05	0.0073819	PREDICTED: Capsicum annuum serine--glyoxylate aminotransferase (LOC107850292), transcript variant X1, mRNA
Cluster-33503.49067	--	23600.597	578523.251	-4.6155	3.04E-05	0.0073831	PREDICTED: Erinaceus europaeus keratin-associated protein 8-1 (LOC107523100), mRNA
Cluster-33503.38365	--	0.000	16.281	-6.5405	3.12E-05	0.0075595	PREDICTED: Erinaceus europaeus calponin 1 (CNN1), transcript variant X1, mRNA
Cluster-33503.179504	--	0.000	21.850	-6.9602	3.13E-05	0.0075805	PREDICTED: Erinaceus europaeus keratin-associated protein 19-2-like (LOC103118359), mRNA
Cluster-33503.182476	--	1.100	39.641	-4.911	3.14E-05	0.0076026	Atelerix albiventris clone LB4-197A24, complete sequence
Cluster-33503.30170	--	420.139	102.341	2.0389	3.19E-05	0.0076716	PREDICTED: Erinaceus europaeus T-box 18 (TBX18), mRNA
Cluster-33503.129410	--	4.835	322.522	-6.0912	3.19E-05	0.0076837	PREDICTED: Erinaceus europaeus keratin-associated protein 19-2-like (LOC103118359), mRNA
Cluster-82023.0	--	0.000	16.843	-6.5867	3.49E-05	0.0083826	PREDICTED: Gossypium arboreum translationally-controlled tumor protein homolog (LOC108483654), mRNA
Cluster-33503.127081	--	16.208	534.866	-5.0544	3.51E-05	0.0084381	PREDICTED: Erinaceus europaeus keratin-associated protein 19-5-like (LOC103122993), mRNA
Cluster-33503.127150	--	889.104	3197.453	-1.846	3.52E-05	0.0084389	PREDICTED: Balaenoptera acutorostrata scammoni tensin 1 (TNS1), transcript variant X1, mRNA

Cluster-30691.0	--	16.760	0.000	6.4706	3.53E-05	0.0084633	PREDICTED: Capsicum annuum protein TAP1-like (LOC107845848), mRNA
Cluster-33503.57749	--	89.388	428.015	-2.2629	3.54E-05	0.008479	PREDICTED: Erinaceus europaeus keratin, type I cytoskeletal 14 (LOC103124740), mRNA
Cluster-33503.55894	--	968.163	3230.415	-1.7383	3.56E-05	0.0085168	PREDICTED: Erinaceus europaeus absent in melanoma 1 (AIM1), mRNA
Cluster-33503.127669	--	612.484	2127.169	-1.7965	3.57E-05	0.0085235	PREDICTED: Erinaceus europaeus EF-hand domain family member D1 (EFHD1), mRNA
Cluster-33503.9135	--	2.358	49.033	-4.507	3.75E-05	0.0089501	--
Cluster-33503.41723	--	4.038	39.601	-3.3104	3.80E-05	0.0090459	TPA_inf: Erinaceus europaeus gene for trappin-2, complete cds
Cluster-33503.6802	--	1.019	30.912	-5.0169	3.81E-05	0.0090631	--
Cluster-33503.169199	--	0.000	16.612	-6.5673	3.85E-05	0.0091484	--
Cluster-33503.66189	--	47.663	275.105	-2.5185	3.86E-05	0.0091544	PREDICTED: Oryctolagus cuniculus platelet derived growth factor subunit B (PDGFB), mRNA
Cluster-33503.44564	--	140.832	12286.630	-6.4478	3.88E-05	0.0092014	PREDICTED: Erinaceus europaeus keratin-associated protein 19-3-like (LOC103118358), mRNA
Cluster-33503.8393	--	5.936	53.257	-3.1761	3.91E-05	0.0092472	PREDICTED: Erinaceus europaeus serpin B13 (LOC103114269), transcript variant X1, mRNA
Cluster-33503.130114	--	34.255	6130.346	-7.4841	3.96E-05	0.0093713	PREDICTED: Erinaceus europaeus keratin-associated protein 13-1-like (LOC103118345), mRNA
Cluster-33503.48161	--	1384.309	292.201	2.2453	4.10E-05	0.0096801	PREDICTED: Erinaceus europaeus keratin-associated protein 9-4-like (LOC107523731), partial mRNA
Cluster-33503.49215	--	89.741	370.275	-2.0502	4.16E-05	0.0097925	PREDICTED: Erinaceus europaeus trichohyalin (TCHH), mRNA
Cluster-33503.53995	--	36.520	183.269	-2.3129	4.16E-05	0.0097925	Atelerix albiventris clone LB4-234A16, complete sequence

Cluster-4576.0	--	0.000	17.471	-6.6409	4.23E-05	0.0099296	PREDICTED: Gossypium hirsutum granule-bound starch synthase 1, chloroplastic/amyloplastic-like (LOC107911823), mRNA
Cluster-33503.46542	--	409.154	1688.979	-2.0463	4.28E-05	0.010034	PREDICTED: Erinaceus europaeus plakophilin 1 (PKP1), mRNA
Cluster-33503.123839	--	437.191	1886.743	-2.1113	4.34E-05	0.010175	PREDICTED: Erinaceus europaeus keratin-associated protein 4-11-like (LOC103120636), transcript variant X1, mRNA
Cluster-33503.78619	--	776.179	2860.205	-1.8825	4.39E-05	0.010278	Erinaceus europaeus, clone XX-29994857A5, complete sequence
Cluster-33503.124216	--	6.676	88.014	-3.7712	4.49E-05	0.010484	PREDICTED: Physeter catodon cuticle protein 64-like (LOC102975728), mRNA
Cluster-33503.78630	--	912.530	3292.608	-1.852	4.64E-05	0.010818	--
Cluster-33503.127777	--	28.438	118.463	-2.0576	4.66E-05	0.010846	--
Cluster-33503.157918	--	1.960	28.180	-3.8959	4.66E-05	0.010846	--
Cluster-33503.57719	--	11.952	87.532	-2.9088	4.67E-05	0.010852	PREDICTED: Erinaceus europaeus keratin, type I cytoskeletal 17 (LOC103124738), mRNA
Cluster-44834.0	--	30.631	1.218	4.5732	4.73E-05	0.010993	PREDICTED: Erinaceus europaeus ATP/GTP binding protein-like 4 (AGBL4), mRNA
Cluster-33503.106955	--	22.637	104.797	-2.2099	4.79E-05	0.011111	PREDICTED: Erinaceus europaeus carbohydrate sulfotransferase 1 (CHST1), mRNA
Cluster-33503.108534	--	5.899	60.137	-3.4124	4.79E-05	0.011111	Atelerix albiventris clone LB4-443C20, complete sequence
Cluster-33503.154295	--	0.000	18.002	-6.6803	4.80E-05	0.011111	Atelerix albiventris clone LB4-130D24, complete sequence
Cluster-33503.49221	--	16014.197	59309.214	-1.8889	4.81E-05	0.011141	PREDICTED: Erinaceus europaeus trichohyalin (TCHH), mRNA

Cluster-33503.5692	--	4.820	55.053	-3.4095	4.83E-05	0.011173	PREDICTED: Rousettus aegyptiacus collagen type XIX alpha 1 (COL19A1), mRNA
Cluster-33503.132629	--	1.199	28.877	-4.8186	4.85E-05	0.011197	--
Cluster-33503.9349	--	3.996	1925.086	-8.9346	4.87E-05	0.01125	PREDICTED: Erinaceus europaeus keratin-associated protein 19-3-like (LOC103118358), mRNA
Cluster-33503.165463	--	49.783	245.374	-2.2877	4.88E-05	0.011268	--
Cluster-33503.54454	--	0.360	32.593	-6.5857	4.91E-05	0.011323	Atelerix albiventris clone LB4-112D1, complete sequence
Cluster-33503.44194	--	226.954	57.551	1.9784	4.93E-05	0.011338	PREDICTED: Erinaceus europaeus keratin-associated protein 4-7-like (LOC103120622), mRNA
Cluster-29969.0	--	16.831	0.000	6.4722	4.96E-05	0.011411	PREDICTED: Capsicum annuum plastocyanin, chloroplastic (LOC107866833), mRNA
Cluster-4428.0	--	0.000	17.865	-6.6734	4.99E-05	0.01146	PREDICTED: Gossypium hirsutum late embryogenesis abundant protein Lea5-D (LOC107916177), mRNA
Cluster-33503.179583	--	0.000	20.564	-6.8757	5.00E-05	0.011468	--
Cluster-33503.93364	--	283.349	985.242	-1.7987	5.01E-05	0.011471	PREDICTED: Erinaceus europaeus jun B proto-oncogene (JUNB), mRNA
Cluster-8660.0	--	0.620	31.613	-5.6847	5.12E-05	0.011717	--
Cluster-33503.124801	--	33.659	1.345	4.636	5.13E-05	0.011743	Mus musculus BAC clone RP23-346N11 from chromosome 7, complete sequence
Cluster-33503.179672	--	8.955	83.230	-3.2208	5.18E-05	0.011832	Mus musculus genomic DNA, chromosome 4, clone:RP23-284M8, complete sequence >dbj|AP006506.1| Mus musculus genomic DNA, chromosome 4, clone: RP23-284M8, complete sequence
Cluster-33503.44569	--	18.704	1542.943	-6.3712	5.27E-05	0.012015	PREDICTED: Erinaceus europaeus keratin-associated protein 19-3-like (LOC103118358), mRNA

Cluster-33503.63829	--	8.722	77.232	-3.1068	5.27E-05	0.012015	PREDICTED: Erinaceus europaeus desmin (DES), mRNA
Cluster-6109.0	--	0.000	16.851	-6.5901	5.28E-05	0.012015	PREDICTED: Gossypium hirsutum FRIGIDA-like protein 4a (LOC107928523), mRNA
Cluster-33503.125562	--	52.225	1610.701	-4.9509	5.31E-05	0.012058	PREDICTED: Erinaceus europaeus keratin-associated protein 8-1 (LOC107523100), mRNA
Cluster-33503.66176	--	0.000	17.707	-6.666	5.36E-05	0.01215	--
Cluster-30447.0	--	25.923	0.399	6.1325	5.50E-05	0.012473	PREDICTED: Capsicum annuum GDSL esterase/lipase CPRD49-like (LOC107843709), mRNA
Cluster-33503.179347	--	4.081	48.947	-3.6616	5.51E-05	0.012476	MACACA MULATTA BAC clone CH250-118M20 from chromosome 17, complete sequence
Cluster-33503.121862	--	7.120	62.974	-3.1317	5.58E-05	0.012616	--
Cluster-33503.44849	--	742.412	2538.521	-1.7735	5.61E-05	0.012677	PREDICTED: Condylura cristata protein phosphatase 1, regulatory subunit 12B (PPP1R12B), transcript variant X5, mRNA
Cluster-71080.0	--	15.695	0.000	6.3739	5.69E-05	0.012836	PREDICTED: Capsicum annuum phospho-2-dehydro-3-deoxyheptonate aldolase 2, chloroplastic (LOC107850308), mRNA
Cluster-33503.27987	--	327.641	1885.784	-2.5269	5.84E-05	0.013138	PREDICTED: Erinaceus europaeus family with sequence similarity 26 member D (FAM26D), mRNA
Cluster-33503.112637	--	38.452	156.228	-2.0206	5.88E-05	0.013175	PREDICTED: Colobus angolensis palliatus tyrosine-protein phosphatase non-receptor type substrate 1 (LOC105512773), transcript variant X3, mRNA
Cluster-33503.94002	--	13959.797	333697.282	-4.5792	5.88E-05	0.013175	PREDICTED: Erinaceus europaeus keratin-associated protein 19-3-like (LOC103122994), transcript variant X1, mRNA
Cluster-3807.0	--	0.000	15.424	-6.4618	5.89E-05	0.013175	PREDICTED: Gossypium hirsutum uncharacterized LOC107893301 (LOC107893301), mRNA

Cluster-33503.71203	--	3.739	42.121	-3.5491	6.03E-05	0.013476	Atelerix albiventris clone LB4-443C20, complete sequence
Cluster-27408.0	--	15.555	0.000	6.3616	6.10E-05	0.013611	Capsicum annuum putative non-specific lipid transfer protein mRNA, complete cds
Cluster-33503.154754	--	6.617	73.662	-3.4226	6.13E-05	0.013674	PREDICTED: Erinaceus europaeus keratin, type II cuticular Hb1 (LOC103109277), mRNA
Cluster-33503.66165	--	16.660	96.485	-2.5552	6.13E-05	0.013675	PREDICTED: Erinaceus europaeus smoothelin (SMTN), transcript variant X2, mRNA
Cluster-33503.28399	--	98.571	11.366	3.1005	6.34E-05	0.01409	PREDICTED: Erinaceus europaeus keratin-associated protein 10-8-like (LOC103128195), mRNA
Cluster-33503.145149	--	0.799	28.342	-5.4007	6.35E-05	0.014105	--
Cluster-32183.0	--	16.869	0.000	6.4741	6.41E-05	0.014229	PREDICTED: Capsicum annuum peroxidase 42 (LOC107859584), mRNA
Cluster-33503.139467	--	53.280	235.754	-2.1449	6.73E-05	0.014911	PREDICTED: Erinaceus europaeus hephaestin like 1 (HEPHL1), mRNA
Cluster-33503.122761	--	13.272	115.202	-3.157	6.79E-05	0.015001	PREDICTED: Erinaceus europaeus keratin, type I cuticular Ha4-like (LOC103124756), mRNA
Cluster-33503.4439	--	0.000	14.697	-6.3918	6.81E-05	0.015034	Atelerix albiventris clone LB4-108K18, complete sequence
Cluster-33503.43352	--	304.930	2159.304	-2.8258	6.89E-05	0.015203	PREDICTED: Condylura cristata keratin-associated protein 19-2-like (LOC101625530), mRNA
Cluster-33503.66670	--	13.194	78.012	-2.5765	7.06E-05	0.015513	PREDICTED: Erinaceus europaeus desmin (DES), mRNA
Cluster-33503.122151	--	6230.505	143408.451	-4.5247	7.06E-05	0.01552	PREDICTED: Erinaceus europaeus keratin-associated protein 8-1 (LOC107523100), mRNA
Cluster-33503.68910	--	100.737	348.636	-1.7894	7.07E-05	0.01553	PREDICTED: Erinaceus europaeus junction plakoglobin (JUP), transcript variant X4, mRNA

Cluster-33503.82669	--	141.824	702.467	-2.304	7.12E-05	0.015612	PREDICTED: Erinaceus europaeus solute carrier family 2 member 1 (SLC2A1), transcript variant X2, mRNA
Cluster-4158.0	--	0.000	14.851	-6.4061	7.21E-05	0.015807	PREDICTED: Gossypium raimondii phospho-2-dehydro-3-deoxyheptonate aldolase 1, chloroplastic-like (LOC105769516), mRNA
Cluster-33503.127148	--	621.522	2271.509	-1.8692	7.30E-05	0.015984	PREDICTED: Canis lupus familiaris tensin 1 (TNS1), transcript variant X12, mRNA
Cluster-33503.49124	--	29.141	770.643	-4.7327	7.33E-05	0.016036	PREDICTED: Erinaceus europaeus keratin-associated protein 8-1 (LOC107523100), mRNA
Cluster-33503.127814	--	12348.786	44143.511	-1.8379	7.40E-05	0.016153	PREDICTED: Erinaceus europaeus heat shock protein family B (small) member 1 (HSPB1), mRNA
Cluster-33503.7984	--	0.000	14.724	-6.3946	7.39E-05	0.016153	PREDICTED: Erinaceus europaeus keratin, type II cytoskeletal 75-like (LOC103109183), mRNA
Cluster-4714.0	--	0.000	14.944	-6.4147	7.42E-05	0.016187	PREDICTED: Gossypium arboreum phenylalanine ammonia-lyase-like (LOC108467032), mRNA
Cluster-33503.146475	--	0.000	15.435	-6.458	7.61E-05	0.01655	PREDICTED: Erinaceus europaeus keratin-associated protein 8-1 (LOC107523100), mRNA
Cluster-3846.0	--	0.000	14.710	-6.3907	7.80E-05	0.016921	PREDICTED: Gossypium arboreum cytochrome b5-like (LOC108473809), mRNA
Cluster-33503.123828	--	14.857	87.288	-2.5909	7.93E-05	0.017187	PREDICTED: Erinaceus europaeus keratin-associated protein 4-11-like (LOC103120636), transcript variant X1, mRNA
Cluster-33503.7367	--	3.020	34.056	-3.426	7.97E-05	0.017261	Atelerix albiventris clone LB4-482N5, complete sequence
Cluster-33503.91002	--	16.754	111.627	-2.7405	8.10E-05	0.017524	--

Cluster-31058.0	--	15.751	0.000	6.3786	8.14E-05	0.017578	PREDICTED: Capsicum annuum photosystem II 5 kDa protein, chloroplastic-like (LOC107851418), mRNA
Cluster-33503.50141	--	0.400	20.711	-5.9214	8.20E-05	0.017706	PREDICTED: Erinaceus europaeus keratin-associated protein 7-1 (LOC103122995), mRNA
Cluster-33503.25903	--	77.177	15.254	2.3293	8.25E-05	0.017792	PREDICTED: Erinaceus europaeus family with sequence similarity 149 member A (FAM149A), mRNA
Cluster-33503.78629	--	38.763	162.493	-2.0741	8.26E-05	0.017792	--
Cluster-25902.0	--	14.697	0.000	6.2873	8.34E-05	0.017936	PREDICTED: Capsicum annuum uncharacterized LOC107845871 (LOC107845871), mRNA
Cluster-33503.124800	--	30.342	0.799	5.4022	8.40E-05	0.018043	--
Cluster-33503.338	--	4.099	39.934	-3.3375	8.41E-05	0.018049	PREDICTED: Erinaceus europaeus myelin regulatory factor-like (MYRFL), mRNA
Cluster-33503.66166	--	4.899	52.189	-3.319	8.50E-05	0.018191	PREDICTED: Erinaceus europaeus smoothelin (SMTN), transcript variant X2, mRNA
Cluster-75788.0	--	54.603	275.666	-2.3437	8.66E-05	0.018493	PREDICTED: Erinaceus europaeus serine/arginine repetitive matrix 3 (SRRM3), mRNA
Cluster-33503.37533	--	1.320	40.393	-4.7122	8.69E-05	0.018553	PREDICTED: Erinaceus europaeus sperm mitochondria associated cysteine rich protein (SMCP), mRNA
Cluster-33503.124546	--	1789.162	383.266	2.2231	8.71E-05	0.018569	PREDICTED: Erinaceus europaeus hornerin (HRNR), mRNA
Cluster-33503.152195	--	85.228	369.268	-2.1151	8.80E-05	0.018742	--
Cluster-27966.0	--	14.660	0.000	6.2786	8.85E-05	0.018825	PREDICTED: Capsicum annuum glycine-rich protein 3-like (LOC107866512), mRNA
Cluster-33503.28402	--	135.520	18.369	2.8901	8.92E-05	0.018948	--

Cluster-33503.34658	--	106.262	18.124	2.5651	9.06E-05	0.019185	PREDICTED: Erinaceus europaeus glycoprotein M6A (GPM6A), mRNA
Cluster-5012.0	--	0.000	14.198	-6.3433	9.48E-05	0.020011	PREDICTED: Gossypium arboreum L-ascorbate peroxidase 2, cytosolic (LOC108453402), mRNA
Cluster-5432.0	--	0.000	14.892	-6.408	9.60E-05	0.020178	PREDICTED: Gossypium hirsutum glutathione S-transferase U17-like (LOC107899606), mRNA
Cluster-33503.45623	--	10.215	72.271	-2.8445	9.62E-05	0.020195	Homo sapiens keratin 86 (KRT86), RefSeqGene on chromosome 12
Cluster-33503.27097	--	27.805	171.754	-2.6457	9.95E-05	0.020839	--
Cluster-33503.45771	--	7745.458	49776.016	-2.6841	9.95E-05	0.020839	--
Cluster-33503.24675	--	203.637	53.474	1.9333	0.00010151	0.02122	PREDICTED: Erinaceus europaeus interferon-induced very large GTPase 1-like (LOC103117645), transcript variant X2, mRNA
Cluster-33503.27901	--	108.072	531.313	-2.3016	0.00010222	0.021335	--
Cluster-33503.68660	--	2.340	40.184	-3.9552	0.00010396	0.021637	--
Cluster-33503.80965	--	82.609	418.869	-2.34	0.00010428	0.021665	Atelerix albiventris clone LB4-495B20, complete sequence
Cluster-33503.81903	--	54.458	214.526	-1.9842	0.00010504	0.021806	PREDICTED: Erinaceus europaeus jun B proto-oncogene (JUNB), mRNA
Cluster-55585.0	--	0.000	14.558	-6.375	0.00010708	0.02218	PREDICTED: Gossypium raimondii subtilisin-like protease SBT1.7 (LOC105798729), mRNA
Cluster-3107.2	--	0.000	13.557	-6.2784	0.00010717	0.022181	Atelerix albiventris clone LB4-498C22, complete sequence
Cluster-33503.108452	--	142.542	615.833	-2.1128	0.00010765	0.022253	--
Cluster-33503.52871	--	669.954	2601.533	-1.9577	0.00010953	0.022618	Erinaceus europaeus, clone XX-29935257F24, complete sequence
Cluster-33503.46032	--	5.775	64.055	-3.5445	0.00010973	0.022624	Atelerix albiventris clone LB4-244I20, complete sequence

Cluster-5274.0	--	0.000	15.300	-6.4496	0.00010972	0.022624	Gossypium hirsutum flavonoid 3',5'-hydroxylase 2-like (LOC107953189), mRNA >gb|EU921266.1| Gossypium hirsutum cultivar Xincai 5 flavonoid 3'5'-hydroxylase mRNA, complete cds
Cluster-33503.128469	--	0.000	14.071	-6.326	0.00010999	0.022658	PREDICTED: Erinaceus europaeus keratin-associated protein 19-2-like (LOC103118359), mRNA
Cluster-33503.125983	--	0.220	25.180	-6.2121	0.00011031	0.022692	PREDICTED: Erinaceus europaeus keratin, type II cytoskeletal 75-like (LOC103109183), mRNA
Cluster-33503.182124	--	0.000	14.132	-6.3373	0.00011064	0.022742	Macaca fascicularis complete genome, chromosome chr2
Cluster-33503.106952	--	0.000	13.785	-6.2983	0.00011107	0.022797	PREDICTED: Erinaceus europaeus carbohydrate sulfotransferase 1 (CHST1), mRNA
Cluster-99085.0	--	14.494	0.000	6.2565	0.00011107	0.022797	PREDICTED: Capsicum annuum translationally-controlled tumor protein homolog (LOC107839623), mRNA
Cluster-33503.132648	--	2.162	35.894	-4.1351	0.00011238	0.023031	PREDICTED: Erinaceus europaeus calponin 1 (CNN1), transcript variant X2, mRNA
Cluster-33503.28386	--	0.440	30.309	-5.7649	0.00011436	0.023401	--
Cluster-33503.30630	--	59.161	9.507	2.6118	0.00011614	0.023731	--
Cluster-33503.126355	--	0.721	24.070	-5.209	0.00011707	0.023901	PREDICTED: Erinaceus europaeus keratin-associated protein 15-1 (LOC103118357), mRNA
Cluster-33503.91949	--	31.696	130.815	-2.0308	0.00011776	0.024025	Erinaceus europaeus, clone XX-29460857F24, complete sequence
Cluster-73121.0	--	14.155	0.000	6.2303	0.00011859	0.024177	PREDICTED: Capsicum annuum uncharacterized LOC107877050 (LOC107877050), transcript variant X3, mRNA
Cluster-33503.124222	--	1555.483	4760.476	-1.6138	0.0001195	0.024344	PREDICTED: Erinaceus europaeus keratin, type I cytoskeletal 25 (LOC103120609), mRNA

Cluster-33503.135808	--	289.243	1404.649	-2.28	0.00011997	0.024421	PREDICTED: Erinaceus europaeus protein S100-A15A-like (LOC103115638), mRNA
Cluster-33503.161437	--	115.170	21.740	2.4097	0.00012037	0.024484	PREDICTED: Erinaceus europaeus leupaxin (LPXN), mRNA
Cluster-33503.119353	--	35.654	164.355	-2.2139	0.00012079	0.024532	--
Cluster-114330.0	--	0.000	14.225	-6.3412	0.00012121	0.024591	PREDICTED: Gossypium raimondii aquaporin PIP1-3-like (LOC105792230), mRNA
Cluster-33503.25504	--	0.000	14.388	-6.3583	0.00012196	0.024715	PREDICTED: Erinaceus europaeus keratin-associated protein 15-1 (LOC103118357), mRNA
Cluster-33503.27180	--	335.500	66.307	2.3352	0.00012623	0.02551	PREDICTED: Erinaceus europaeus GLI family zinc finger 1 (GLI1), transcript variant X3, mRNA
Cluster-53373.0	--	1.679	27.522	-3.9701	0.00012626	0.02551	PREDICTED: Myotis davidii uncharacterized LOC107182631 (LOC107182631), ncRNA
Cluster-33503.129199	--	57.979	289.850	-2.3314	0.0001264	0.025517	--
Cluster-33503.49022	--	10.735	73.017	-2.8062	0.00012648	0.025517	PREDICTED: Monodelphis domestica keratin 86 (KRT86), mRNA
Cluster-27665.0	--	13.799	0.000	6.1927	0.00012669	0.02554	Capsicum annuum glycine-rich RNA-binding protein 1 (GRP1) gene, complete cds, alternatively spliced
Cluster-33503.43926	--	341.420	6555.246	-4.2637	0.00012813	0.02581	PREDICTED: Erinaceus europaeus keratin-associated protein 8-1 (LOC107523100), mRNA
Cluster-33503.126302	--	92.322	479.044	-2.38	0.00012844	0.025853	Ovis aries keratin intermediate filament type II (KRT2.10) gene, partial cds
Cluster-33503.170568	--	12.898	81.380	-2.634	0.00012882	0.025896	Atelerix albiventris clone LB4-244I20, complete sequence
Cluster-33503.178803	--	2.399	31.360	-3.7766	0.00012884	0.025896	Erinaceus europaeus, clone XX-29460857F24, complete sequence

Cluster-33503.95845	--	485.338	1537.155	-1.6628	0.00012918	0.025946	PREDICTED: Erinaceus europaeus cysteine rich secretory protein LCCL domain containing 2 (CRISPLD2), transcript variant X2, mRNA
Cluster-88493.0	--	14.214	0.000	6.2298	0.00013012	0.026096	PREDICTED: Capsicum annuum photosystem I reaction center subunit III, chloroplastic-like (LOC107858989), mRNA
Cluster-33503.54219	--	169.954	675.755	-1.9943	0.0001305	0.026152	PREDICTED: Erinaceus europaeus Cbp/p300 interacting transactivator with Glu/Asp rich carboxy-terminal domain 4 (CITED4), mRNA
Cluster-33503.7005	--	0.000	13.424	-6.2597	0.00013179	0.026391	Pongo abelii mRNA; cDNA DKFZp469H1115 (from clone DKFZp469H1115)
Cluster-33503.178673	--	4.180	37.155	-3.1476	0.00013278	0.02657	Atelerix albiventris clone LB4-108K18, complete sequence
Cluster-33503.149665	--	181.361	726.167	-2.0031	0.0001329	0.026574	PREDICTED: Erinaceus europaeus argininosuccinate synthase 1 (ASS1), mRNA
Cluster-33503.106725	--	130.555	627.696	-2.2664	0.00013445	0.026826	PREDICTED: Erinaceus europaeus solute carrier family 9 member B2 (SLC9B2), mRNA
Cluster-33503.9350	--	0.799	110.907	-7.2567	0.00013665	0.027243	PREDICTED: Erinaceus europaeus keratin-associated protein 19-2-like (LOC103118359), mRNA
Cluster-33503.147794	--	30.119	170.194	-2.4996	0.00013721	0.027316	PREDICTED: Erinaceus europaeus dual specificity phosphatase 5 (DUSP5), mRNA
Cluster-29957.0	--	14.751	0.000	6.2789	0.00013946	0.027722	PREDICTED: Capsicum annuum photosystem II core complex proteins psbY, chloroplastic (LOC107844032), mRNA
Cluster-33503.68998	--	1.199	27.252	-4.715	0.00014164	0.028136	PREDICTED: Erinaceus europaeus keratin, type II cytoskeletal 6A (LOC103109181), mRNA
Cluster-33503.43347	--	3195.294	18278.864	-2.5163	0.00014284	0.028347	--
Cluster-33503.26238	--	0.000	13.225	-6.2403	0.00014377	0.028455	--

Cluster-33503.50178	--	394.222	74.764	2.3979	0.00014726	0.029102	PREDICTED: Erinaceus europaeus loricrin (LOR), mRNA
Cluster-33503.124423	--	4.357	65.530	-3.9662	0.00014788	0.029205	--
Cluster-33503.41824	--	35.739	176.884	-2.325	0.00015208	0.029946	PREDICTED: Erinaceus europaeus keratin, type II cuticular Hb6 (LOC103109202), mRNA
Cluster-33503.47914	--	35.739	176.884	-2.325	0.00015208	0.029946	PREDICTED: Erinaceus europaeus keratin, type II cuticular Hb6 (LOC103109202), mRNA
Cluster-33503.5744	--	62.801	325.837	-2.3743	0.0001528	0.030031	PREDICTED: Marmota marmota marmota uncharacterized LOC107140567 (LOC107140567), ncRNA
Cluster-33503.125010	--	28.711	2.224	3.6388	0.00015389	0.030215	PREDICTED: Erinaceus europaeus keratin-associated protein 12-1-like (LOC103111332), mRNA
Cluster-35912.0	--	14.033	0.000	6.2105	0.00015454	0.030321	PREDICTED: Capsicum annuum metallothionein-like protein type 2 (LOC107875536), mRNA
Cluster-33503.7975	--	11.716	93.032	-2.9595	0.00015507	0.030382	Atelerix albiventris clone LB4-477I10, complete sequence
Cluster-31080.11	--	14.431	0.000	6.2488	0.00015621	0.030516	PREDICTED: Capsicum annuum chlorophyll a-b binding protein 21, chloroplastic-like (LOC107855780), mRNA
Cluster-33503.6725	--	1.200	22.165	-4.1928	0.00015618	0.030516	Erinaceus europaeus, clone XX-29460857F24, complete sequence
Cluster-33503.45701	--	265.516	4973.553	-4.2283	0.00015734	0.030649	PREDICTED: Erinaceus europaeus keratin-associated protein 8-1 (LOC107523100), mRNA
Cluster-64741.0	--	13.415	0.000	6.1497	0.00016352	0.031808	PREDICTED: Capsicum annuum sedoheptulose-1,7-bisphosphatase, chloroplastic (LOC107871105), mRNA
Cluster-33503.47257	--	357.950	1072.198	-1.5836	0.00016538	0.032146	PREDICTED: Erinaceus europaeus AT-rich interaction domain 3A (ARID3A), mRNA

Cluster-112947.0	--	0.400	18.791	-5.7773	0.00016556	0.032158	Atelerix albiventris clone LB4-416G11, complete sequence
Cluster-33503.7635	--	1.819	31.370	-4.0988	0.00016658	0.032333	PREDICTED: Erinaceus europaeus transient receptor potential cation channel subfamily V member 1 (TRPV1), mRNA
Cluster-33503.28909	--	242.652	864.786	-1.8323	0.00016777	0.032541	Erinaceus europaeus, clone XX-29438057B6, complete sequence
Cluster-33503.35606	--	15.396	72.434	-2.2217	0.00016809	0.032579	Ovis canadensis canadensis isolate 43U chromosome 11 sequence
Cluster-33503.23074	--	801.474	16965.088	-4.404	0.0001683	0.032597	PREDICTED: Erinaceus europaeus keratin-associated protein 14-like (LOC103118356), mRNA
Cluster-33503.45904	--	0.620	26.361	-5.4225	0.00016868	0.032604	PREDICTED: Myotis davidii cornifin alpha (CORNIFIN-A), transcript variant X21, mRNA
Cluster-33503.6880	--	1.239	28.228	-4.5216	0.00017097	0.032997	Atelerix albiventris clone LBNL4-89B6, complete sequence
Cluster-33503.123580	--	259.545	735.928	-1.5033	0.00017131	0.033038	Atelerix albiventris clone LB4-244I20, complete sequence
Cluster-33503.125075	--	30.582	2.896	3.3697	0.00017248	0.03324	PREDICTED: Erinaceus europaeus keratin-associated protein 12-1-like (LOC103111332), mRNA
Cluster-27038.0	--	13.895	0.000	6.1917	0.00017478	0.033661	PREDICTED: Capsicum annuum glycine-rich cell wall structural protein 2-like (LOC107855327), mRNA
Cluster-33503.44850	--	132.407	458.352	-1.7877	0.00017553	0.03378	--
Cluster-33503.50135	--	3.040	35.773	-3.6013	0.00017655	0.033928	PREDICTED: Erinaceus europaeus keratin-associated protein 7-1 (LOC103122995), mRNA
Cluster-33503.50005	--	636.372	188.752	1.7516	0.00017698	0.033987	PREDICTED: Erinaceus europaeus keratin 10 (KRT10), mRNA
Cluster-33503.75894	--	39.122	3.342	3.6019	0.00018264	0.03505	Atelerix albiventris clone LB4-443C20, complete sequence

Cluster-33503.43354	--	306.085	1855.436	-2.6016	0.00018312	0.035109	--
Cluster-33503.59	--	0.220	19.487	-5.8372	0.00018407	0.035249	--
Cluster-4669.0	--	0.000	12.718	-6.1819	0.00018496	0.035395	Homo sapiens chromosome 8, clone RP11-26K8, complete sequence
Cluster-33503.28404	--	28.212	0.912	4.8482	0.00018527	0.035429	--
Cluster-33503.133160	--	137.536	24.682	2.4819	0.00018669	0.035662	PREDICTED: Erinaceus europaeus mast cell-expressed membrane protein 1 (MCEMP1), mRNA
Cluster-33503.48861	--	3030.137	729.165	2.0552	0.00018675	0.035662	PREDICTED: Erinaceus europaeus complement component 1, q subcomponent, B chain (C1QB), mRNA
Cluster-33503.5242	--	1.779	25.713	-3.9602	0.00019073	0.036395	Atelerix albiventris clone LB4-197A24, complete sequence
Cluster-33503.42087	--	16.193	94.478	-2.5471	0.00019094	0.03641	Atelerix albiventris clone LB4-171F3, complete sequence
Cluster-33503.22297	--	262.534	788.050	-1.5868	0.00019326	0.036827	PREDICTED: Erinaceus europaeus family with sequence similarity 167 member A (FAM167A), mRNA
Cluster-33503.7609	--	11.651	326.787	-4.8293	0.0001941	0.03696	Bos taurus isolate Dominette_000065F genomic sequence
Cluster-33503.123838	--	629.412	2367.314	-1.9123	0.00019504	0.037114	PREDICTED: Erinaceus europaeus keratin-associated protein 4-11-like (LOC103120636), transcript variant X1, mRNA
Cluster-23173.2	--	0.000	12.650	-6.1773	0.0001977	0.037519	--
Cluster-33503.61616	--	462.425	1291.435	-1.4821	0.00019734	0.037519	Atelerix albiventris clone LB4-197A24, complete sequence
Cluster-33503.74244	--	315.927	919.062	-1.5411	0.00019754	0.037519	PREDICTED: Erinaceus europaeus pyruvate dehydrogenase kinase 3 (PDK3), transcript variant X2, mRNA

Cluster-98002.0	--	13.773	0.000	6.1929	0.00019772	0.037519	PREDICTED: Capsicum annuum auxin-repressed 12.5 kDa protein (LOC107839631), mRNA
Cluster-33503.151796	--	162.652	566.134	-1.803	0.0001987	0.037678	PREDICTED: Erinaceus europaeus family with sequence similarity 110 member C (FAM110C), mRNA
Cluster-33503.124215	--	9.493	89.632	-3.2613	0.0001998	0.037835	PREDICTED: Nomascus leucogenys keratin-associated protein 19-3 (LOC105738720), transcript variant X2, mRNA
Cluster-33503.125662	--	7320.935	2149.224	1.7681	0.00020231	0.038202	PREDICTED: Erinaceus europaeus keratin 10 (KRT10), mRNA
Cluster-33503.28246	--	247.963	4555.783	-4.2004	0.00020675	0.038878	PREDICTED: Erinaceus europaeus keratin-associated protein 8-1 (LOC107523100), mRNA
Cluster-33503.44568	--	8.472	519.108	-5.9518	0.00020829	0.039142	PREDICTED: Erinaceus europaeus keratin-associated protein 19-2-like (LOC103118359), mRNA
Cluster-101969.0	--	13.634	0.000	6.1811	0.00020952	0.039318	PREDICTED: Capsicum annuum beta-carotene hydroxylase 2, chloroplastic (LOC107873401), transcript variant X1, mRNA
Cluster-82403.0	--	120.454	24.791	2.2926	0.00021013	0.039406	PREDICTED: Erinaceus europaeus Fc receptor like A (FCRLA), mRNA
Cluster-33503.107454	--	89.832	358.236	-2.0037	0.00021074	0.039492	PREDICTED: Erinaceus europaeus transgelin (TAGLN), mRNA
Cluster-33503.11069	--	89.413	337.788	-1.9196	0.00021107	0.039527	PREDICTED: Erinaceus europaeus dendrin (DDN), mRNA
Cluster-26131.0	--	14.210	0.000	6.2245	0.00021365	0.039936	Solanum nigrum metallothionein (MT3b) mRNA, complete cds
Cluster-33503.12821	--	35.682	162.634	-2.1948	0.00021399	0.039964	PREDICTED: Erinaceus europaeus carbonic anhydrase 2 (CA2), mRNA

Cluster-33503.126329	--	332.495	47.617	2.8104	0.00021543	0.040178	PREDICTED: Erinaceus europaeus myosin-1 (LOC103113192), transcript variant X2, mRNA
Cluster-33503.48578	--	27.042	0.672	5.3201	0.00021595	0.040246	PREDICTED: Erinaceus europaeus tryptase alpha/beta-1-like (LOC107522796), transcript variant X2, mRNA
Cluster-33503.61031	--	126.457	440.100	-1.7962	0.00021633	0.040262	PREDICTED: Equus asinus synemin (SYNM), mRNA
Cluster-33503.129744	--	0.799	93.751	-7.0155	0.00021666	0.040296	PREDICTED: Erinaceus europaeus keratin-associated protein 13-1-like (LOC103118345), mRNA
Cluster-33503.50001	--	410.138	104.293	1.9749	0.00022148	0.041136	PREDICTED: Erinaceus europaeus keratin 10 (KRT10), mRNA
Cluster-75350.0	--	0.000	12.960	-6.2082	0.00022275	0.041344	PREDICTED: Gossypium hirsutum BAHD acyltransferase DCR-like (LOC107917823), mRNA
Cluster-33503.62347	--	951.050	3223.066	-1.7609	0.0002249	0.041714	PREDICTED: Microcebus murinus desmoplakin (DSP), transcript variant X1, mRNA
Cluster-33503.49622	--	16.515	93.690	-2.497	0.00022586	0.041812	PREDICTED: Chrysochloris asiatica sperm mitochondrial-associated cysteine-rich protein-like (LOC102815696), mRNA
Cluster-71261.1	--	58.064	7.757	2.8982	0.00022899	0.04229	PREDICTED: Erinaceus europaeus adenosine receptor A3 (LOC103116970), mRNA
Cluster-33503.79304	--	0.360	18.590	-5.7609	0.00023218	0.042802	Atelerix albiventris clone LB4-341B4, complete sequence
Cluster-33503.91019	--	49.116	188.895	-1.9393	0.000233	0.042924	Atelerix albiventris clone LB4-57B20, complete sequence
Cluster-33503.81901	--	299.320	967.730	-1.6914	0.00023434	0.043142	PREDICTED: Marmota marmota marmota jun B proto-oncogene (Junb), mRNA
Cluster-33503.2396	--	255.200	43.090	2.5656	0.00023475	0.043187	Homo sapiens BAC clone RP11-384O8 from 2, complete sequence

Cluster-76701.0	--	13.751	0.000	6.1811	0.00023552	0.043299	PREDICTED: Capsicum annuum oxygen-evolving enhancer protein 2, chloroplastic (LOC107856476), mRNA
Cluster-33503.24383	--	22.809	117.508	-2.3597	0.00023586	0.043333	PREDICTED: Erinaceus europaeus keratin, type II microfibrillar, component 7C (LOC103109188), mRNA
Cluster-33503.56689	--	105.857	466.906	-2.1481	0.0002398	0.043997	--
Cluster-63594.0	--	4.838	40.334	-3.0633	0.00024027	0.044054	PREDICTED: Erinaceus europaeus dedicator of cytokinesis 2 (DOCK2), mRNA
Cluster-49082.0	--	0.000	12.581	-6.1681	0.00024144	0.044239	PREDICTED: Gossypium arboreum peroxidase 42 (LOC108453239), mRNA
Cluster-33503.181824	--	0.000	12.410	-6.1488	0.00024193	0.044298	Atelerix albiventris clone LB4-495B20, complete sequence
Cluster-33503.133096	--	389.989	1330.867	-1.7705	0.00024319	0.044469	PREDICTED: Camelus ferus sarcolemma associated protein (SLMAP), transcript variant X12, mRNA
Cluster-33503.31464	--	77.188	405.620	-2.395	0.00024316	0.044469	--
Cluster-33503.142084	--	3.219	44.107	-3.68	0.00024908	0.045454	Atelerix albiventris clone LB4-171F3, complete sequence
Cluster-33503.125193	--	6.574	267.052	-5.3709	0.00025189	0.045936	PREDICTED: Erinaceus europaeus keratin-associated protein 8-1 (LOC107523100), mRNA
Cluster-33503.134458	--	0.721	22.915	-5.1324	0.00025474	0.046425	PREDICTED: Erinaceus europaeus myosin light chain kinase (MYLK), transcript variant X3, mRNA
Cluster-33503.81902	--	47.277	181.914	-1.9511	0.00025511	0.046461	PREDICTED: Erinaceus europaeus jun B proto-oncogene (JUNB), mRNA
Cluster-71688.0	--	34.078	0.766	5.5747	0.00025587	0.046536	PREDICTED: Erinaceus europaeus T-cell receptor-associated transmembrane adapter 1 (LOC103112706), mRNA

Cluster-33503.181996	--	175.913	26.204	2.7371	0.0002568	0.046643	PREDICTED: Erinaceus europaeus thyroid hormone responsive (THRSP), mRNA
Cluster-33503.24966	--	15.709	474.001	-4.9276	0.00025988	0.047171	PREDICTED: Erinaceus europaeus keratin-associated protein 8-1 (LOC107523100), mRNA
Cluster-33503.179527	--	37.407	186.399	-2.3328	0.00026168	0.047435	Erinaceus europaeus, clone XX-29460857F24, complete sequence
Cluster-33503.125513	--	19.030	100.358	-2.4209	0.00026217	0.047436	PREDICTED: Erinaceus europaeus phospholipase A2 inhibitor and LY6/PLAUR domain containing (PINLYP), mRNA
Cluster-33503.17148	--	2.899	35.803	-3.7031	0.00026221	0.047436	Atelerix albiventris clone LB4-244I20, complete sequence
Cluster-33503.49151	--	0.360	19.666	-5.8406	0.00026196	0.047436	PREDICTED: Erinaceus europaeus keratin-associated protein 8-1 (LOC107523100), mRNA
Cluster-33503.143899	--	110.537	24.124	2.1923	0.0002644	0.0478	PREDICTED: Erinaceus europaeus delta/notch like EGF repeat containing (DNER), mRNA
Cluster-33503.28241	--	1.159	29.019	-4.8056	0.0002661	0.048044	PREDICTED: Erinaceus europaeus keratin-associated protein 8-1 (LOC107523100), mRNA
Cluster-33503.123833	--	86.453	315.286	-1.8743	0.00026657	0.048096	PREDICTED: Erinaceus europaeus keratin-associated protein 4-11-like (LOC103120636), transcript variant X1, mRNA
Cluster-33503.117769	--	154.033	617.515	-2.0079	0.00027132	0.048889	--
Cluster-33503.179868	--	1.199	23.164	-4.4861	0.00027186	0.048948	PREDICTED: Erinaceus europaeus calponin 1 (CNN1), transcript variant X2, mRNA
Cluster-40520.0	--	3.358	42.413	-3.6235	0.00027201	0.048948	--
Cluster-33503.55896	--	6.899	41.306	-2.592	0.00027369	0.049218	PREDICTED: Erinaceus europaeus absent in melanoma 1 (AIM1), mRNA
Cluster-33503.179549	--	2.399	26.876	-3.5624	0.0002739	0.049224	--

Cluster-33503.11058	--	63.825	243.651	-1.9337	0.00027524	0.049399	PREDICTED: Erinaceus europaeus stimulated by retinoic acid 6 (STRA6), mRNA
Cluster-33503.81273	--	45.131	183.695	-2.0324	0.0002766	0.049544	PREDICTED: Erinaceus europaeus tropomyosin 2 (beta) (TPM2), transcript variant X4, mRNA
Cluster-33503.152479	--	3.018	34.641	-3.567	0.00027895	0.049932	Atelerix albiventris clone LB4-372K23, complete sequence

Supplementary table 6 NCBI accession numbers of *KRT1* sequences from 19 species

Species	NCBI accession number
Erinaceus europaeus	XM_007535509.2
Odobenus rosmarus	XM_004410492.1
Ursus arctos	XM_026491053.1
Ursus maritimus	XM_008701257.1
Ailuropoda melanoleuca	XM_011228597.2
Canis lupus	XM_025440552.1
Vulpes vulpes	XM_025987081.1
Mustela putorius	XM_004772755.2
Rousettus aegyptiacus	XM_016150993.1
Equus asinus	XM_014833554.1
Callorhinus ursinus	XM_025861805.1
Condylura cristata	XM_004684201.2
Lontra canadensis	XM_032871791.1
Phoca vitulina	XM_032396498.1
Zalophus californianus	>XM_027625808.1
Felis catus	XM_006940344.3
Tupaia chinensis	XM_006155972.3
Trichechus manatus	XM_004390333.2
Callithrix jacchus	XM_002748619.3
